# Pursuing the Complexity of Alzheimer’s Disease: Discovery of Fluoren-9-Amines as Selective Butyrylcholinesterase Inhibitors and *N*-Methyl-d-Aspartate Receptor Antagonists

**DOI:** 10.3390/biom11010003

**Published:** 2020-12-22

**Authors:** Jan Konecny, Anna Misiachna, Martina Hrabinova, Lenka Pulkrabkova, Marketa Benkova, Lukas Prchal, Tomas Kucera, Tereza Kobrlova, Vladimir Finger, Marharyta Kolcheva, Stepan Kortus, Daniel Jun, Marian Valko, Martin Horak, Ondrej Soukup, Jan Korabecny

**Affiliations:** 1 Department of Toxicology and Military Pharmacy, Faculty of Military Health Sciences, Trebesska 1575, 500 01 Hradec Kralove, Czech Republic; jan.konecny@unob.cz (J.K.); martina.hrabinova@unob.cz (M.H.); lenka.pulkrabkova@fnhk.cz (L.P.); tomas.kucera2@unob.cz (T.K.); daniel.jun@unob.cz (D.J.); 2 Biomedical Research Centre, University Hospital Hradec Kralove, Sokolska 581, 500 05 Hradec Kralove, Czech Republic; Marketa.Benkova@fnhk.cz (M.B.); lukas.prchal@fnhk.cz (L.P.); tereza.kobrlova@fnhk.cz (T.K.); fingerv@faf.cuni.cz (V.F.); 3 Institute of Experimental Medicine of the Czech Academy of Sciences, Videnska 1083, 142 20 Prague, Czech Republic; anna.misiachna@iem.cas.cz (A.M.); marharyta.kolcheva@iem.cas.cz (M.K.); stepan.kortus@iem.cas.cz (S.K.); martin.horak@iem.cas.cz (M.H.); 4 Institute of Physiology of the Czech Academy of Sciences, Videnska 1083, 142 20 Prague, Czech Republic; 5 Department of Physiology, Faculty of Science, Charles University in Prague, Albertov 6, 128 43 Prague, Czech Republic; 6 Department of Organic and Bioorganic Chemistry, Faculty of Pharmacy in Hradec Kralove, Charles University, Akademika Heyrovskeho 1203, 500 05 Hradec Kralove, Czech Republic; 7 Faculty of Chemical and Food Technology, Slovak University of Technology, Radlinskeho 9, 812 37 Bratislava, Slovakia; marian.valko@stuba.sk

**Keywords:** acetylcholinesterase, Alzheimer’s disease, butyrylcholinesterase, fluorene, in vitro, in silico, multi-target directed ligands, *N*-methyl-d-aspartate receptor

## Abstract

Alzheimer’s disease (AD) is a complex disorder with unknown etiology. Currently, only symptomatic therapy of AD is available, comprising cholinesterase inhibitors and *N*-methyl-d-aspartate (NMDA) receptor antagonists. Drugs targeting only one pathological condition have generated only limited efficacy. Thus, combining two or more therapeutic interventions into one molecule is believed to provide higher benefit for the treatment of AD. In the presented study, we designed, synthesized, and biologically evaluated 15 novel fluoren-9-amine derivatives. The in silico prediction suggested both the oral availability and permeation through the blood–brain barrier (BBB). An initial assessment of the biological profile included determination of the cholinesterase inhibition and NMDA receptor antagonism at the GluN1/GluN2A and GluN1/GluN2B subunits, along with a low cytotoxicity profile in the CHO-K1 cell line. Interestingly, compounds revealed a selective butyrylcholinesterase (BChE) inhibition pattern with antagonistic activity on the NMDARs. Their interaction with butyrylcholinesterase was elucidated by studying enzyme kinetics for compound **3c** in tandem with the in silico docking simulation. The docking study showed the interaction of the tricyclic core of new derivatives with Trp82 within the anionic site of the enzyme in a similar way as the template drug tacrine. From the kinetic analysis, it is apparent that **3c** is a competitive inhibitor of BChE.

## 1. Introduction

Alzheimer’s disease (AD) is a debilitating neurodegenerative disorder that manifests as progressive memory loss leading to dementia [1,2]. AD not only represents a serious health burden, but it also imposes social and economic issues [3,4,5,6]. Worldwide, nearly 50 million people have developed AD, and it is expected that the number will double within the next 20 years [7]. An effective cure able to halt or slow down the disease progression still does not exist, mainly because of our limited knowledge about AD pathophysiology. However, mechanisms resulting in the clinical symptoms of AD are well-established [8,9]. Among them, amyloid-β (Aβ) and tau proteins are considered as major contributors to AD, forming extracellular aggregates [10,11] and intracellular neurofibrillary tangles, respectively [12,13,14]. So far, the therapy is only palliative, either enhancing the cholinergic neurotransmission or modulating the synaptic excitotoxicity via *N*-methyl-d-aspartate (NMDA) receptors [15,16,17,18]. Oxidative stress [19,20], metal ion imbalance [21], or neuroinflammation [22] are other crucial players in AD pathophysiology.

Currently, AD is treated by acetylcholinesterase (AChE, E.C. 3.1.1.7) inhibitors to restore the physiological levels of acetylcholine (ACh) and the NMDA receptor antagonist, which reduce the excitotoxicity by mitigating the excessive glutamate stimulation of the receptors [23,24]. Donepezil, galantamine, and rivastigmine represent the marketed drugs from the group of AChE inhibitors; memantine acts as a noncompetitive NMDA receptor antagonist, blocking the overstimulation of the respective receptors. Since AChE inhibitors are administered in mild-to-moderate stages of AD, memantine is indicated for severe stages of the disease [25]. It is worth mentioning that the combination of donepezil and memantine into one capsule, known as Namzaric, was approved in 2014. Such a combination offers an improved efficacy compared to single-agent administration with a good pharmacokinetic profile, safety, and tolerability [26,27].

Given the complexity of AD and the high failure rate of single-targeted drug candidates from clinical trials in the last few years, the idea of multitarget directed ligands (MTDLs) has emerged as a new approach to tackle the disease [28,29,30,31]. Accordingly, the design of these molecules is oriented towards the modulation of different pathological pathways simultaneously [29,32]. Recently, we have critically pointed out that not all the combined effects are suitable to be amalgamated into a single molecule to achieve the highest therapeutic effect [29]. Indeed, AD is a long-term condition with progressive brain changes occurring 20 years before the AD outbreak into the symptomatic phase [33]. With respect to the combination of therapeutic targets of interest and to achieve maximum synergy, the ideal drugs should be designed by following the timescale for specific pathological cascades. Within this study, we have focused on the symptomatic stage of AD, in which Aβ plaques and neurofibrillary tangles of paired helical filaments are ubiquitous, and their clearance has no effect on improving cognitive functions. This is well-documented, for instance, by the insufficient efficacy of β-secretase inhibitors in phase III clinical trials [34]. With this in mind, we turned our attention to impaired neurotransmission, which is the most critically affected at the later stages of AD, thus pursuing the two most pronounced systems, namely cholinergic and glutamatergic ones [35,36].

Tacrine (THA), an AChE inhibitor, was the first drug approved for AD treatment in 1993. Hepatotoxicity and gastrointestinal discomfort are the culprits responsible for its withdrawal in 2013 [37]. THA acts not only as AChE inhibitor but, also, via the antagonism of NMDA receptors, which might contribute to its pharmacological effects [38]. Indeed, THA inhibits NMDA receptor responses with high specificity in a concentration-dependent manner with an IC_50_ = 20 μM at −60 mV and much higher values at positive membrane potentials [39]. Mechanistically, the THA action at NMDA receptors can be classified as reversible, blocking the channel’s open state with binding in the proximity of the channel entrance [40,41]. Likewise, other THA derivatives emerged as interesting drugs with a potential applicability for AD treatment due to the pharmacological behavior of both cholinesterases and the NMDA receptor [39,42]. Based on this, our goal was to design a series of novel multipotent agents, which, in one molecule, shows a representative cholinesterase inhibition together with NMDA antagonist activity. 

**Figure 1:**
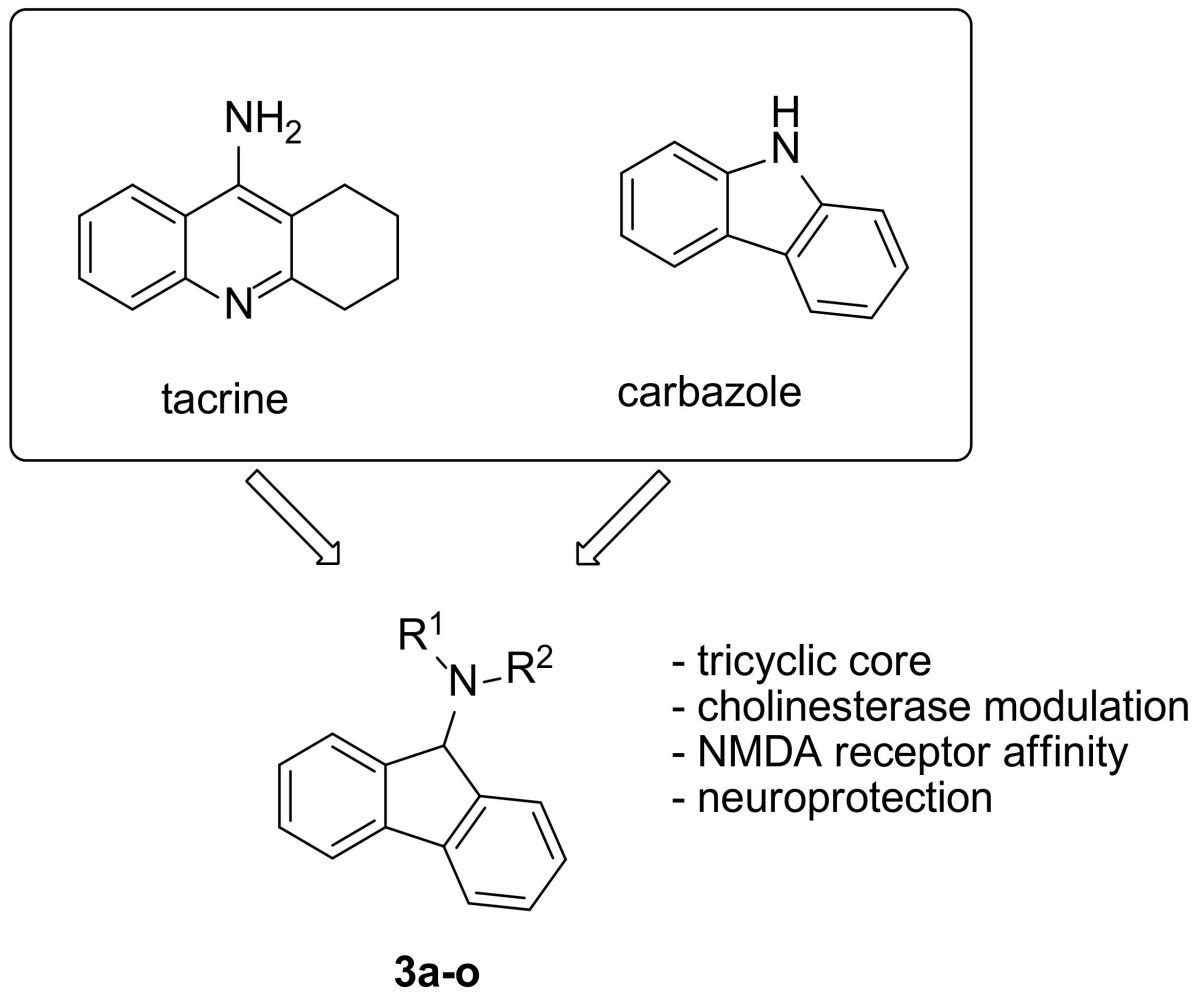
Design of a novel 9-aminofluorene overlapping the tacrine (THA) and carbazole moieties. NMDA: *N*-methyl-d-aspartate.

In a recent work, we developed novel molecules building on a fluorene moiety. The compounds are structurally related to other tricyclic congeners like THA or carbazole, known for their cholinesterase inhibition and/or neuroprotective properties (Figure 1) [43,44,45]. We took advantage of the above-mentioned and designed novel molecules substituted at the C9 position by various primary or secondary amines, generating 15 novel compounds (**3a**–**o**; Figure 1) as potential cholinesterase inhibitors and NMDA receptor antagonists. Importantly, since several techniques on how to construct MTDLs exist, we applied the so-called merging approach in this study. This approach best corresponds to the drug-likeness of the final molecule compared to the linking or fusing strategies [28,29,32]. Furthermore, the design of the novel family was envisaged in parallel with their physicochemical properties, presuming both oral and central bioavailability. The biological profile includes the assessment of AChE, butyrylcholinesterase (BChE; E.C. 3.1.1.8), and NMDA receptor affinities, with the cytotoxicity and BBB permeation being evaluated as well.

## 2. Materials and Methods

### 2.1. Chemistry

All chemical solvents and reagents were used in the highest available purity without further purification, and they were purchased from Sigma-Aldrich (Prague, Czech Republic) or FluoroChem (Hadfield, UK). The reactions were monitored by thin-layer chromatography (TLC) on silica gel plates (60 F254, Merck, Prague, Czech Republic), and the spots were visualized by ultraviolet light (254 nm). Purification of crude products was carried out using a PuriFlash Gen5 column, 5.250 (Interchim, Montluçon, France) (silica gel 100, 60 Å, 230–400-mesh ASTM, Sigma-Aldrich, Prague, Czech Republic). NMR spectra were recorded in deuterated chloroform (CDCl3) and deuterated dimethyl sulfoxide (DMSO-d6) on a Bruker Avance NEO 500 MHz spectrometer (499.87 MHz for 1H-NMR and 125.71 MHz for 13C-NMR; Vienna, Austria). Chemical shifts are reported in parts per millions (ppm), and spin multiplicities are given as broad singlet (bs), doublet (d), doublet of doublet (dd), triplet (t), doublet of triplet (dt), quartet (q), pentet (p), or multiplet (m). Coupling constants (J) are reported in Hz. Recorded NMR spectra are available in the Supplementary Materials. Melting points were measured using an automated melting point recorder M-565 (Büchi, Flawil, Switzerland). The synthesized compounds were analyzed by an LC-MS system consisting of UHLPC Dionex Ultimate 3000 RS coupled with a Q Exactive Plus orbitrap mass spectrometer to obtain high-resolution mass spectra (Thermo Fisher Scientific, Bremen, Germany) (see Supplementary Materials). The samples were dissolved in DMSO/methanol 50/50 (*v*/*v*). Reverse-phase C18 column Kinetex EVO (Phenomenex, Torrance, CA, USA) was used as a stationary phase, and purified water with 0.1% formic acid (mobile phase A) and LC-MS grade acetonitrile with 0.1% formic acid (mobile phase B) were used as the mobile phases. Gradient elution was used to determine purities and mass spectra. Method started with 5% B for 0.3 min, then the gradient rose to 100% B in the third min and was at 100% B for 0.7 min and then went back to 5% B and was equilibrated for 3.5 min. Total run time of the method was 7.5 min. Column was tempered to 27 °C, the flow of the mobile phase was 0.5 mL/min, and the injection volume was 1 µL. Gradient LC analysis with UV detection (254 nm) confirmed a >97% purity. High-resolution mass spectra were collected from the total ion current in the scan range 105–1000 *m/z*, with the resolution set to 140,000.

### 2.2. General Procedure for the Preparation of 9-Bromofluorene hydrochlorides (***3a–o***)

**Scheme 1:**
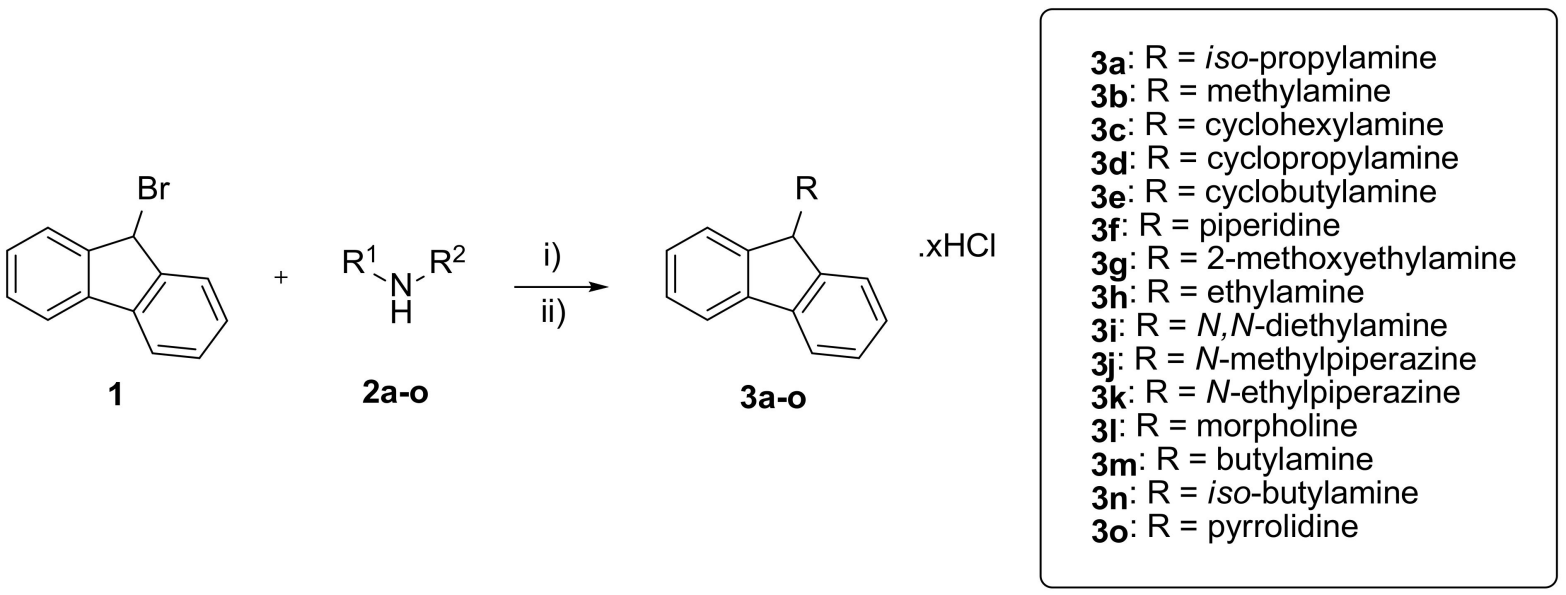
Synthetic procedure for the preparation of substituted fluoren-9-amines **3a**–**o**. Reaction conditions: (i) MeCN, 40 °C, 3 h and (ii) HCl (aq.), MeOH, RT, 1 h.

Appropriate primary or secondary amine (Scheme 1) was dissolved in 10 mL of dry MeCN under argon atmosphere and stirred vigorously at 40 °C for 15 min. 9-Bromofluorene (Scheme 1) was dissolved in 5 mL of dry MeCN and added dropwise to the reaction mixture. The reaction was maintained with stirring at 40 °C for 3 h. After cooling, the solvent was evaporated under reduced pressure, and the crude material was purified on a FlashChrom column (eluent dichloromethane/methanol (DCM/MeOH) with 1% of NH_3_, gradient 95:5 → 90:10) to get the appropriate products as a free base. These were converted into hydrochloride salts by the treatment with 1 mL of concentrated aqueous solution of HCl (35%) in 5 mL of MeOH, starting from 0 °C to room temperature for 1 h. The solvent was removed in vacuo, and the residual water was distilled via azeotropic distillation with absolute EtOH three times. The solid product was washed by ice-cold acetone, resulting in hydrochloride salt as a white solid.

#### 2.2.1. *N*-Propan-2-yl-9*H*-fluoren-9-amine hydrochloride (**3a**)

Yield 43% as a white solid. Melting point (m.p.): decomposed at 238.5 °C. ^1^H-NMR (500 MHz, DMSO-*d*_6_) δ 10.17 (bs, *J* = 5.6 Hz, 2H), 8.12 (dd, *J* = 7.6, 1.0 Hz, 2H), 7.95 (dt, *J* = 7.6, 1.0 Hz, 2H), 7.54 (td, *J* = 7.6, 1.0 Hz, 2H), 7.42 (td, *J* = 7.6, 1.0 Hz, 2H), 5.61–5.57 (m, 1H), 3.27–3.17 (m, 1H), 1.18 (d, *J* = 6.5 Hz, 6H). ^13^C-NMR (126 MHz, dmso) δ 141.41, 138.60, 130.43, 128.33, 127.35, 121.21, 58.25, 48.44, 20.18. HRMS (ESI^+^): [M + H]^+^: calculated for C_16_H_18_N^+^ (*m*/*z*): 224.14392; found: 224.14343. LC-MS purity 99%.

#### 2.2.2. *N*-Methyl-9*H*-fluoren-9-amine hydrochloride (**3b**)

Yield 13% as a white solid. M.p.: decomposed at 205.9 °C. ^1^H-NMR (500 MHz, DMSO-*d*_6_) δ 10.52 (bs, 2H), 8.07 (dd, *J* = 7.6, 1.0 Hz, 2H), 7.94 (dd, *J* = 7.6, 1.0 Hz, 2H), 7.53 (td, *J* = 7.6, 1.0 Hz, 2H), 7.42 (td, *J* = 7.6, 1.0 Hz, 2H), 5.64 (s, 1H), 2.12 (s, 3H). ^13^C-NMR (126 MHz, dmso) δ 141.42, 137.56, 130.25, 128.20, 126.62, 120.94, 59.75, 27.06. HRMS (ESI^+^): [M + H]^+^: calculated for C_14_H_14_N^+^ (*m*/*z*): 196.11262; found: 196.11229. LC-MS purity 100%.

#### 2.2.3. *N*-Cyclohexyl-9*H*-fluoren-9-amine hydrochloride (**3c**)

Yield 43% as a white solid. M.p. 267.3–269.6 °C. ^1^H-NMR (500 MHz, DMSO-*d*_6_) δ 10.17 (bs, 2H), 8.10 (dd, *J* = 7.6, 1.0 Hz, 2H), 7.95 (dd, *J* = 7.6, 1.0 Hz, 2H), 7.54 (td, *J* = 7.6, 1.0 Hz, 2H), 7.42 (td, *J* = 7.6, 1.0 Hz, 2H), 5.60 (s, 1H), 2.84 (t, *J* = 11.5 Hz, 1H), 1.81 – 1.71 (m, 2H), 1.68–1.59 (m, 2H), 1.50 (m, *J* = 15.0, 5.3, 4.4 Hz, 3H), 1.13–1.00 (m, 3H). ^13^C-NMR (126 MHz, dmso) δ 141.09, 138.37, 130.15, 128.09, 127.00, 120.94, 57.68, 54.59, 29.72, 24.68, 23.99. HRMS (ESI^+^): [M + H]^+^: calculated for C_19_H_22_N^+^ (*m*/*z*): 264.17522; found: 264.17465. LC-MS purity 99%.

#### 2.2.4. *N*-Cyclopropyl-9*H*-fluoren-9-amine hydrochloride (**3d**)

Yield 57% as a white solid. M.p.: decomposed at 233.1 °C. ^1^H-NMR (500 MHz, DMSO-*d*_6_) δ 10.63 (bs, 2H), 8.13 (dd, *J* = 7.6, 1.0 Hz, 2H), 7.94 (dd, *J* = 7.6, 1.0 Hz, 2H), 7.53 (td, *J* = 7.6, 1.0 Hz, 2H), 7.41 (td, *J* = 7.6, 1.0 Hz, 2H), 5.63 (s, 1H), 2.25–2.15 (m, 1H), 0.85–0.76 (m, 2H), 0.55–0.46 (m, 2H). ^13^C-NMR (126 MHz, dmso) δ 141.38, 138.07, 130.14, 127.95, 127.08, 120.82, 60.17, 26.24, 3.50. HRMS (ESI^+^): [M + H]^+^: calculated for C_16_H_16_N^+^ (*m*/*z*): 222.12827; found: 222.12776. LC-MS purity 99%.

#### 2.2.5. *N*-Cyclobutyl-9*H*-fluoren-9-amine hydrochloride (**3e**)

Yield 51% as a white solid. M.p.: decomposed at 271.6 °C. ^1^H-NMR (500 MHz, DMSO-*d*_6_) δ 10.77 (bs, 2H), 8.10 (dd, *J* = 7.6, 1.0 Hz, 2H), 7.92 (dd, *J* = 7.6, 1.0 Hz, 2H), 7.52 (td, *J* = 7.6, 1.0 Hz, 2H), 7.40 (td, *J* = 7.6, 1.0 Hz, 2H), 5.57 (s, 1H), 3.12 (p, *J* = 9.1, 8.6 Hz, 1H), 2.22–2.10 (m, 2H), 1.62–1.41 (m, 4H). ^13^C-NMR (126 MHz, dmso) δ 141.23, 137.87, 130.16, 127.97, 126.96, 120.84, 58.50, 47.72, 27.67, 15.64. HRMS (ESI^+^): [M + H]^+^: calculated for C_17_H_18_N^+^ (*m*/*z*): 236.14392; found: 236.14333. LC-MS purity 99%.

#### 2.2.6. 1-(9*H*-fluoren-9-yl)piperidine hydrochloride (**3f**)

Yield 45% as a white solid. M.p.: decomposed at 251.4 °C. ^1^H-NMR (500 MHz, DMSO-*d*_6_) δ 11.79 (bs, 1H), 8.24 (dd, *J* = 7.6, 1.0 Hz, 2H), 7.94 (dd, *J* = 7.6, 1.0 Hz, 2H), 7.55 (td, *J* = 7.6, 1.0 Hz, 2H), 7.42 (td, *J* = 7.6, 1.0 Hz, 2H), 5.72 (s, 1H), 3.23 (d, *J* = 11.7 Hz, 2H), 2.79 (q, *J* = 11.7 Hz, 2H), 2.07 (q, *J* = 12.6, 11.7 Hz, 2H), 1.67 (d, *J* = 12.6 Hz, 3H), 1.34–1.20 (m, 1H). ^13^C-NMR (126 MHz, dmso) δ 141.83, 135.94, 130.66, 128.29, 128.17, 120.91, 67.23, 49.46, 22.29, 21.67. HRMS (ESI^+^): [M + H]^+^: calculated for C_18_H_20_N^+^ (*m*/*z*): 250.15957; found: 250.15875. LC-MS purity 98%.

#### 2.2.7. *N*-(2-methoxyethyl)-9*H*-fluoren-9-amine hydrochloride (**3g**)

Yield 11% as a white solid. M.p.: 183.4–185.1 °C. ^1^H-NMR (500 MHz, DMSO-*d*_6_) δ 10.60 (bs, 2H), 8.15 (dd, *J* = 7.6, 1.0 Hz, 2H), 7.93 (dd, *J* = 7.6, 1.0 Hz, 2H), 7.53 (td, *J* = 7.6, 1.0 Hz, 2H), 7.42 (td, *J* = 7.6, 1.0 Hz, 2H), 5.59 (s, 1H), 3.47 (t, *J* = 5.2 Hz, 2H), 3.19 (s, 3H), 2.47–2.40 (m, 2H). ^13^C-NMR (126 MHz, dmso) δ 141.42, 137.56, 130.27, 128.26, 126.69, 120.93, 67.03, 59.32, 58.16, 40.87. HRMS (ESI^+^): [M + H]^+^: calculated for C_16_H_18_NO^+^ (*m*/*z*): 240.13884; found: 240.13831. LC-MS purity 99%.

#### 2.2.8. *N*-Ethyl-9*H*-fluoren-9-amine hydrochloride (**3h**)

Yield 36% as a white solid. M.p.: decomposed at 264.8 °C. ^1^H-NMR (500 MHz, DMSO-*d*_6_) δ 10.46 (bs, *J* = 9.3 Hz, 2H), 8.12 (dd, *J* = 7.6, 1.0 Hz, 2H), 7.94 (dd, *J* = 7.6, 1.0 Hz, 2H), 7.53 (td, *J* = 7.6, 1.0 Hz, 2H), 7.42 (td, *J* = 7.6, 1.0 Hz, 2H), 5.61 (d, *J* = 3.5 Hz, 1H), 2.51–2.48 (m, 2H), 1.14 (t, *J* = 7.2 Hz, 3H). ^13^C-NMR (126 MHz, dmso) δ 141.29, 137.89, 130.19, 128.18, 126.72, 120.91, 59.17, 37.35, 11.53. HRMS (ESI^+^): [M + H]^+^: calculated for C_15_H_16_N^+^ (*m*/*z*): 210.12827; found: 210.12784. LC-MS purity 99%.

#### 2.2.9. *N*,*N*-Diethyl-9*H*-fluoren-9-amine hydrochloride (**3i**)

Yield 7% as a white solid. M.p.: 166.2–167.9 °C. ^1^H-NMR (500 MHz, DMSO-*d*_6_) δ 11.66 (bs, 1H), 8.15 (dd, *J* = 7.6, 1.0 Hz, 2H), 7.98 (dd, *J* = 7.6, 1.0 Hz, 2H), 7.57 (td, *J* = 7.6, 1.0 Hz, 2H), 7.44 (td, *J* = 7.6, 1.0 Hz, 2H), 5.85 (s, 1H), 3.12–3.02 (m, 1H), 3.02–2.94 (m, 1H), 1.30 (t, *J* = 7.2 Hz, 6H). ^13^C-NMR (126 MHz, dmso) δ 141.62, 136.45, 130.54, 128.37, 127.49, 121.07, 63.15, 46.29, 10.32. HRMS (ESI^+^): [M + H]^+^: calculated for C_17_H_20_N^+^ (*m*/*z*): 238.15957; found: 238.15903. LC-MS purity 99%.

#### 2.2.10. 1-(9*H*-fluorene-9-yl)-4-methylpiperazine dihydrochloride (**3j**)

Yield 7% as a white solid. M.p.: decomposed at 206.4 °C. ^1^H-NMR (500 MHz, DMSO-*d*_6_) δ 10.80 (bs, 1H), 7.85 (dd, *J* = 7.6, 1.0 Hz, 2H), 7.59 (dd, *J* = 7.6, 1.0 Hz, 2H), 7.42 (td, *J* = 7.6, 1.0 Hz, 2H), 7.34 (td, *J* = 7.6, 1.0 Hz, 2H), 5.03 (s, 1H), 3.31–2.71 (m, 8H), 2.68 (s, 3H). ^13^C-NMR (126 MHz, dmso) δ 143.00, 140.64, 128.64, 127.51, 125.88, 120.36, 68.65, 53.41, 45.50, 42.34. HRMS (ESI^+^): [M + H]^+^: calculated for C_18_H_21_N_2_^+^ (*m*/*z*): 265.17047; found: 265.16974. LC-MS purity 99%.

#### 2.2.11. 1-(9*H*-fluorene-9-yl)-4-ethylpiperazine dihydrochloride (**3k**)

Yield 25% as a white solid. M.p.: decomposed at 233.6 °C. ^1^H-NMR (500 MHz, DMSO-*d*_6_) δ 10.75 (bs, 1H), 7.85 (dd, *J* = 7.6, 1.0 Hz, 2H), 7.59 (dd, *J* = 7.6, 1.0 Hz, 2H), 7.42 (td, *J* = 7.6, 1.0 Hz, 2H), 7.33 (td, *J* = 7.6, 1.0 Hz, 2H), 5.03 (s, 1H), 3.02 (q, *J* = 7.3 Hz, 2H), 2.99–2.61 (m, 8H), 1.19 (t, *J* = 7.3 Hz, 3H). ^13^C-NMR (126 MHz, dmso) δ 143.05, 140.63, 128.62, 127.49, 125.90, 120.34, 68.67, 51.28, 50.79, 45.45, 9.01. HRMS (ESI^+^): [M + H]^+^: calculated for C_19_H_24_N_2_^+^ (m/z): 279.18612; found: 279.18555. LC-MS purity 99%.

#### 2.2.12. 4-(9*H*-fluorene-9-yl)morpholine hydrochloride (**3l**)

Yield 50% as a white solid. M.p.: decomposed at 239.8 °C. ^1^H-NMR (500 MHz, DMSO-*d*_6_) δ 12.66 (bs, 1H), 8.21 (dd, *J* = 7.6, 1.0 Hz, 2H), 7.96 (dd, *J* = 7.6, 1.0 Hz, 2H), 7.58 (td, *J* = 7.6, 1.0 Hz, 2H), 7.45 (td, *J* = 7.6, 1.0 Hz, 2H), 5.81 (s, 1H), 4.18–3.93 (m, 2H), 3.94–3.75 (m, 2H), 3.31–3.09 (m, 2H), 3.09–2.87 (m, 2H). ^13^C-NMR (126 MHz, DMSO) δ 142.19, 131.09, 128.60, 128.46, 121.30, 67.38, 63.41, 48.58. HRMS (ESI^+^): [M + H]^+^: calculated for C_17_H_18_NO^+^ (*m*/*z*): 252.13884; found: 252.13837. LC-MS purity 99%.

#### 2.2.13. *N*-Butyl-9*H*-fluoren-9-amine hydrochloride (**3m**)

Yield 12% as a white solid. M.p.: decomposed at 201.3 °C. ^1^H-NMR (500 MHz, DMSO-*d*_6_) δ 10.13 (bs, 2H), 8.10 (dd, *J* = 7.6, 1.0 Hz, 2H), 7.93 (dd, *J* = 7.6, 1.0 Hz, 2H), 7.52 (td, *J* = 7.6, 1.0 Hz, 2H), 7.42 (td, *J* = 7.6, 1.0 Hz, 2H), 5.54 (s, 1H), 2.33 (t, 2H), 1.58–1.47 (m, 2H), 1.25–1.14 (m, 2H), 0.74 (t, *J* = 7.3 Hz, 3H). ^13^C-NMR (126 MHz, DMSO) δ 141.52, 130.26, 128.40, 126.78, 121.12, 59.98, 41.80, 28.70, 19.70, 13.86. HRMS (ESI^+^): [M + H]^+^: calculated for C_17_H_20_N^+^ (*m*/*z*): 238.15957; found: 238.15912. LC-MS purity 97%.

#### 2.2.14. *N*-(2-methylpropyl)-9*H*-fluoren-9-amine hydrochloride (**3n**)

Yield 34% as a white solid. M.p.: 254.8–256.1 °C. ^1^H-NMR (500 MHz, DMSO-*d*_6_) δ 10.52 (bs, 2H), 8.19 (dd, *J* = 7.6, 1.0 Hz, 2H), 7.94 (dd, *J* = 7.6, 1.0 Hz, 2H), 7.54 (td, *J* = 7.6, 1.0 Hz, 2H), 7.44 (td, *J* = 7.6, 1.0 Hz, 2H), 5.62 (s, 1H), 2.13–2.03 (m, 2H), 1.98–1.85 (m, 1H), 0.82 (d, *J* = 6.7 Hz, 6H). ^13^C-NMR (126 MHz, DMSO) δ 141.72, 137.87, 130.54, 128.56, 126.89, 121.22, 59.76, 48.58, 26.03, 20.52. HRMS (ESI^+^): [M + H]^+^: calculated for C_17_H_20_N^+^ (*m*/*z*): 238.15957; found: 238.15903. LC-MS purity 97%.

#### 2.2.15. 1-(9*H*-fluoren-9-yl)pyrrolidine hydrochloride (**3o**)

Yield 30% as a white solid. M.p.: decomposed at 239.3 °C. ^1^H-NMR (500 MHz, DMSO-*d*_6_) δ 12.31 (bs, 1H), 8.14 (dd, *J* = 7.6, 1.0 Hz, 2H), 7.96 (dd, *J* = 7.6, 1.0 Hz, 2H), 7.56 (td, *J* = 7.6, 1.0 Hz, 2H), 7.43 (td, *J* = 7.6, 1.0 Hz, 2H), 5.84 (s, 1H), 3.49–3.27 (m, 2H; signal overlapped with residual DMSO), 3.13–2.92 (m, 2H), 1.97–1.72 (m, 4H). ^13^C-NMR (126 MHz, DMSO) δ 141.85, 130.84, 128.57, 127.78, 121.22, 64.49, 50.55, 23.96. HRMS (ESI^+^): [M + H]^+^: calculated for C_17_H_18_N^+^ (*m*/*z*): 236.14392; detected: 236.14328. LC-MS purity 99%.

### 2.3. In Vitro Anti-ChE Assay

The human AChE/human BChE (*h*AChE/*h*BChE) inhibitory activity of the tested derivatives was determined using Ellman’s method [46,47,48] and is expressed as IC_50_, i.e., a concentration that reduces the cholinesterase activity by 50%. The human recombinant BChE and AChE were prepared at the Department of Toxicology and Military Pharmacy (Faculty of Military Health Sciences, Hradec Kralove, Czech Republic). 5,5′-dithiobis(2-nitrobenzoic acid) (Ellman’s reagent, DTNB), phosphate buffer (PB, pH 7.4), acetylthiocholine (ATC), and butyrylthiocholine (BTC) were purchased from Sigma-Aldrich, Prague, Czech Republic. For measuring purposes, polystyrene Nunc 96-well microplates with a flat bottom shape (Thermo Fisher Scientific, Waltham, MA, USA) were utilized. All the assays were carried out in 0.1-M KH_2_PO_4_/K_2_HPO_4_ buffer, pH 7.4. Enzyme solutions were prepared at 2.0 units/mL in 2-mL aliquots. The assay medium (100 µL) consisted of 40 µL of 0.1-M phosphate buffer (pH 7.4), 20 µL of 0.01-M DTNB, 10 µL of the enzyme, and 20 µL of 0.01-M substrate (ATC/BTC iodide solution). Inhibitor solutions in the concentration range 10^−3^–10^−11^ M were prepared, and IC_50_ values were calculated. Tested compounds were preincubated for 5 min. The reaction was started by the immediate addition of 20 µL of the substrate. The activity was determined by measuring the increase in absorbance at 412 for *h*AChE/*h*BChE at 37 °C at 2 min intervals using a multimode microplate reader Synergy 2 (BioTek Instruments, Inc., Winooski, VT, USA). Each concentration was assayed in triplicate. Software GraphPad Prism 5 (San Diego, CA, USA) was used for the statistical data evaluation.

### 2.4. Kinetic Study of hBChE Inhibition

The kinetic study of *h*BChE was performed by using the above-mentioned modified Ellman’s method. The values of *V*_max_ and *K*_m_ of the Michaelis-Menten kinetics, as well as the value of *K*_i_, were calculated by nonlinear regression from the substrate velocity curves. Linear regression was used for calculation of the Cornish-Bowden plots. All calculations were performed using GraphPad Prism software version 6.07 for Windows (San Diego, CA, USA).

### 2.5. Antagonist Activity Towards the NMDA receptor

#### 2.5.1. HEK293 Cell Culture and Transfection

Human embryonic kidney 293 (HEK293) cells were cultured in Opti-MEM I containing 5% fetal bovine serum (FBS; both from Thermo Fisher Scientific) [49]. The cells grown in a 24-well plate were put in Opti-MEM I media containing a mixture of 0.9 µL of *MATra*-A Reagent (*IBA*) and 900 ng of DNA vectors carrying the human versions of the GluN1-1a (GluN1), GluN2A, or GluN2B subunits and green fluorescent protein (GFP; all vectors were added in equal ratio) [39,50,51]. The cells were placed on a strong magnet plate for 30 min and then trypsinized; resuspended in Opti-MEM I containing 1% FBS, 20-mM MgCl_2_, and 3-mM kynurenic acid (to inhibit the NMDAR-induced excitotoxicity); and plated on poly-l-lysine-coated glass coverslips. The electrophysiological experiments were executed 24–48 h after transfection [52].

#### 2.5.2. Electrophysiology

Whole-cell patch-clamp recordings were conducted at room temperature on the GFP-positive HEK293 cells using an Axopatch 200B amplifier (Molecular Devices, San Jose, CA, USA); the series resistance compensation was >80% for all cells [39]. The recorded currents were filtered at 2 kHz with an eight-pole low-pass Bessel filter and digitized at 5 kHz with Digidata 1322A and pClamp 10 software (Molecular Devices). The WAS02 application system, which can reach the solution exchange time around the recorded cell of 10–20 ms, was used to apply the extracellular solution (ECS) [53]. The ECS contained (in mM): 160 NaCl, 2.5 KCl, 10 HEPES, 10 glucose, 0.2 EDTA, and 0.7 CaCl_2_ (pH adjusted to 7.3 with NaOH) and was supplemented with the saturating concentrations of co-agonists glycine (50 µM) and agonist glutamate (1 mM; both from Merck). The stock solutions of **3a*–*o** (10 mM) were prepared freshly before each experiment in dimethylsulfoxide (DMSO; Merck), and the background concentration of DMSO was kept equal in each ECS. The borosilicate glass pipettes with a tip resistance ~4-7 MΩ were made using a P-1000 micropipette puller (Sutter Instrument Co., Novato, CA, USA) and were filled with an intracellular solution (in mM: 125 gluconic acid, 15 CsCl, 5 EGTA, 10 HEPES, 3 MgCl_2_, 0.5 CaCl_2_, and 2 ATP-Mg salt (pH adjusted to 7.2 with CsOH). Data were analyzed using SigmaPlot 14.0 (Systat Software, Inc., Chicago, IL, USA), and the dose-response curves were built using Equation (1): I = 1/(1 + ([compound]/IC_50_)^h^), where IC_50_ is the concentration of the compound that produces a 50% inhibition of the glutamate-evoked current, [compound] is the concentration of the studied compound, and h is the apparent Hill coefficient [54].

### 2.6. In Vitro Cell Viability Assessment

Standard MTT assay (3-(4,5-dimethylthiazol-2-yl)-2,5-diphenyltetrazolium bromide; Sigma Aldrich, Prague, Czech Republic) was used according to the manufacturer’s protocols on the CHO-K1 (Chinese hamster ovary, ECACC, Salisbury, UK) and hCMEC/D3 (Sigma Aldrich, St. Louis, MO, USA). The cells were cultured according to recommended conditions and seeded in a density of 8000 cells per well, as described previously [55]. Briefly, the tested compounds were dissolved in dimethyl sulfoxide (DMSO; Sigma Aldrich, St. Louis, MO, USA) and subsequently diluted in the nutrient mixture F-12 ham growth medium (Sigma Aldrich, St. Louis, MO, USA), supplemented with 10% fetal bovine serum and 1% penicillin-streptomycin (both Sigma Aldrich, St. Louis, MO, USA), so that the final concentration of DMSO did not exceed 0.5% (*v*/*v*). CHO-K1 cells were exposed to serially diluted tested compounds for 24 h. Then, the medium was replaced by a fresh medium containing 0.5 mg/mL of MTT, and the cells were allowed to produce formazan for another approximately 3 h under surveillance. Thereafter, the medium with MTT was removed, and crystals of formazan were dissolved in DMSO (100 µL/well; PENTA s.r.o., Prague, Czech Republic). Cell viability was assessed spectrophotometrically by the amount of formazan produced. The absorbance was measured at 570 nm with a 650-nm reference wavelength on Synergy HT (BioTek, Winooski, VT, USA). The IC_50_ value (half-maximal inhibitory concentration) was calculated from the control-subtracted triplicates using nonlinear regression (four parameters) by GraphPad Prism 7.03 software (GraphPad Software Inc., San Diego, CA, USA) for the CHO-K1 cell line, and for hCMEC/D3 cells, the cell viability was expressed as the % relative to the untreated control. Final IC_50_ and SEM (standard error of the mean) values were obtained as a mean of three independent measurements.

### 2.7. Determination of In Vitro BBB Permeation

The D3 assay evaluates the ability of compounds to diffuse from the donor compartment through the D3/hCMEC cell membrane into the acceptor compartment. The D3 cells were seeded on a PET membrane (area 1.12 cm^2^) with 3-µm pores of the 12-well plates with 12-mm inserts. The tested compounds were dissolved in DMSO and then diluted with Opti-MEM to reach final a concentration of 30 µM for **3m** and 50 μM for others; the concentration of DMSO did not exceed 0.5% (*v*/*v*). The donor solution (750 µL) was added to the donor compartment (insert), and the same volume of Opti-MEM was added into the acceptor. The concentration of the drug in both compartments was measured by UV-VIS spectrophotometry in 5, 15, 30, and 60 min of incubation in triplicates. The apparent permeability coefficient (*P_app_*) was calculated from the concentration ratios. Tightness of the D3 monolayer was conducted by the fluorescein isothiocyanate (FITC) test after each experiment. The accepted values of FITC (fluorescein isothiocyanate) in 0.4 mg/mL in the acceptor compartment after 30 min of incubation must not exceed 1.5% of the initial donor concentration.

(1) $$Papp=\;\left(\frac{\mathrm{dC}}{\mathrm{dt}}\right)\;\times \;\frac{V_{r}}{\left(A\;\times {\;C}_{0}\right)}$$

A: area of the well/cell monolayer,
dC/dt: amount in the receiver compartment in given time,
V_r_: volume of the receiver compartment, and
C_0_: the initial concentration of tested compounds.

### 2.8. Molecular Modeling Studies

The model of *h*BChE (Protein Data Bank (PDB) ID: 4BDS, resolution: 2.10 Å) was downloaded from the RCSB Protein Data Bank (https://rcsb.org) and prepared for flexible molecular docking by MGL Tools [56,57]. The preparation of these receptors involved removal of the inhibitor, water molecules and nonbonded co-crystalized compounds, and the addition of polar hydrogens. Default Gasteiger charges were assigned to all atoms. Flexible parts of the enzymes were set based on previous experiences [58,59]. The rotatable bonds in the flexible residues were detected automatically by AutoDock Tools 1.5.4. The flexible receptor parts contained 39 residues. The studied ligands were firstly drawn in HyperChem 8.0, then manually protonated, as suggested by MarvinSketch 18.24.0. software (http://www.chemaxon.com), geometrically optimized by software Avogadro, and stored as pdb files. The structures of the ligands were processed for docking by the AutoDock Tools 1.5.4 program. Molecular docking was carried out by the software AutoDock Vina 1.1.2 utilizing computer resources of the Czech National Grid Infrastructure MetaCentrum. The search algorithm of AutoDock Vina efficiently combines a Markov chain Monte Carlo-like method for the global search and a Broyden-Fletcher-Goldfarb-Shano gradient approach for the local search [60]. It is a type of memetic algorithm based on interleaving stochastic and deterministic calculations [61]. Each docking task was repeated 20 times with the exhaustiveness parameter set to 8, employing 8 CPUs in parallel multithreading. From the obtained results, the solutions reaching the minimum docking score were taken as the top-scoring modes. The graphical representations of the docked poses were shown in PyMOL (The PyMOL Molecular Graphics System, Version 2.0.6. Schrödinger, LLC, New York, NY, USA). 2D diagrams were generated using Maestro 12.3 (Schrödinger Release, Schrödinger, LLC, New York, NY, USA).

### 2.9. In Silico Pharmacokinetics and Drug-Likeness Prediction

SwissADME, web tool was used to predict gastrointestinal absorption, BBB permeation and bioavailability [62]. Physicochemical properties (p*K*_a_, Clog*P*, HBA, HBD, and TPSA) were predicted by MarvinSketch 20.4.0, ChemAxon Ltd. (Budapest, Hungary; see Table S1, Supplementary Materials). BBB scores were calculated by the BBB calculator available at the ACS website [63].

## 3. Results and Discussion

### 3.1. In Silico Prediction of the CNS and Oral Availability

**Figure 2:**
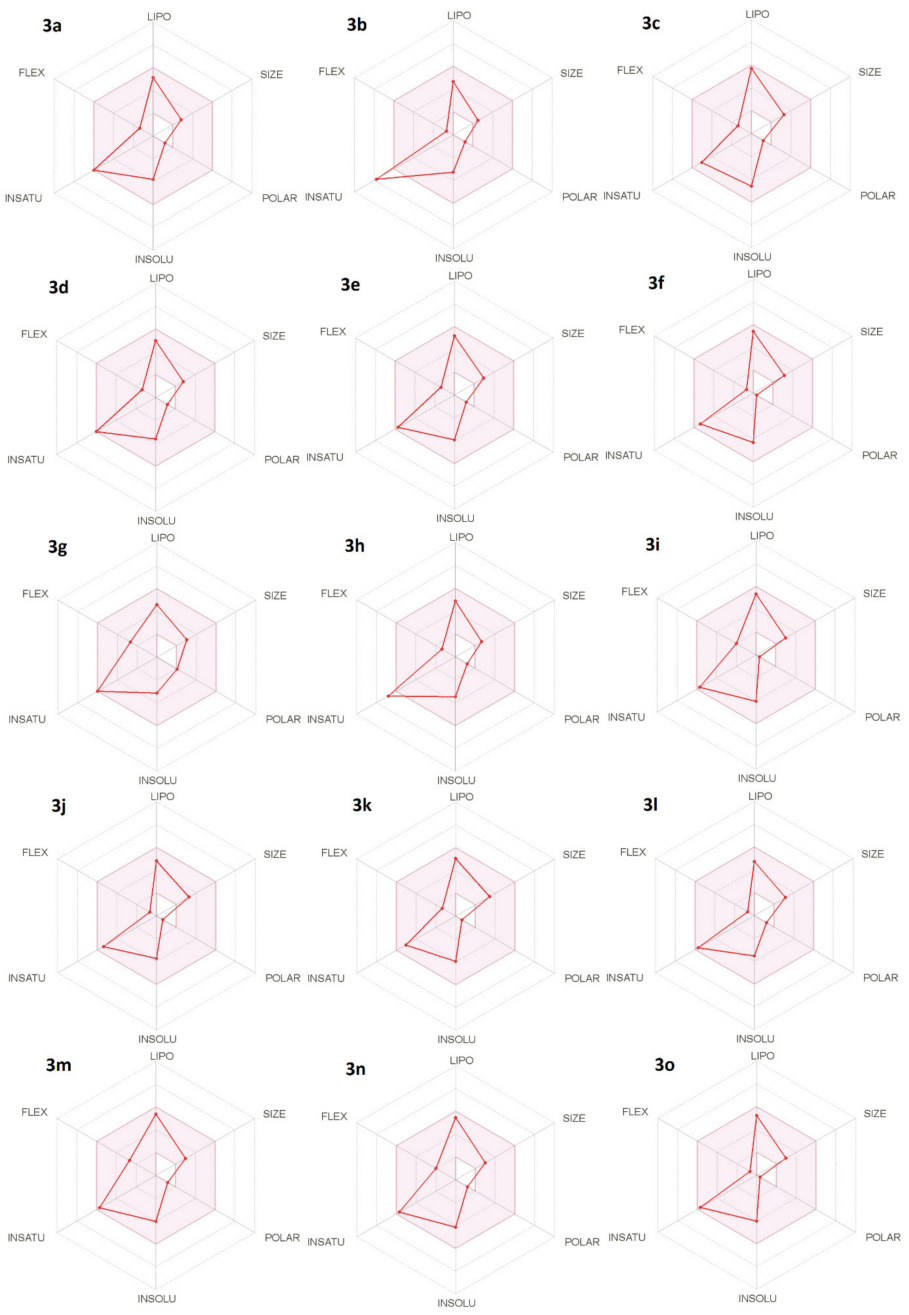
The bioavailability radar chart of the designed fluorene-9-amines **3a–o**.

**Figure 3:**
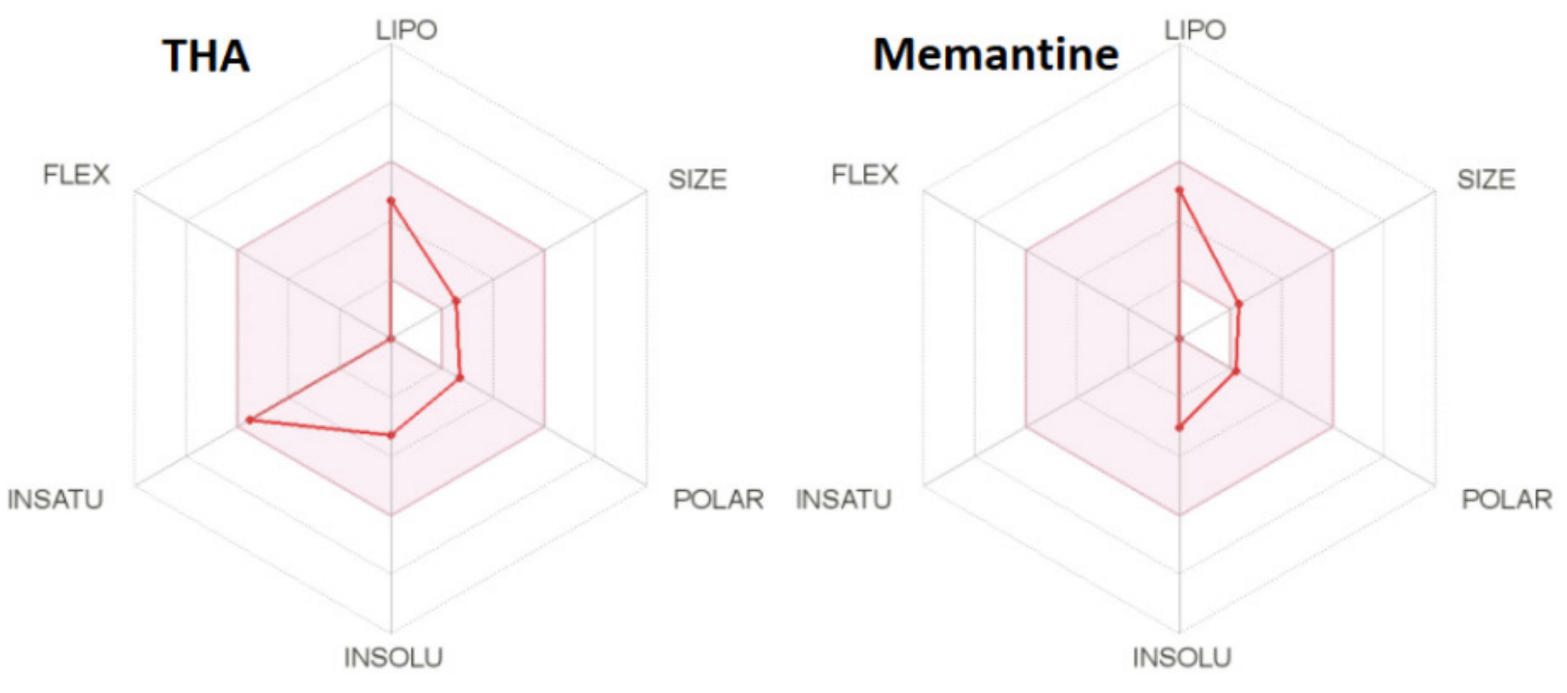
The bioavailability radar chart for the standards THA and memantine (please see detailed description in the text).

All the newly designed compounds were initially screened in silico to predict their peroral and CNS availability. Indeed, both of these features are essential in the drug discovery process of small molecules as potential anti-AD therapeutics and should be estimated before synthesis. To this end, the in silico pharmacokinetics; drug-likeness; and ADME (absorption, distribution, metabolism, and excretion) prediction was applied to compounds **3a**–**o** using a web-based tool SwissADME [62,64,65]. THA and memantine were used as references with known CNS statuses and pharmacokinetic profiles [42,66,67]. Data are presented in Figure 2 and Figure 3 and Table 1 and Table S1.

The pink middle area (Figure 2 and Figure 3) represents the optimum range for bioavailability. It is a sum of the lipophilicity (LIPO), size, polarity (POLAR), solubility (INSOLU), saturation (INSATU), and flexibility (FLEX). The red lines show the predicted state for the designed compounds [62,64,65]. The calculated parameters for all the compounds, except for **3b** and **3h**, are situated inside the pink area, suggesting their high oral bioavailability [69]. A positive correlation for the bioavailability was also supported by the BOILED-Egg method [70], proposing high gastrointestinal adsorption and penetration through the BBB [70]. The BBB score, a new predictive model for CNS availability, was also calculated for all the derivatives fitting the range between 5 and 6, indicating a high probability to permeate through the BBB (estimated physicochemical parameters are shown in Table S1, Supplementary Materials) [63]. Besides, all compounds also fulfill the drug-likeness rules of pharmacological models defined by pharmaceutical companies like Veber’s (GSK) [71], Lipinski’s (Pfizer) [72], Egan’s (Pharmacopeia) [73], Ghose’s (Amgen) [74], and Muegge’s (Bayer) [75] (Table S2, Supplementary Materials). In summary, derivatives **3a**–**o** unambiguously show high predictive peroral bioavailability, BBB permeation, and acceptable pharmacokinetic profiles, making the compounds prospective candidates for the treatment of CNS disorders, including AD.

### 3.2. Synthesis

Fluoren-9-amines were prepared in a one-step procedure from commercially available 9-bromofluorene (**1**) with an excess of various primary or secondary amines (**2a**–**o**) in dry acetonitrile (Scheme 1). This reaction afforded the formation of fluoren-9-amines (**3a**–**o**) in high yields (80–95%). The free bases were then converted to the respective hydrochloride salts. The final products were characterized by ^1^H, ^13^C-NMR spectra, melting points, and HRMS analysis. LC analysis confirmed the purity for all the derivatives > 97% (Supplementary Materials).

### 3.3. Evaluation of Cholinesterase Inhibitory Activity

Fluoren-9-amines were tested for their inhibitory potential against human AChE (*h*AChE) and human BChE (*h*BChE) enzymes using the modified spectrophotometric method of Ellman et al. [46,48,76]. THA was used as a reference compound. The IC_50_ values of all tested compounds and their selectivity indexes (SI) for *h*BChE are summarized in Table 2. All tested derivatives showed a preferential inhibition of *h*BChE (usually in the single-digit micromolar range), whereas only compounds **3c** and **3m** showed weak inhibitory activity for *h*AChE at 13.98 and 49.91 µM, respectively. The *h*BChE inhibition ranged between 0.47–54.65 µM. According to the *h*BChE inhibition potency, the top-ranked derivative from all the derivatives under the study was **3c** (IC_50_ = 0.47 µM), being 20 times less active compared to THA. However, the relatively unique selectivity to BChE is of immense interest. Indeed, the selectivity profile for the ChE drugs potentially useful in AD treatment is extensively discussed [77]. The currently used ChE inhibitors are AChE-selective (donepezil) or possess more or less nonselective patterns of inhibition (rivastigmine and galantamine) [25]. It was proven that the levels of BChE in the brain of AD patients are elevated with age, while the levels of AChE are suppressed. This finding underlines the importance of selective-BChE inhibitors in the treatment of the later stages of AD [77,78].

### 3.4. Kinetic Study of hBChE Inhibition

**Figure 4:**
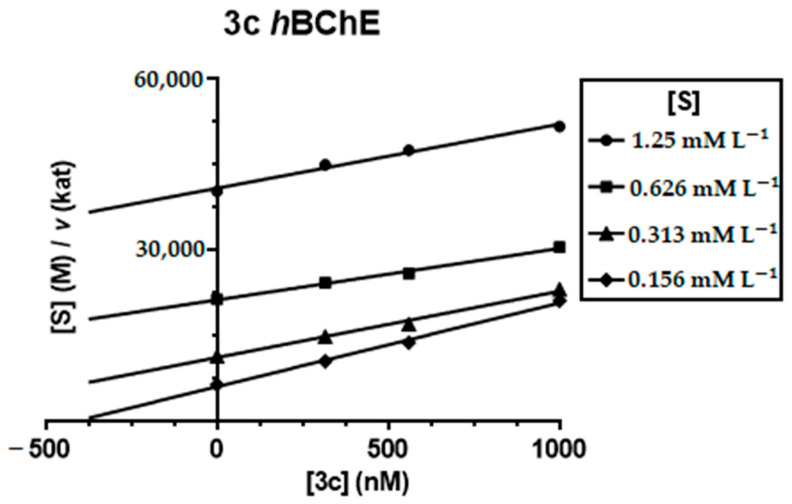
Steady-state inhibition of the *h*BChE substrate (human butyrylthiocholine) hydrolysis by compound **3c** at different concentrations. Cornish−Bowden plots of (S)/*v* against the (I) of the initial velocity at increasing substrate concentrations (2.5–20.0 mM) are presented. Lines were derived from the linear regression of the data points. Each point is the average of three determinations.

To determine the inhibition pattern of **3c**, a kinetic study was performed to elucidate the interaction mechanism of the compound with *h*BChE. The kinetics of the inhibition were revealed from velocity curves measured at several concentrations of **3c** and the *h*BChE substrate butyrylthiocholine. The type of enzyme inhibition and appropriate affinity parameter (*K*_i_) were determined using nonlinear regression analysis. The results for each type of model of inhibition (competitive, noncompetitive, uncompetitive, and mixed) were compared by the sum-of-squares *F-*test. A statistical analysis showed a competitive type of inhibition for *h*BChE (*p* < 0.05) that was in line with the Cornish–Bowden plot used for visualization of the obtained data (Figure 4).

The parallel lines indicate a competitive inhibition of *h*BChE by **3c**, which means a reversible binding mode to the active site of the enzyme. With the increasing concentration of the inhibitor, the apparent *V*_max_ remained unchanged and *K*_m_ increased. A *K*_i_ value of 173 ± 25 nM was determined for **3c** on *h*BChE.

### 3.5. Evaluation of Antagonist Activity towards the NMDA Receptor

**Figure 5:**
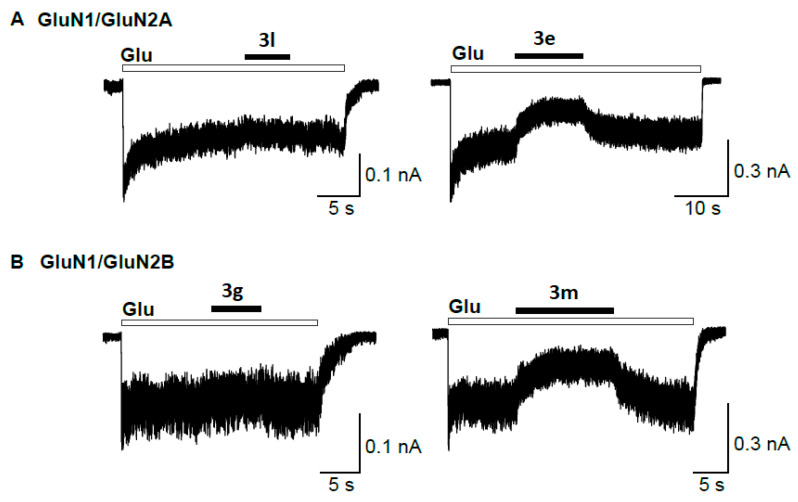
The examples of the most (**3e** and **3m**) and least (**3l** and **3g**) potent compounds from all tested fluoren-9-amines at the NMDARs. Representative whole-cell currents induced by 1 mM of glutamate (Glu) and 50 μM of glycine at the negative (−60 mV) membrane potential with (**A**) the least potent compounds: **3l** measured at GluN1/GluN2A and **3g** at GluN1/GluN2B NMDARs and (**B**) the most potent compounds: **3e** measured at GluN1/GluN2A and **3m** at GluN1/GluN2B NMDA receptor (see Table 3).

Further, we aimed to examine the effects of all synthesized compounds (**3a**–**o**) at both the GluN1/GluN2A and GluN1/GluN2B receptors. First, we held the transfected cells at a membrane potential of −60 mV, and we assessed the relative inhibitory effect of each compound at a 10-µM concentration upon its application with the (co-)agonists (Figure 4). These experiments showed that all studied compounds exhibited an inhibitory effect ranging from ~7% to ~52% at both studied NMDAR combinations (Table 3). Interestingly, we revealed that **3e** was the most potent compound at the GluN1/GluN2A receptors, while **3m** was at the GluN1/GluN2B receptors. Subsequently, we generated concentration-response curves for **3e** and **3m** (1–300 µM) for both the GluN1/GluN2A and GluN1/GluN2B receptors at the negative (−60 mV) and positive (40 mV) membrane potentials (Figure 5). Our analysis showed that both compounds exhibited a more profound inhibitory effect at the negative membrane potential (IC_50_ values ranged from ~9 µM to ~15 µM), while they were much less potent at the positive membrane potential (IC_50_ values ranged from ~83 µM to ~221 µM); the results are summarized in Table 4. Together, our data show that both **3e** and **3m** act as potent voltage-dependent inhibitors of the NMDARs, suggesting that they act as open-channel blockers of NMDARs. Interestingly, the IC_50_ values for both **3e** and **3m** at the GluN1/GluN2A and GluN1/GluN2B receptors were in a similar range to those previously obtained with THA (Table 4) [39]. However, both **3e** and **3m** were less potent than memantine under the studied conditions (Table 4).

### 3.6. In Vitro Cell Viability Assessment

The in vitro cytotoxicity evaluation on mammalian cells served as a preliminary toxicity indicator for the newly developed compounds (Table 5). In our previous work, we observed a correlation between the calculated log*P* (Clog*P*) values (MarvinSketch software, v. 18.24.0; Table 5) and the toxicity; however, our results within the presented study showed no clear relationship [80]. For instance, one of the most lipophilic compounds (**3f**) yielded as the least toxic, whereas other more hydrophilic fluoren-9-amines were regarded as low-toxic (e.g., **3g** as the most hydrophilic compound) agents as well. The least cytotoxic compound can be classified as **3f** with an attached piperidine moiety. At the pole separation, the most cytotoxic agents were **3c**, **3m**, and **3e**. Importantly, some of the new derivatives were less cytotoxic than THA.

### 3.7. In Vitro BBB Permeation

The top-ranked BChE inhibitors and NMDA receptor antagonists were selected (i.e., **3e**, **3n**, **3c**, and **3m**) to evaluate their ability to cross the BBB. For this reason, we used the human brain microvascular endothelial cell line hCMEC/D3 as a suitable model for the normal human BBB [82]. Before that, we investigated whether the applied concentration (50 µM) of the tested compound negatively influenced the cell viability in the monolayer. Despite the relatively low toxicity of the CHO-K1 model, we investigated the effects on the cell viability of the hCMEC/D3 line using MTT at 100 and 50 µM (Table S3). We found that all tested compounds at 50 µM did not substantially reduce the cell viability (>90% of viable cells), except **3m**, which reduced the cell viability to approx. 82% at 50 µM. Due to this fact, a concentration of 30 µM was used for the BBB permeation test for **3m**.

To investigate the potential of the BBB permeation, we used a panel of reference drugs for which the BBB permeation in vivo is known and correlated the P*_app_* values to them. Thus, our measurements predict that the compounds **3c**, **3e**, and **3n** have a low potential to passively pass the BBB. On the other hand, the P*_app_* value for **3m** corresponds to those of standard drugs with high CNS permeability (Table 6).

### 3.8. In Silico Studies

**Figure 7:**
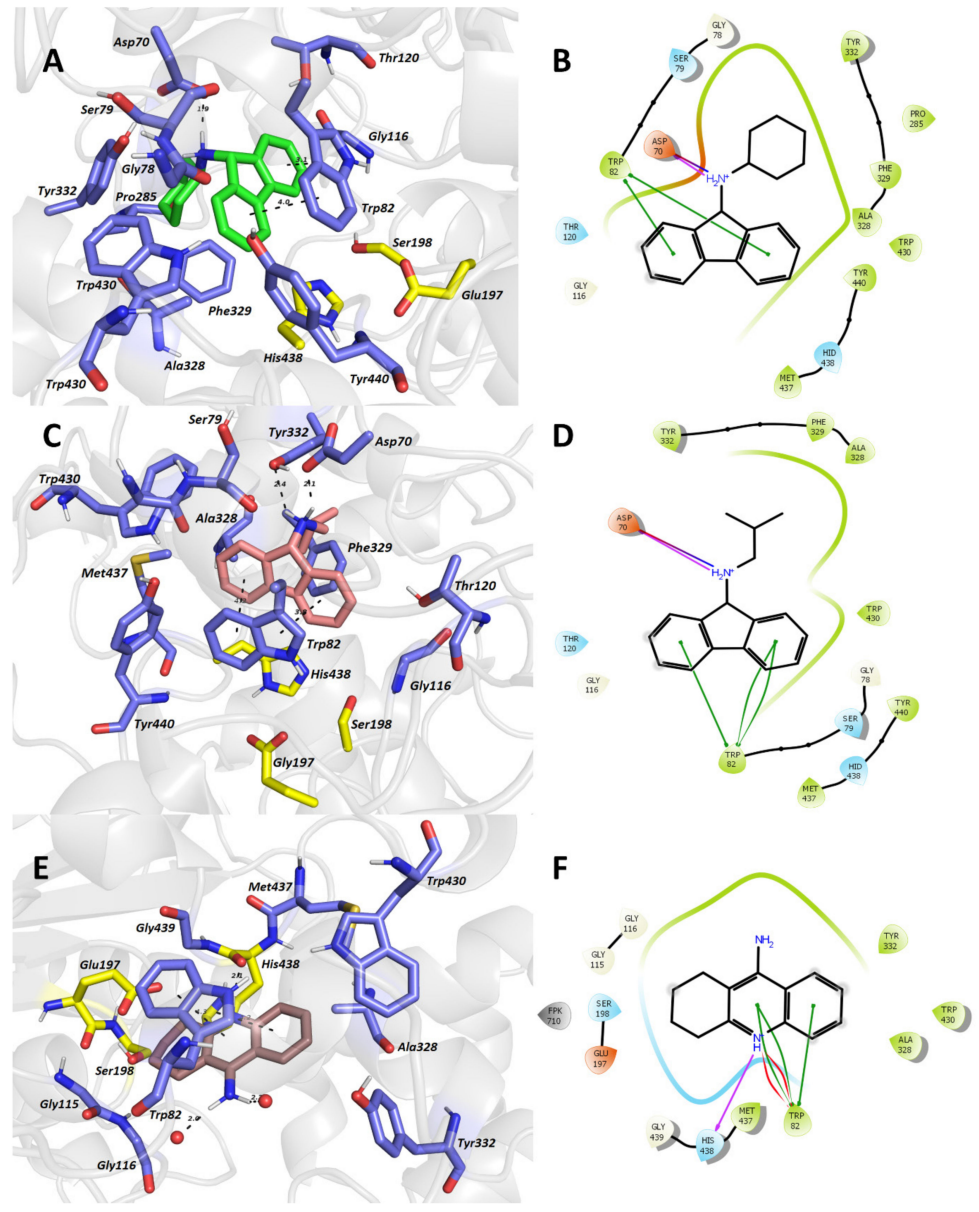
Top-scored docking poses of **3c** (**A**,**B**), **3n** (**C**,**D**), and the crystal structure of THA (**E**,**F**) within the *h*BChE active site (Protein Data Bank (PDB) ID: 4BDS) [56]. Ligands are displayed in green (**A**), salmon (**C**), and brown (**E**) for **3c**, **3n**, and THA, respectively. Important amino acid residues involved in the enzyme-ligand interaction are shown in dark-blue, and the catalytic triad is rendered in yellow. Distances are colored as dashed lines, and the distance is measured in Å (**A**,**C**,**E**). 3D figures were created by using PyMOL 2.0.6 viewer. 2D diagrams were generated with Maestro 12.3 (Schrödinger Release, Schrödinger, LLC., New York, NY, USA).

To better understand the inhibition mechanism between *h*BChE with **3c** (Figure 7A,B) and **3n** (Figure 7C,D), respectively, as the top-ranked inhibitors of the respective enzyme, we performed a molecular modeling simulation. The crystal structure of the THA-*h*BChE complex (Figure 7E,F; PDB ID: 4BDS) was used for comparative purposes [56]. In line with the compounds’ designs, both of the inspected fluoren-9-amines **3c** and **3n** adopted a similar arrangement to that of THA within the *h*BChE active site. The crucial observation is that the tricyclic cores of both new molecules interact with Trp82 via parallel π-π stacking in the anionic site of the enzyme. The protonated amino group enabled hydrogen bond formation to carboxylic oxygen from Asp70 (**3c** and **3n**) and the hydroxyl group from Tyr332 (**3n**). In the case of THA, this type of interaction is replaced by two water bridges with the primary amino group. From the catalytic triad residues (His438, Glu197, and Ser198), only His438 is involved in hydrophobic contact with **3c** and **3n**, whereas THA is involved in direct hydrogen contact with this residue. The docking results also highlighted the importance of the attached appendages. Both aliphatic parts, either the cyclohexyl (**3c**) or *iso*-butyl (**3n**) moiety, are engaged in hydrophobic contacts with the Tyr332, Phe329, and Ala328 residues. The cyclohexyl moiety of **3c** seems to deliver another hydrophobic contact to Pro285 and Gly78 that presumably mediates a slightly higher inhibition potency. Contrary to THA anchoring, missing cation-π contacts in the **3c**- and **3n**-*h*BChE complexes can be denoted as the culprit responsible for the slightly decreased affinity of these two agents.

## 4. Conclusions

Since the cholinergic hypothesis was first postulated [15], five decades of intensive research have generated a few marketed drugs for symptomatic relief only. The proper and in-depth elucidation of the disease biology imposes challenging tasks that remain to be resolved by delivering the drugs in the right stage of the disorder [29]. So far, the attempts to approve any drugs have continuously failed, with memantine receiving the final approval in 2003. A glimmer of hope emerged in 2019 when sodium oligomannate (GV-971) was approved in China, acting in a completely different way to other marketed drugs. Indeed, GV-971 suppresses gut dysbiosis, mitigates neuroinflammation, and reverses cognition impairment [83]. Designing MTDLs is an interesting approach that has generated several preclinical candidates for the therapy of AD. However, most of these newly developed agents suffer from limitations, as disclosed recently [29]. Our study was inspired by the appealing combination of donepezil and memantine into one capsule that resulted in a higher therapeutic benefit than the single-agent administrations [27]. Herewith, pursuing the symptomatic stage of AD by modulating the cholinergic and glutamatergic systems, we developed a series of 15 molecules with fluorene tricyclic scaffolds substituted with secondary or tertiary amines at C9. Prior to the synthesis, we took the compound’s predicted physicochemical parameters and calculated their oral and CNS availability. Indeed, all the compounds adhered to these criteria. Under in vitro conditions, we demonstrated that the compounds can interact with both NMDAR and BChE while having only a marginal effect on AChE. We also showed that they have a relatively low cytotoxic profile, as determined using CHO-K1 cell lines, and for one compound (**3m**) out of the four selected candidates, a BBB permeation ability was confirmed. The obtained data herein warrant future research with this class of compounds; however, we may state that we found low-toxic BChE-selective compounds with antagonistic activity on the NMDARs that potentially permeate the BBB.

## Figures and Tables

**Figure 6:**
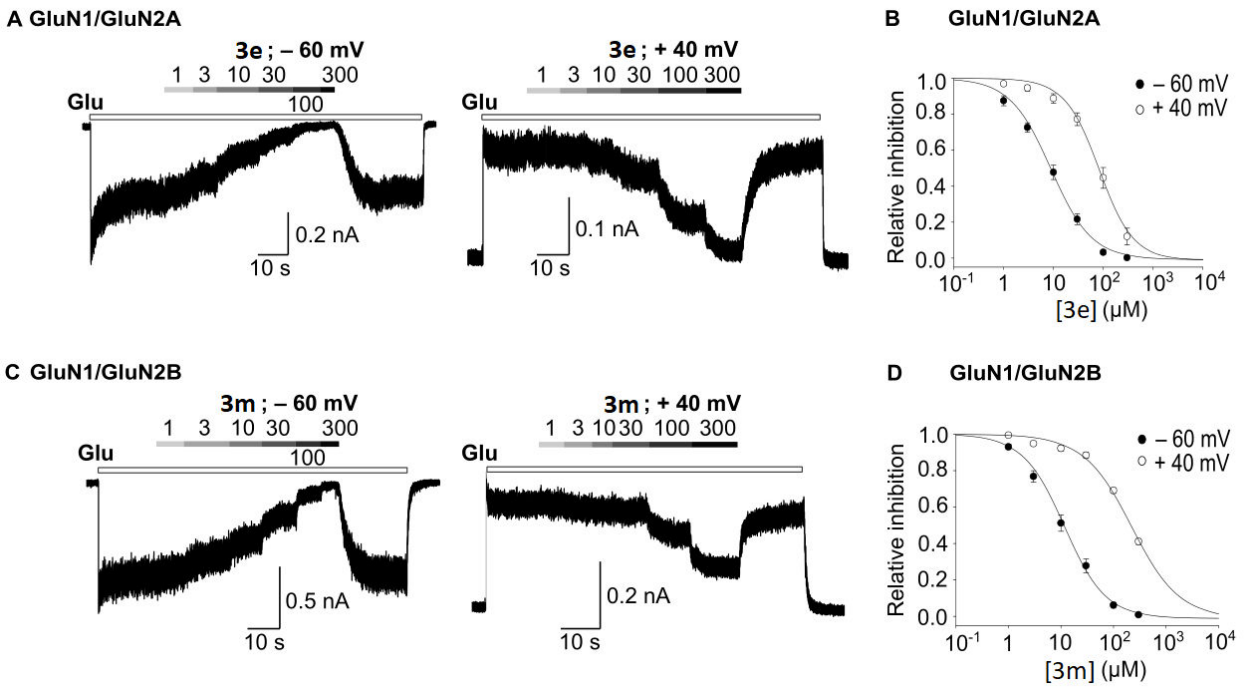
The most potent inhibitory compounds **3e** and **3m** acting at the GluN1/GluN2A and GluN1/GluN2B receptors. (**A**) Whole-cell representative currents of the GluN1/GluN2A receptors showing the inhibitory effects of **3e** (1–300 µM) at membrane potentials −60 mV and +40 mV. (**C**) Representative currents of the GluN1/GluN2B receptors inhibited by **3m** (1–300 µM) at membrane potentials −60 mV and +40 mV. (**B**,**D**) Dose-response inhibitory curves at two membrane potentials (−60 and +40 mV) at the GluN1/GluN2A receptors for **3e** (**B**) and at the GluN1/GluN2B receptors for **3m** (**D**). The inhibition dose-response curves were fitted by Equation (1) (see Section 2.5.2); the resulting values are shown in Table 4.

**Table 1:** Summary of the in silico absorption, distribution, metabolism, and excretion (ADME) and drug-likeness of derivatives **3a**–**o** with tacrine (THA) and memantine using various prediction models.

Compound	GIA ^a^	BBB ^b^	BBB Score ^c^	Lipinski ^d^	Bio. Score ^e^
**3a**	High	Yes	5.63	Yes	0.55
**3b**	High	Yes	5.63	Yes	0.55
**3c**	High	Yes	5.59	Yes	0.55
**3d**	High	Yes	5.64	Yes	0.55
**3e**	High	Yes	5.93	Yes	0.55
**3f**	High	Yes	5.15	Yes	0.55
**3g**	High	Yes	5.68	Yes	0.55
**3h**	High	Yes	5.63	Yes	0.55
**3i**	High	Yes	5.17	Yes	0.55
**3j**	High	Yes	5.67	Yes	0.55
**3k**	High	Yes	5.86	Yes	0.55
**3l**	High	Yes	5.55	Yes	0.55
**3m**	High	Yes	5.62	Yes	0.55
**3n**	High	Yes	5.62	Yes	0.55
**3o**	High	Yes	5.72	Yes	0.55
**THA**	High	Yes	5.38	Yes	0.55
**Memantine**	High	Yes	4.61	Yes	0.55

^a^ GIA = gastrointestinal absorption, ^b^ BBB = blood–brain barrier permeation, ^c^ Reference [63], ^d^ Reference [68], and ^e^ Bio. Score = bioavailability score [69].

**Table 2:** The inhibitory activities of compounds **3a**–**o** for human acetylcholinesterase (*h*AChE) and human butyrylcholinesterase (*h*BChE).

Compound	IC_50_ ± SEM (µM) ^a^		SI for BChE ^b^
	*h*AChE	*h*BChE	
**3a**	n.a.	2.95 ± 0,09	-
**3b**	n.a.	8.91 ± 0.31	-
**3c**	13.98 ± 0.35	0.47 ± 0.01	29.7
**3d**	n.a.	6.42 ± 0.22	-
**3e**	n.a.	2.03 ± 0.08	-
**3f**	n.a.	2.13 ± 0.06	-
**3g**	n.a.	2.56 ± 0.11	-
**3h**	n.a.	6.58 ± 0.19	-
**3i**	n.a.	5.38 ± 0.11	-
**3j**	n.a.	23.5 ± 0.8	-
**3k**	n.a.	12.8 ± 0.3	-
**3l**	n.a.	54.7 ± 1.9	-
**3m**	49.91 ± 3.05	1.02 ± 0.03	48.9
**3n**	n.a.	0.90 ± 0,02	-
**3o**	n.a.	1.80 ± 0.06	-
**THA ^c^**	0.50 ± 0.10 ^c^	0.023 ± 0.003 ^c^	21.7

^a^ Results are expressed as the mean of at least three experiments ± SEM. n.a. stands for not active at the tested concentration scale. ^b^ SI = selectivity index; the selectivity for *h*BChE is determined as a ratio of the half-maximal inhibitory concentration (IC_50_)(*h*AChE)/IC_50_(*h*BChE). ^c^ Data taken from Reference [79].

**Table 3:** The inhibitory effect of the derivatives **3a**–**o** at the GluN1/GluN2A and GluN1/GluN2B receptors.

Compound	GluN1/GluN2A		GluN1/GluN2B	
	RI^a^ (%) ± SEM	n	RI^a^ (%) ± SEM	n
**3a**	33.29 ± 1.66	4	25.00 ± 1.82	4
**3b**	36.16 ± 2.16	4	18.01 ± 0.78	4
**3c**	41.02 ± 3.56	5	17.24 ± 2.39	4
**3d**	26.56 ± 0.85	5	14.26 ± 0.35	5
**3e**	52.30 ± 4.18	5	44.79 ± 3.43	5
**3f**	33.01 ± 1.96	5	19.35 ± 2.65	5
**3g**	18.68 ± 1.98	5	6.76 ± 1.00	4
**3h**	26.90 ± 2.14	5	17.48 ± 2.46	5
**3i**	25.66 ± 1.92	5	20.12 ± 2.82	5
**3j**	29.15 ± 2.85	4	21.88 ± 2.10	4
**3k**	32.08 ± 1.01	4	19.12 ± 1.48	6
**3l**	14.89 ± 1.76	4	11.64 ± 0.52	5
**3m**	36.23 ± 3.73	6	51.22 ± 3.24	5
**3n**	37.87 ± 2.43	6	49.71 ± 1.72	5
**3o**	27.80 ± 1.74	5	32.39 ± 2.99	5

^a^ The relative inhibition (RI) was calculated as the ratio of the steady-state currents in the presence and absence of 10 µM of the compound (multiplied by 100) at the membrane potential of −60 mV (see text for more details). Data are shown as mean ± SEM; n = number of recorded cells (see Figure 5).

**Table 4:** The activity of derivatives **3e** and **3m** at the *N*-methyl-d-aspartate (NMDA) receptors at different membrane potentials compared to THA and memantine (Mem).

	GluN1/GluN2A ^a^					GluN1/GluN2B ^a^				
	IC_50_ −60 mV	*h* −60 mV	IC_50_ 40 mV	*h* 40 mV	n (−60/40 mV)	IC_50_ −60 mV	*h* −60 mV	IC_50_ 40 mV	*h* 40 mV	n (−60/40 mV)
**3e**	8.85 ± 1.21	1.07 ± 0.05	82.62 ± 14.17	1.28 ± 0.12	8/5	15.27 ± 2.38	1.17 ± 0.05	156.40 ± 14.94	1.10 ± 0.08	12/7
**3m**	14.21 ± 1.60	1.19 ± 0.14	113.99 ± 26.63	1.23 ± 0.13	5/4	11.67 ± 1.72	1.12 ± 0.05	220.78 ± 12.24	0.97 ± 0.10	9/6
**THA ^b^**	9.1 ± 0.5	1.7 ± 0.0	84.6 ± 1.6	1.6 ± 0.1	8/4	19.7 ± 1.8	1.8 ± 0.1	168.8 ± 9.3	1.4 ± 0.1	9/6
**Mem ^b^**	1.34 ± 0.08	1.04 ± 0.06	27.04 ± 1.19	1.16 ± 0.05	6/7	0.78 ± 0.09	1.04 ± 0.03	10.57 ± 0.63	0.84 ± 0.04	5/6

^a^ The experimental data obtained using six concentrations of each compound (1, 3, 10, 30, 100, and 300 µM) were fitted by Equation (1) (see Section 2.5.2); the values of the IC_50_ (in µM), Hill coefficient (*h*), and numbers of analyzed cells (n) obtained from the membrane potentials of −60 mV or 40 mV are shown as mean ± SEM. Dose-response relationships of the inhibitory effects of **3e** or **3m** at the GluN1/GluN2A and GluN1/GluN2B receptors (see Figure 6). ^b^ THA values were published by our group recently [39]. Mem corresponds to memantine.

**Table 5:** The effects of the tested compounds on the CHO-K1 cell viability.

Compound.	IC_50_ ± SEM (µM) ^a^	Clog*P*
**3a**	387 ± 32	3.76
**3b**	427 ± 12	2.99
**3c**	111 ± 10	4.79
**3d**	353 ± 32	3.46
**3e**	192 ± 3	3.90
**3f**	872 ± 10	4.22
**3g**	738 ± 1	2.94
**3h**	417 ± 16	3.35
**3i**	266 ± 3	4.09
**3j**	259 ± 12	3.22
**3k**	239 ± 35	3.58
**3l**	>800 ^b^	3.15
**3m**	140 ± 15	4.31
**3n**	236 ± 24	4.29
**3o**	413 ± 15	3.78
**THA**	248 ± 11 ^c^	2.63

^a^ Values are expressed as the mean of three independent measurements. ^b^ Determined at the maximum solubility of the compound. ^c^ Data taken from Reference [81]. Clog*P*: calculated log*P*.

**Table 6:** Prediction of the BBB penetration of drugs expressed as the apparent permeability coefficient (P*_app_*) ± SEM.

Compound	P*_app_* ± SEM (×10^−6^ cm s^−1^)	CNS (+/−) ^a^	N ^b^
**3e**	13.51 ± 1.54	CNS −	3
**3n**	14.61 ± 1.88	CNS −	3
**3c**	12.28 ± 2.33	CNS −	3
**3m**	18.48 ± 2.37	CNS +	4
THA	22.29 ± 2.01	CNS +	4
Donepezil	24.19 ± 2.83	CNS +	4
Rivastigmine	28.63 ± 4.64	CNS +	5
Propranolol	23.06 ± 2.06	CNS +	5
Antipyrine	17.19 ± 1.82	CNS +	3
Furosemide	13.89 ± 0.92	CNS −	3
Obidoxime	14.35 ± 0.52	CNS −	4

^a^ CNS+ and CNS− represent the final predictions of the compound capability to cross or not to cross the BBB, respectively. ^b^ n stands for the number of independent experiments.

## Data Availability

Data is contained within the article or Supplementary Materials.

## Supplementary Materials

The following are available online at https://www.mdpi.com/2218-273X/11/1/3/s1, Table S1. Physiochemical parameters of compounds **3a**–**o**, THA and Memantine.; Table S2. Drug-likeness of derivatives **3a**–**o**, THA and memantine.; Table S3. Effect of tested compounds on the cell viability of the hCMEC/D3 cells.; ^1^H and ^13^C NMR spectra; HRMS, and HPLC records of compounds.
