# Supplementary material for: Pursuing the Complexity of Alzheimer’s Disease: Discovery of Fluoren-9-Amines as Selective Butyrylcholinesterase Inhibitors and N-Methyl-d-Aspartate Receptor Antagonists
Source: Biomolecules. 2020 Dec 22;11(1):3. doi: 10.3390/biom11010003 (PMC7822176; doi:10.3390/biom11010003)
Supplement: Supplementary file 1 [file biomolecules-11-00003-s001.pdf]

Supplementary Materials

Pursuing the Complexity of Alzheimer's Disease: Discovery of Fluoren-9-Amines as Selective Butyrylcholinesterase Inhibitors and *N*-Methyl-D-Aspartate Receptor Antagonists

Jan Konecny [1,2,†], Anna Misiachna [3,4,5,†], Martina Hrabinova [1,2], Lenka Pulkrabkova [1,2], Marketa Benkova [2], Lukas Prchal [2], Tomas Kucera [1,2], Tereza Kobrlova [2], Vladimir Finger [2,6], Marharyta Kolcheva [3,4], Stepan Kortus [3,4], Daniel Jun [1,2], Marian Valko [7], Martin Horak [3,4], Ondrej Soukup [1,2,*] and Jan Korabecny [1,2,*]

1 Department of Toxicology and Military Pharmacy, Faculty of Military Health Sciences, Trebesska 1575, 500 01 Hradec Kralove, Czech Republic, jan.konecny@unob.cz (J.K.), martina.hrabinova@unob.cz (M.H.), lenka.pulkrabkova@fnhk.cz (L.P.), tomas.kucera2@unob.cz (T.K.), daniel.jun@unob.cz (D.J.)
2 Biomedical Research Centre, University Hospital Hradec Kralove, Sokolska 581, 500 05 Hradec Kralove, Czech Republic, Marketa.Benkova@fnhk.cz (M.B.), lukas.prchal@fnhk.cz (L.P.), tereza.kobrlova@fnhk.cz (T.K.), fingerv@faf.cuni.cz (V.F.)
3 Institute of Experimental Medicine of the Czech Academy of Sciences, Videnska 1083, 14220 Prague 4, Czech Republic, anna.misiachna@iem.cas.cz (A.M.), marharyta.kolcheva@iem.cas.cz (M.K.), stepan.kortus@iem.cas.cz (S.K.), martin.horak@iem.cas.cz (M.H.)
4 Institute of Physiology of the Czech Academy of Sciences, Videnska 1083, 14220 Prague 4, Czech Republic
5 Department of Physiology, Faculty of Science, Charles University in Prague, Albertov 6, 12843 Prague 2, Czech Republic
6 Department of Organic and Bioorganic Chemistry, Charles University, Faculty of Pharmacy in Hradec Kralove, Akademika Heyrovskeho 1203, 50005, Hradec Kralove, Czech Republic
7 Slovak University of Technology, Faculty of Chemical and Food Technology, Radlinskeho 9, 812 37 Bratislava, Slovakia, marian.valko@stuba.sk
* Correspondence: ondrej.soukup@fnhk.cz (O.S.); jan.korabecny@fnhk.cz (J.K.) Tel.: +420-495-833-447 (O.S.); +420-495-833-447 (J.K.)
† These authors contributed equally to this paper.

Table of contents

1. **Physiochemical properties and drug-likeness of final compounds** 1
2. **MTT cell viability test prior the BBB evaluation using hCMEC/D3 cells** 3
3. **^{1}H and ^{13}C NMR spectra** 4
4. **HPLC and LC-MS spectra of final compounds** 34
5. **References** 49

1. Physiochemical properties and drug-likeness of final compounds

For the prediction of physiochemical properties (Table S1) was used MarvinSketch 20.4.0, ChemAxon Ltd. Drug-likeness of designed derivatives was evaluated by SwissADME online tool.[1]

Table S1. Physiochemical parameters of compounds **3a-o**, THA and Memantine.

Compound	MW	pKa	logP	HBA	HBD	TPSA
**3a**	259.78	9.06	3.76	1	1	12.03
**3b**	231.72	8.89	2.99	1	1	12.03
**3c**	299.84	9.34	4.79	1	1	12.03
**3d**	257.76	8.65	3.46	1	1	12.03
**3e**	271.79	8.96	3.90	1	1	12.03
**3f**	285.82	9.66	4.22	1	0	3.24
**3g**	275.78	8.33	2.94	2	1	21.26
**3h**	245.70	8.97	3.35	1	1	12.03
**3i**	273.80	9.60	4.09	1	0	3.24
**3j**	337.29	8.32	3.22	2	0	6.48
**3k**	314.85	8.39	3.58	2	0	6.48
**3l**	287.79	7.45	3.15	2	0	12.47
**3m**	273.80	9.15	4.31	1	1	12.03
**3n**	273.80	9.22	4.29	1	1	12.03
**3o**	271.78	9.80	3.78	1	0	3.24
**THA**	198.26	8.95	2.63	2	1	38.91
**Memantine**	179.30	10.70	2.07	1	1	26.02

MW = molecular weight, pKa = acid dissociation constant, logP = partition coefficient, HBA = hydrogen bond acceptor, HBB = hydrogen bond donor, TPSA = topological polar surface area

Table S2. Drug-likeness of derivatives **3a-o**, THA and memantine.

Compound	Drug-likeness				
	Lipinski [a]	Ghose [b]	Veber [c]	Egan [d]	Muegge [e]
**3a**	Yes	Yes	Yes	Yes	No [f]
**3b**	Yes	Yes	Yes	Yes	No [g]
**3c**	Yes	Yes	Yes	Yes	No [f]
**3d**	Yes	Yes	Yes	Yes	No [f]
**3e**	Yes	Yes	Yes	Yes	No [f]
**3f**	Yes	Yes	Yes	Yes	No [f]
**3g**	Yes	Yes	Yes	Yes	Yes
**3h**	Yes	Yes	Yes	Yes	No [f]
**3i**	Yes	Yes	Yes	Yes	No [f]
**3j**	Yes	Yes	Yes	Yes	Yes
**3k**	Yes	Yes	Yes	Yes	Yes
**3l**	Yes	Yes	Yes	Yes	Yes
**3m**	Yes	Yes	Yes	Yes	No [f]
**3n**	Yes	Yes	Yes	Yes	No [f]
**3o**	Yes	Yes	Yes	Yes	No [f]
THA	Yes	Yes	Yes	Yes	No [f]
Memantine	Yes	Yes	Yes	Yes	No [g]

[a] Lipinski represent,[2] MW < 500 Da, logP <5, HBD <5, HBA <10; [b] Ghose,[3] MW = 180 – 480, MR (molar refractivity) = 40 – 130, logP = -0.4 – +5.6, number of atoms = 20 – 70; [c] Veber,[4] rotatable bonds <10, TPSA <140; [d] Egan,[5] TPSA <132, logP <5.88; [e] Muegge,[6] MW = 200 – 500, log P = -2 - +5, HBD <5, HBA <10, MR = 40 – 130, rotatable bonds <8, heavy atoms = 20 – 70, TPSA <120, net charge -2 - +2; [f] 1 violation, [g] 2 violations.

2. MTT cell viability test prior the BBB evaluation using hCMEC/D3 cells

Table S3. Effect of tested compounds on the cell viability of the hCMEC/D3 cells

	Conc.	% viable cells	SD	n
**3c**	**100 µM**	82.87	11.19	3
	**50 µM**	93.44	1.66	4
**3e**	**100 µM**	92.34	9.43	3
	**50 µM**	92.79	4.11	3
**3m**	**100 µM**	80.10	6.22	3
	**50 µM**	82.08	4.34	4
**3n**	**100 µM**	92.67	11.81	3
	**50 µM**	98.20	20.59	3
**Tacrine**	**100 µM**	94.47	22,25	4
	**50 µM**	91.11	17.02	3

3. ^{1}H and ^{13}C NMR spectra

^{1}H NMR of *N*-propan-2-yl-9*H*-fluoren-9-amine hydrochloride (3a):

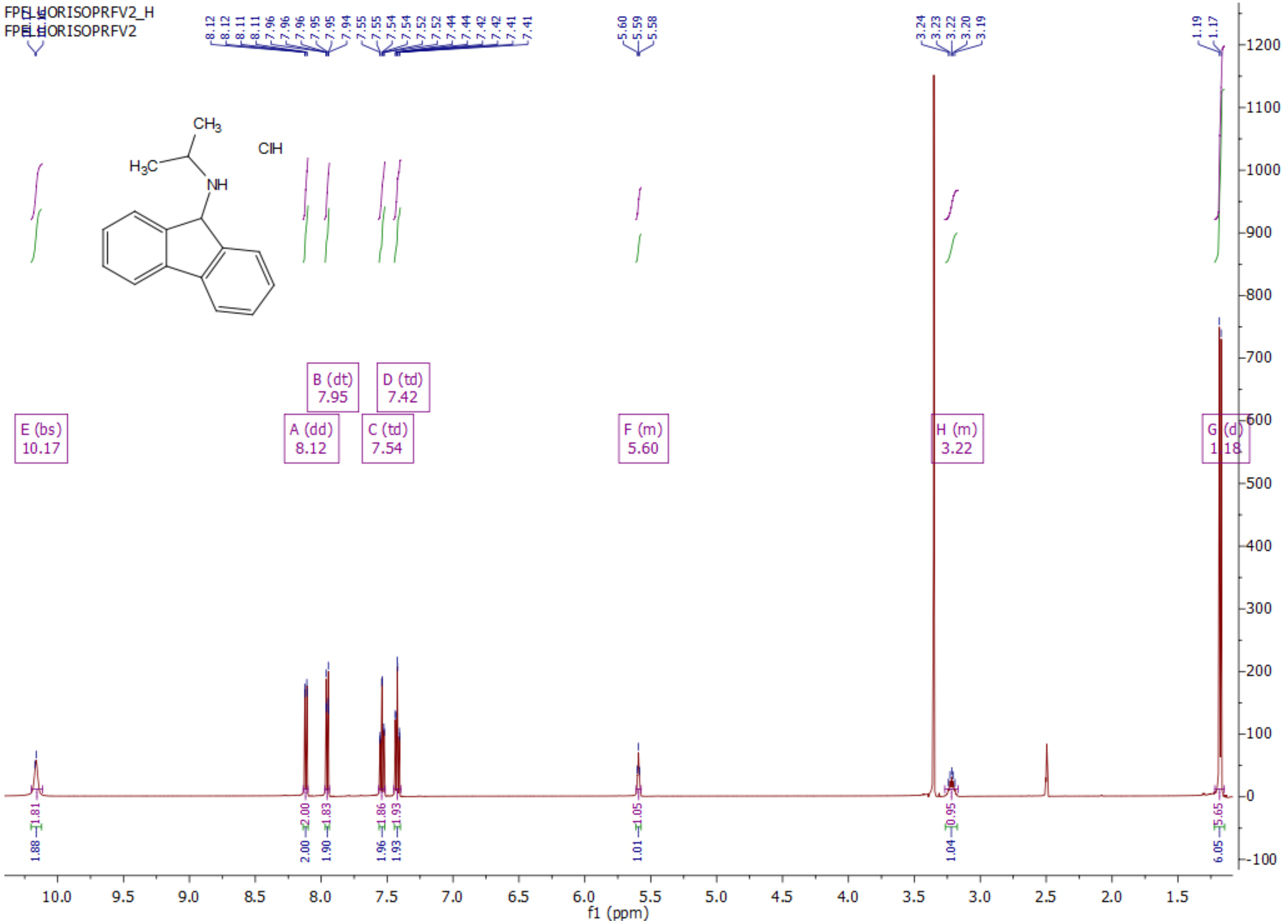

^{13}C NMR of *N*-propan-2-yl-9*H*-fluoren-9-amine hydrochloride (3a):

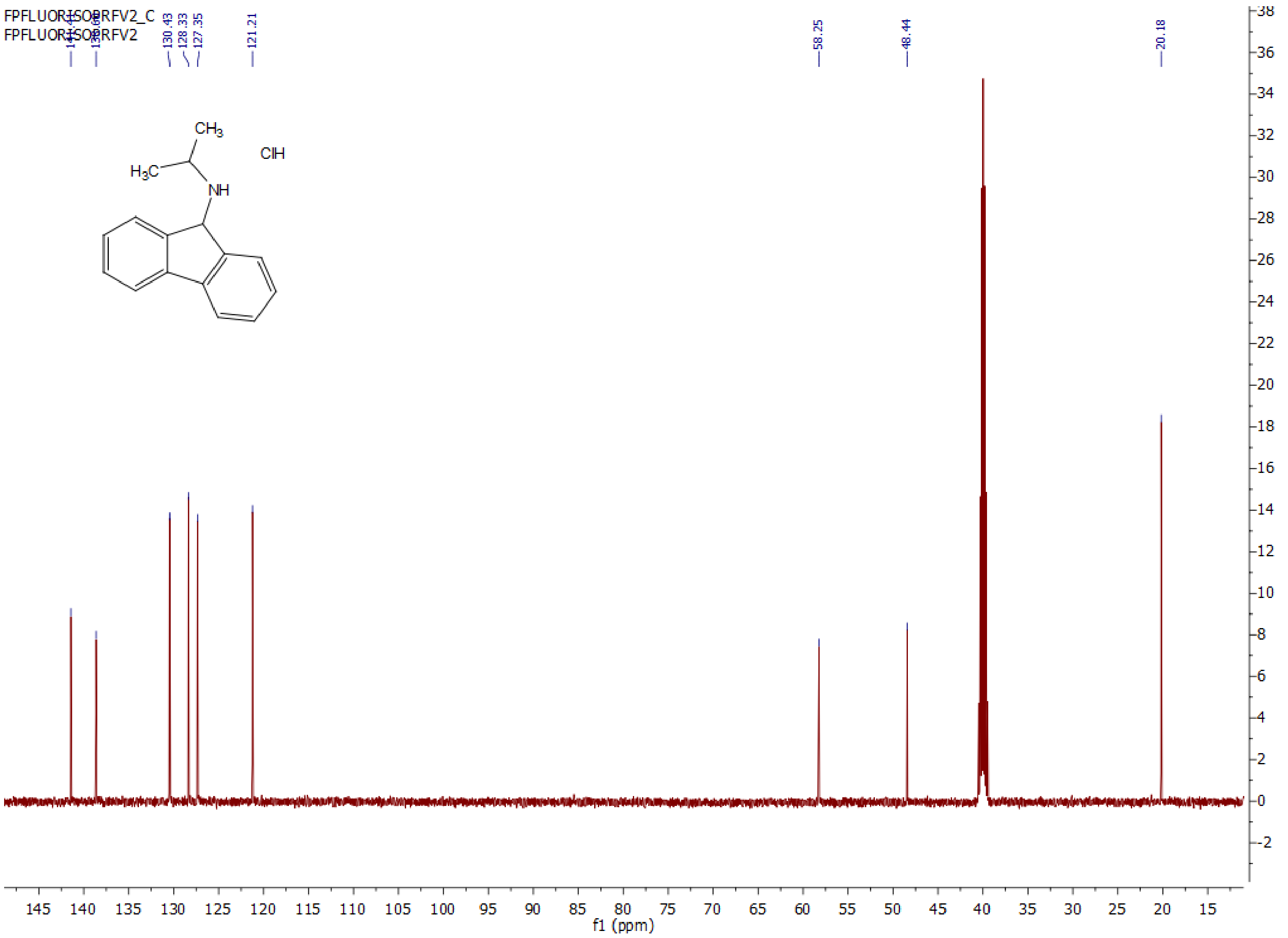

^{1}H NMR of *N*-methyl-9*H*-fluoren-9-amine hydrochloride (3b):

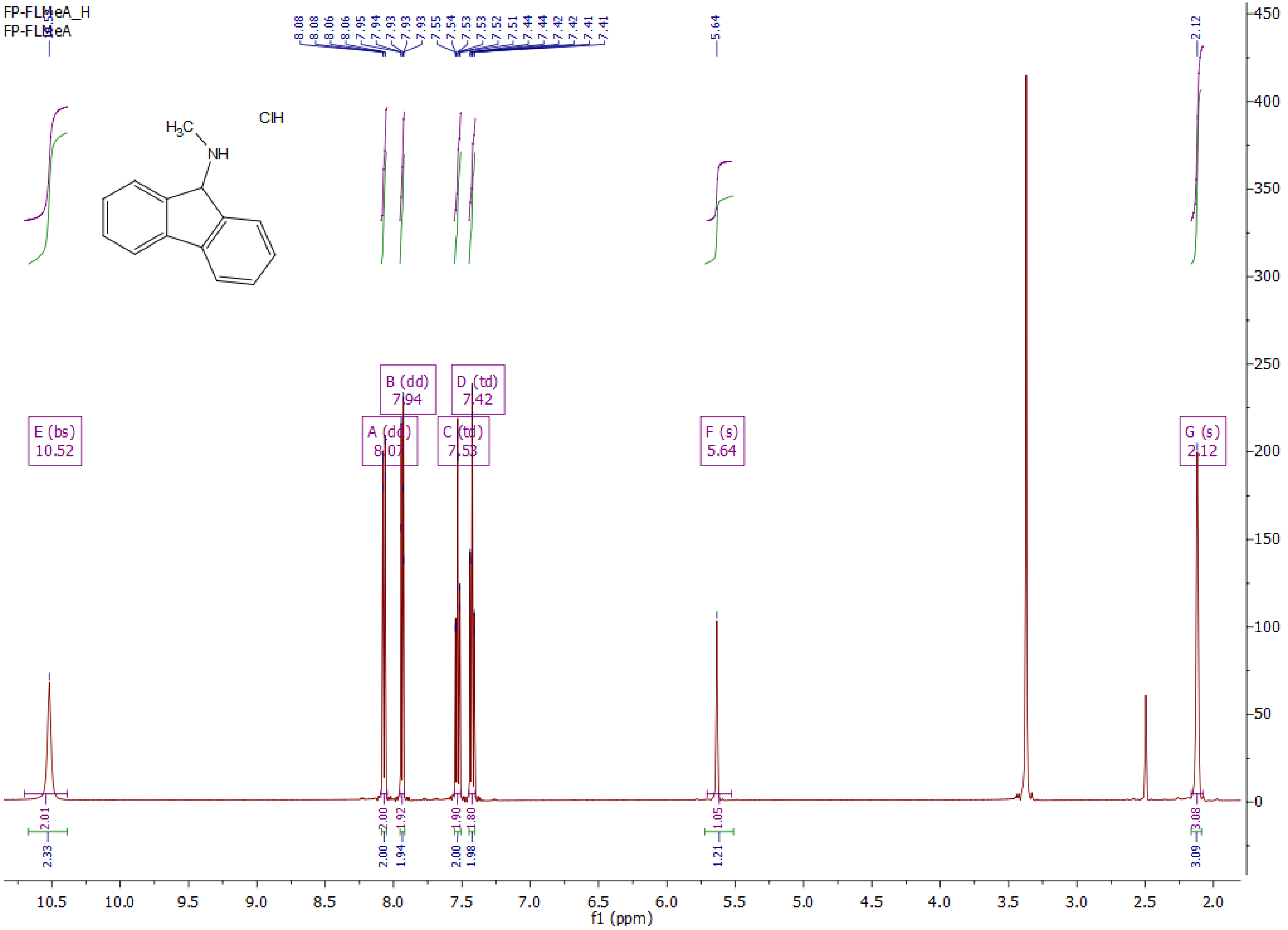

^{13}C NMR of *N*-methyl-9*H*-fluoren-9-amine hydrochloride (3b):

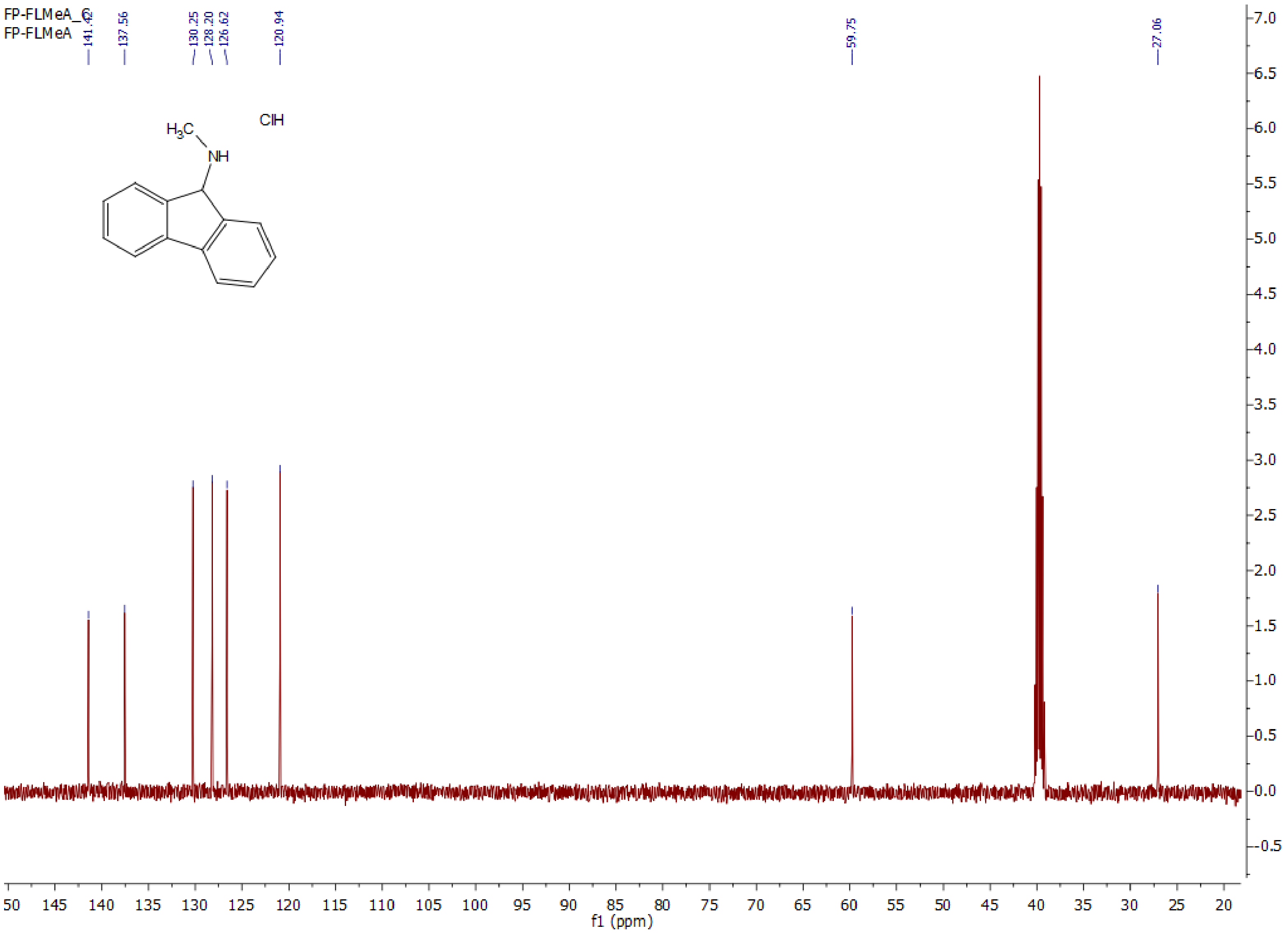

^{1}H NMR of *N*-cyclohexyl-9*H*-fluoren-9-amine hydrochloride (3c):

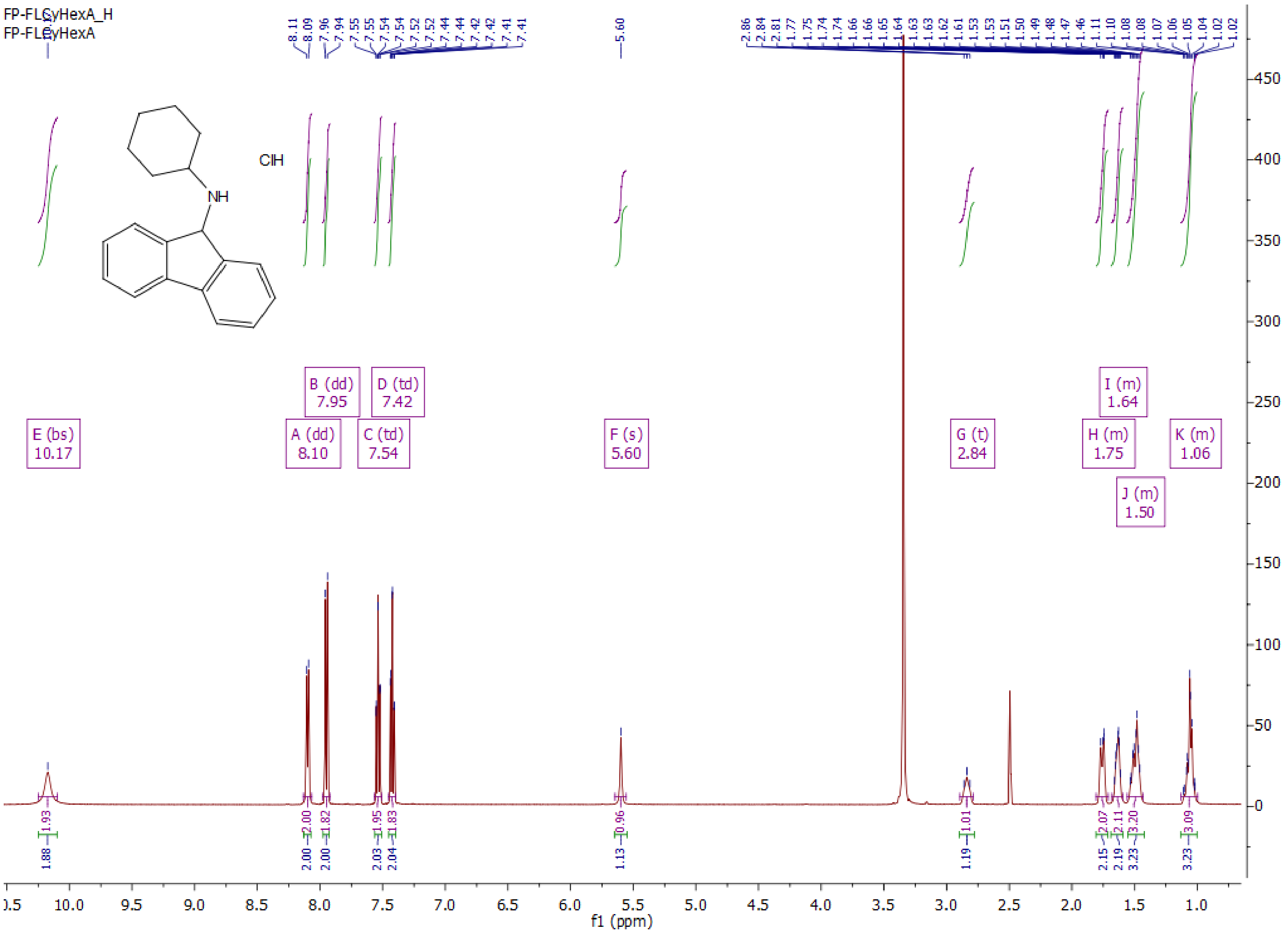

^{13}C NMR of *N*-cyclohexyl-9*H*-fluoren-9-amine hydrochloride (3c):

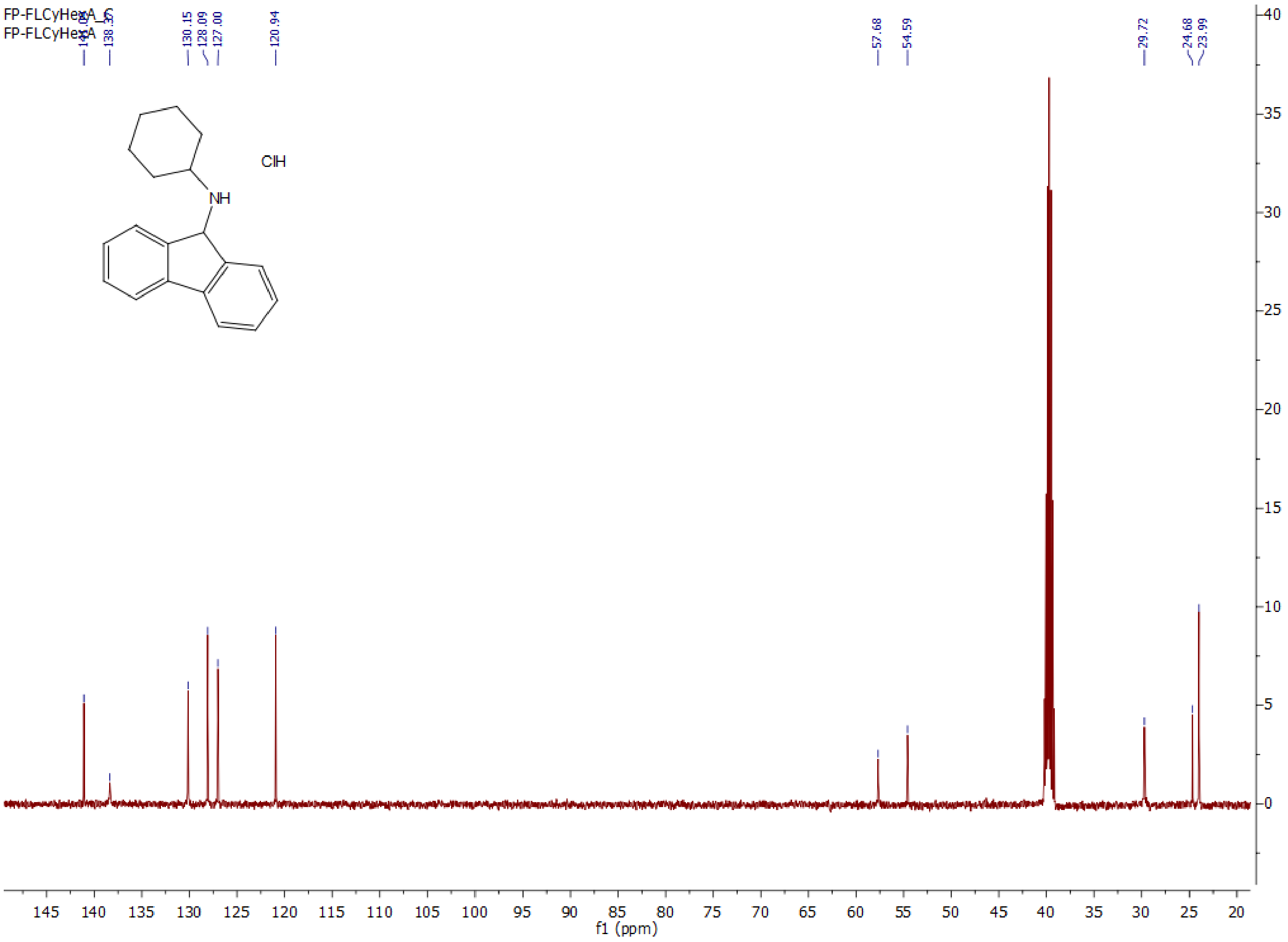

¹H NMR of *N*-cyclopropyl-9*H*-fluoren-9-amine hydrochloride (3d):

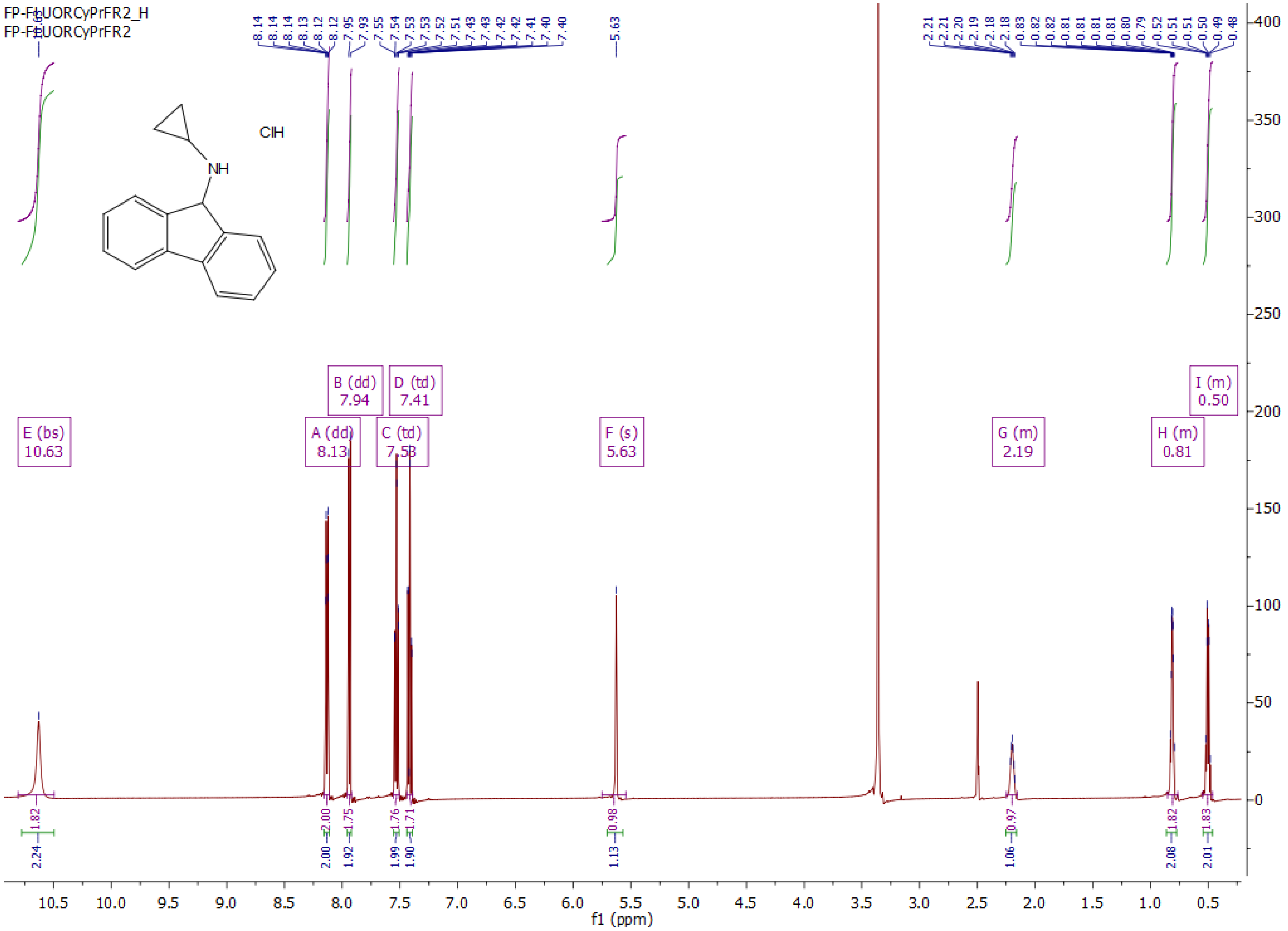

^{13}C NMR of *N*-cyclopropyl-9*H*-fluoren-9-amine hydrochloride (3d):

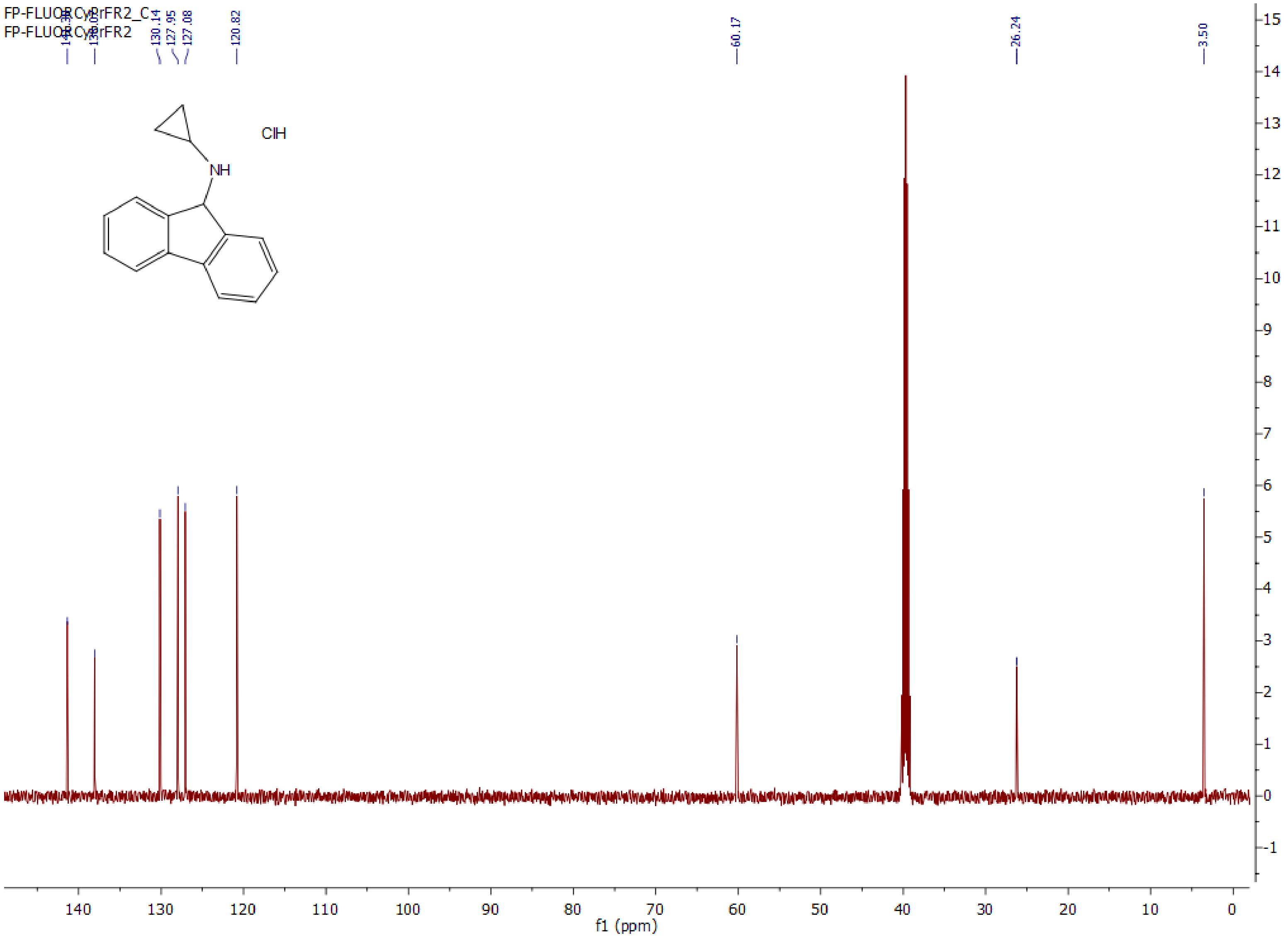

^{1}H NMR of *N*-cyclobutyl-9*H*-fluoren-9-amine hydrochloride (3e):

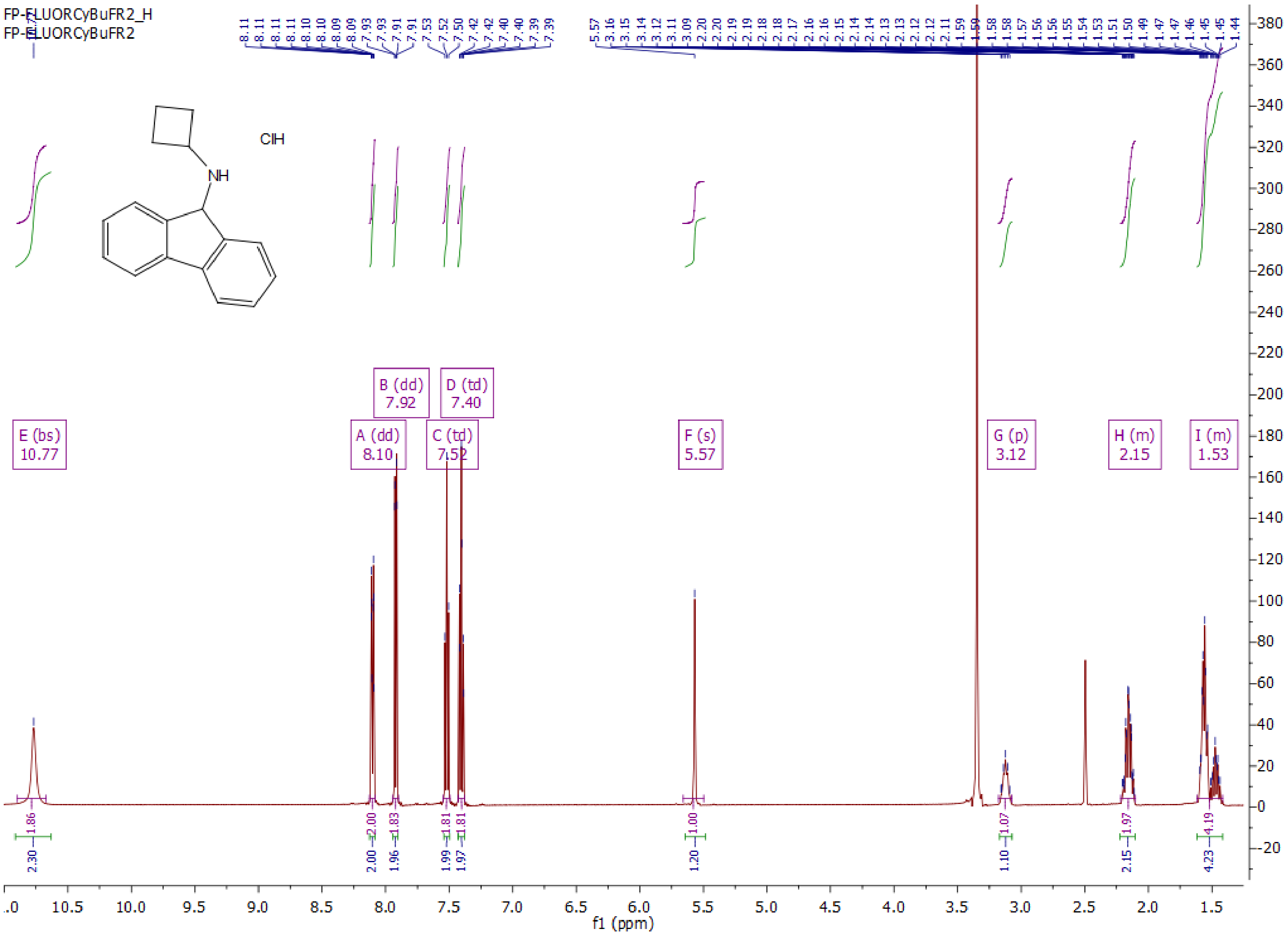

^{13}C NMR of *N*-cyclobutyl-9*H*-fluoren-9-amine hydrochloride (3e):

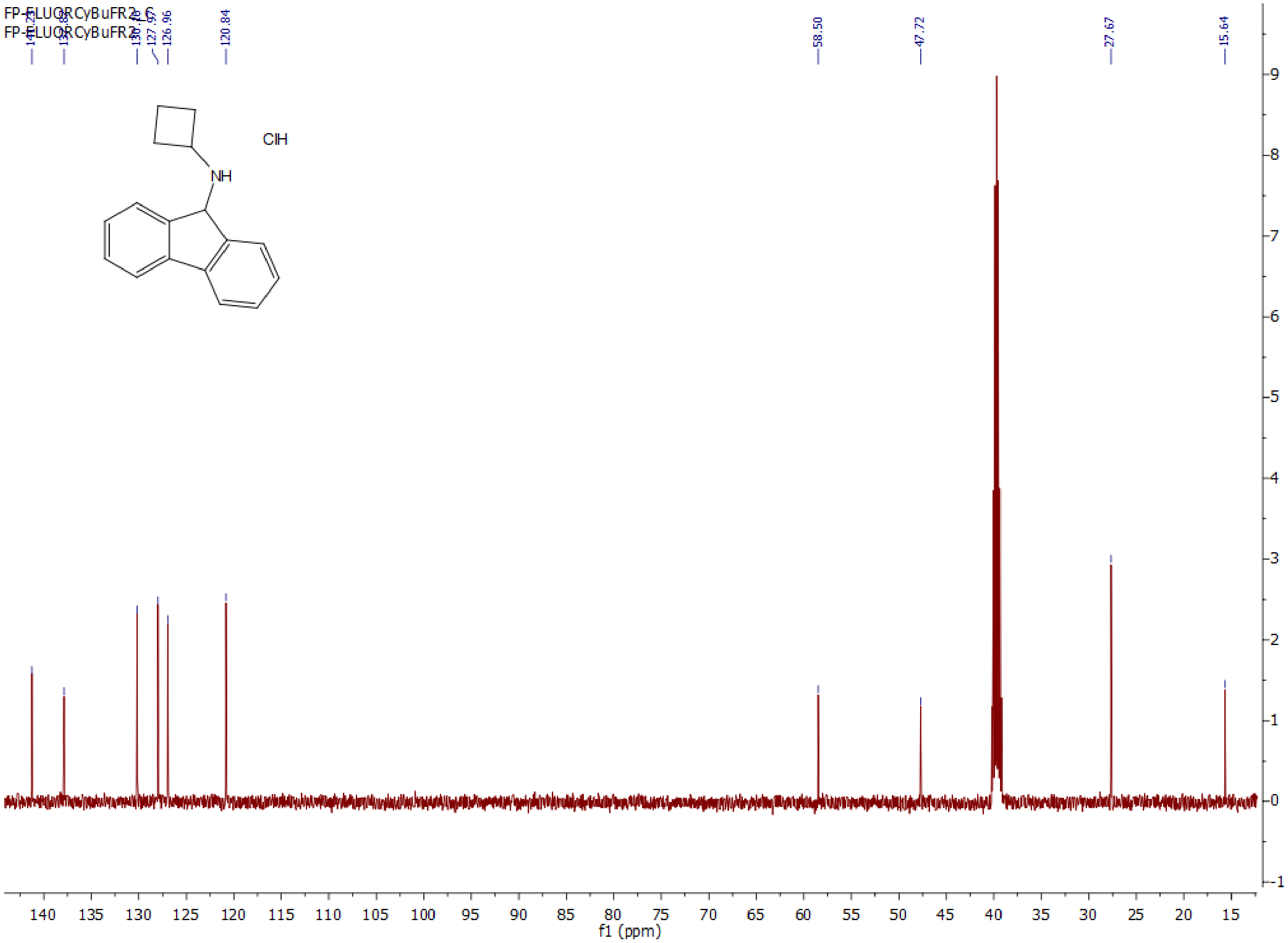

^{1}H NMR of 1-(9*H*-fluoren-9-yl)piperidine hydrochloride (3f):

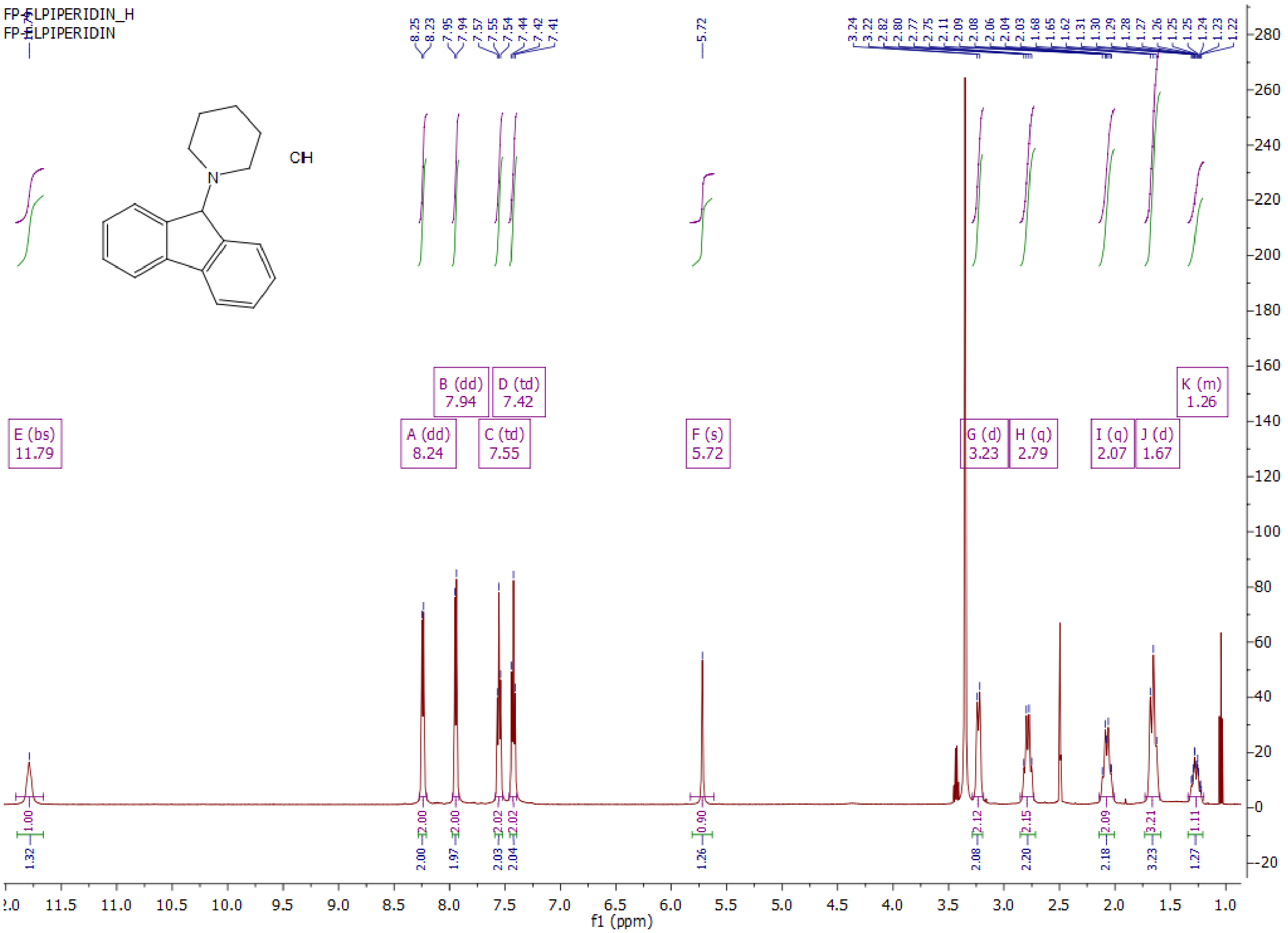

^{13}C NMR of 1-(9*H*-fluoren-9-yl)piperidine hydrochloride (3f):

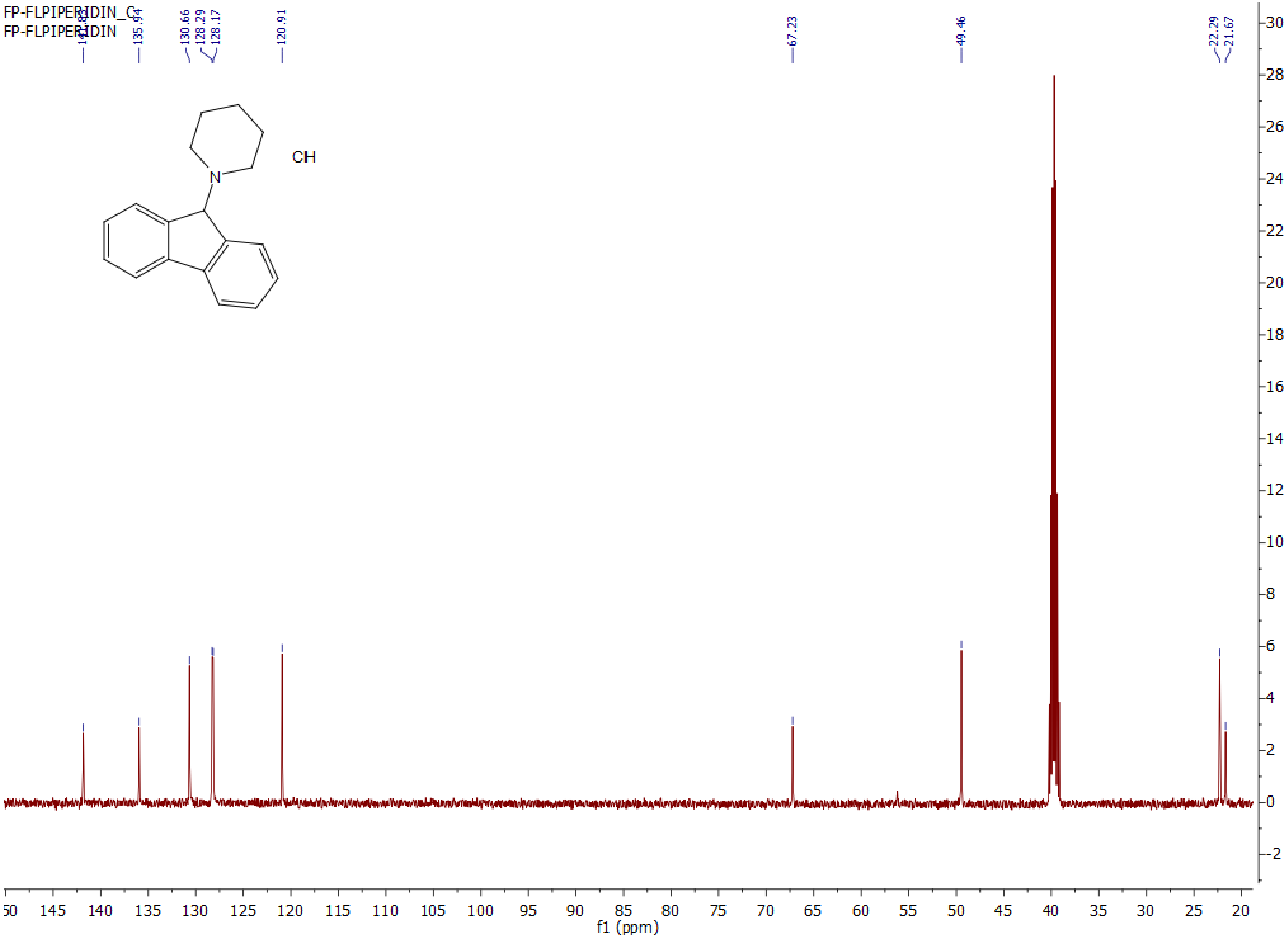

^{1}H NMR of *N*-(2-methoxyethyl)-9*H*-fluoren-9-amine hydrochloride (3g):

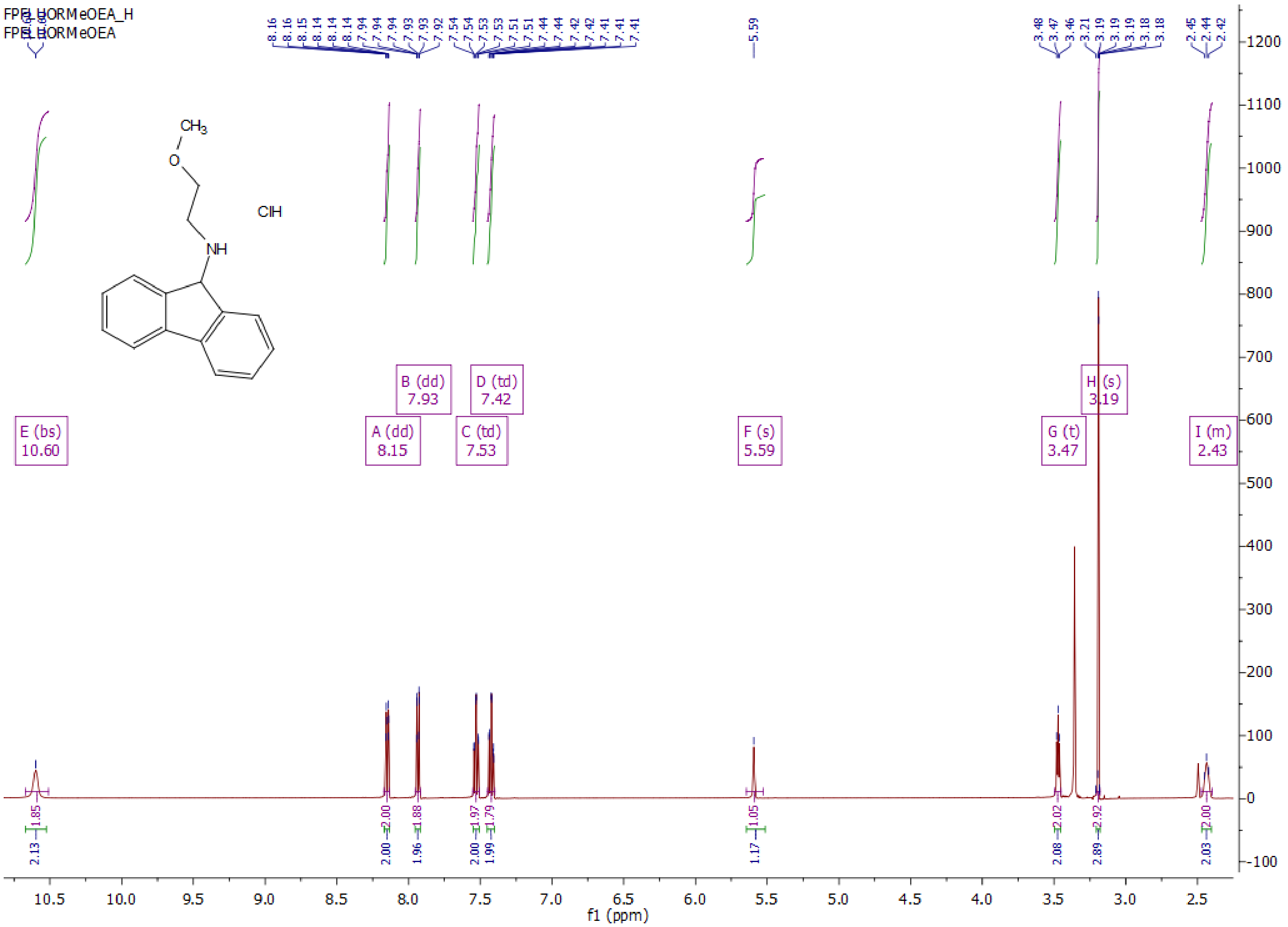

^{13}C NMR of *N*-(2-methoxyethyl)-9*H*-fluoren-9-amine hydrochloride (3g):

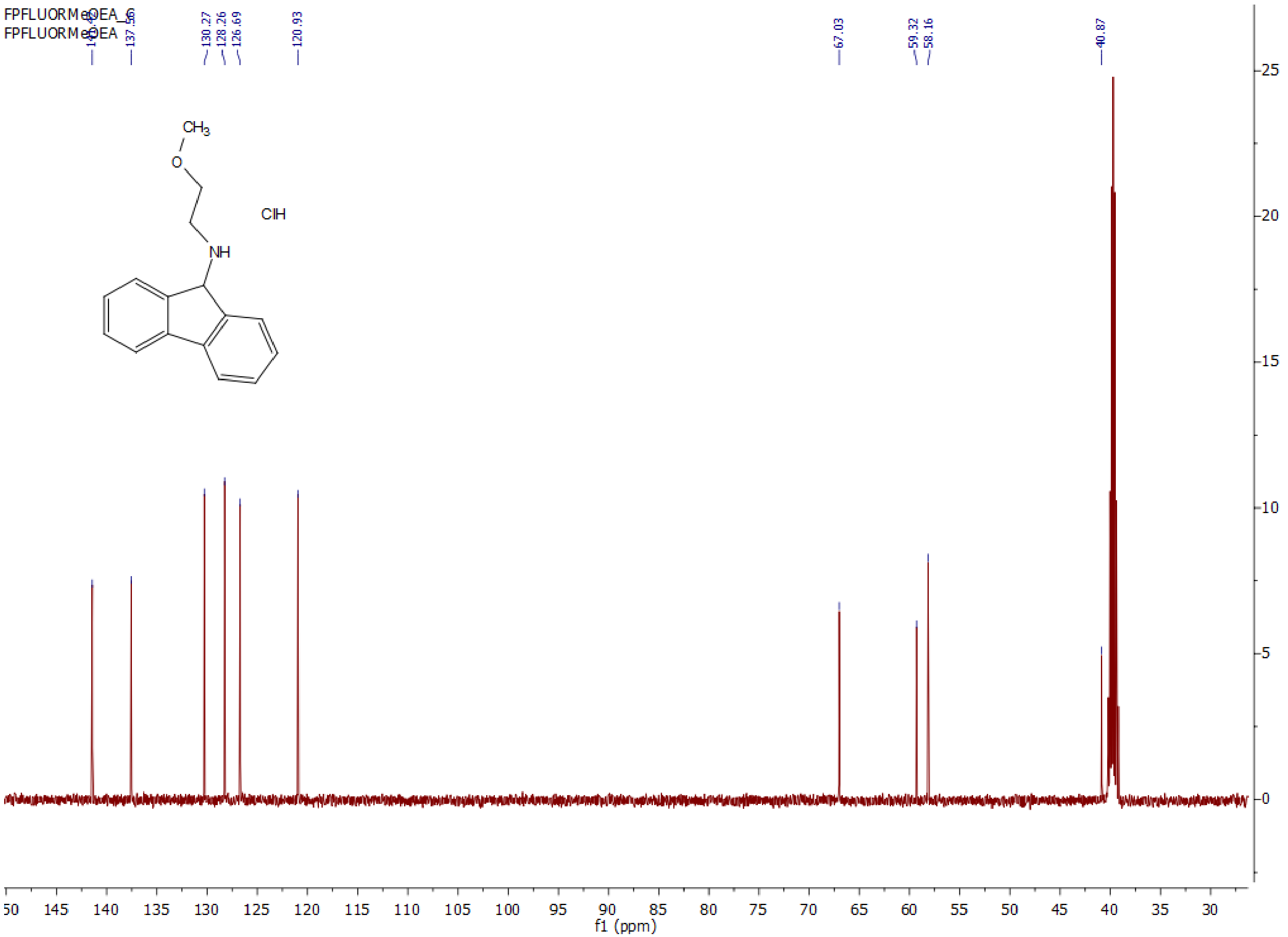

^{1}H NMR of *N*-ethyl-9*H*-fluoren-9-amine hydrochloride (3h):

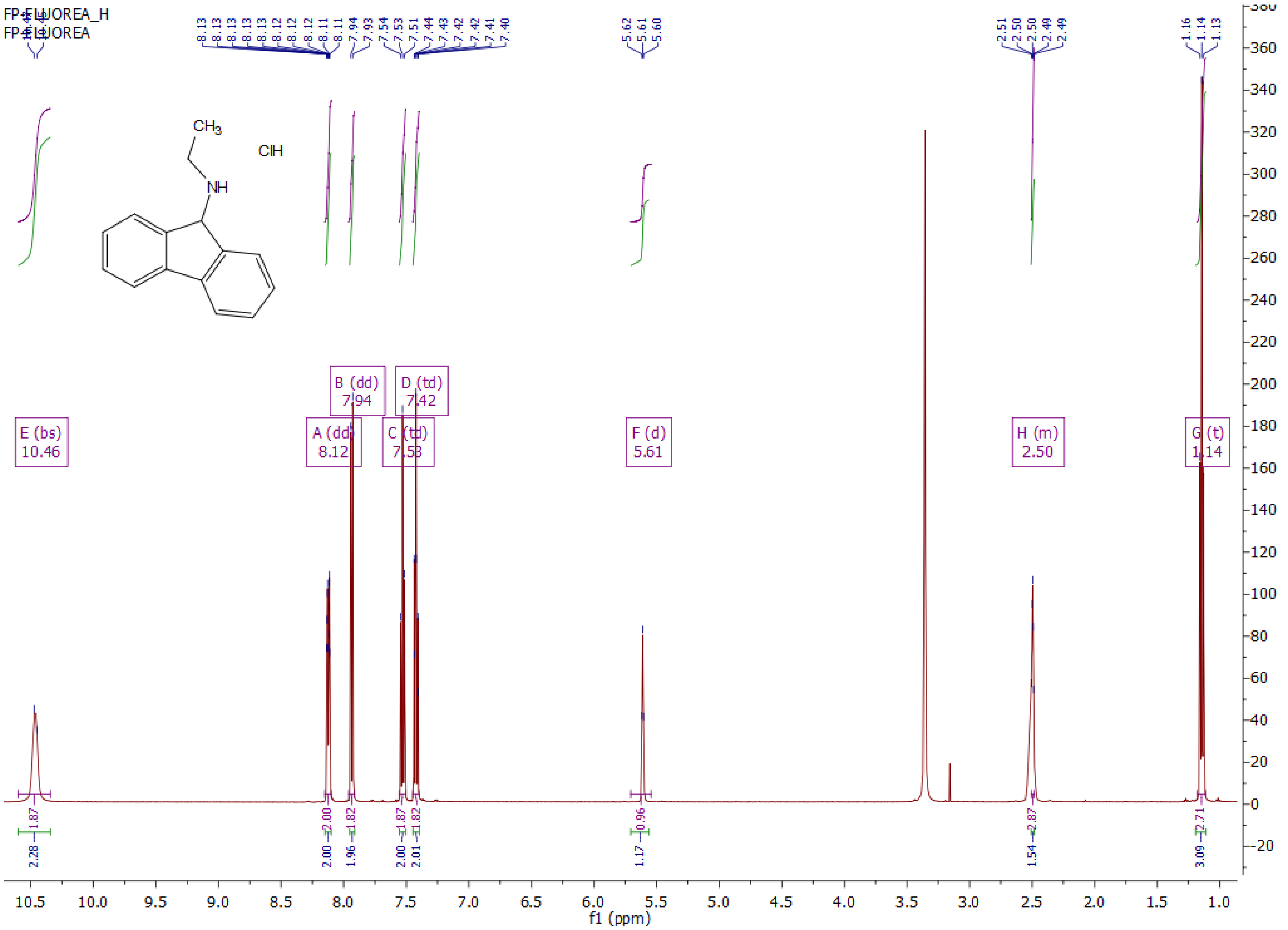

^{13}C NMR of *N*-ethyl-9*H*-fluoren-9-amine hydrochloride (3h):

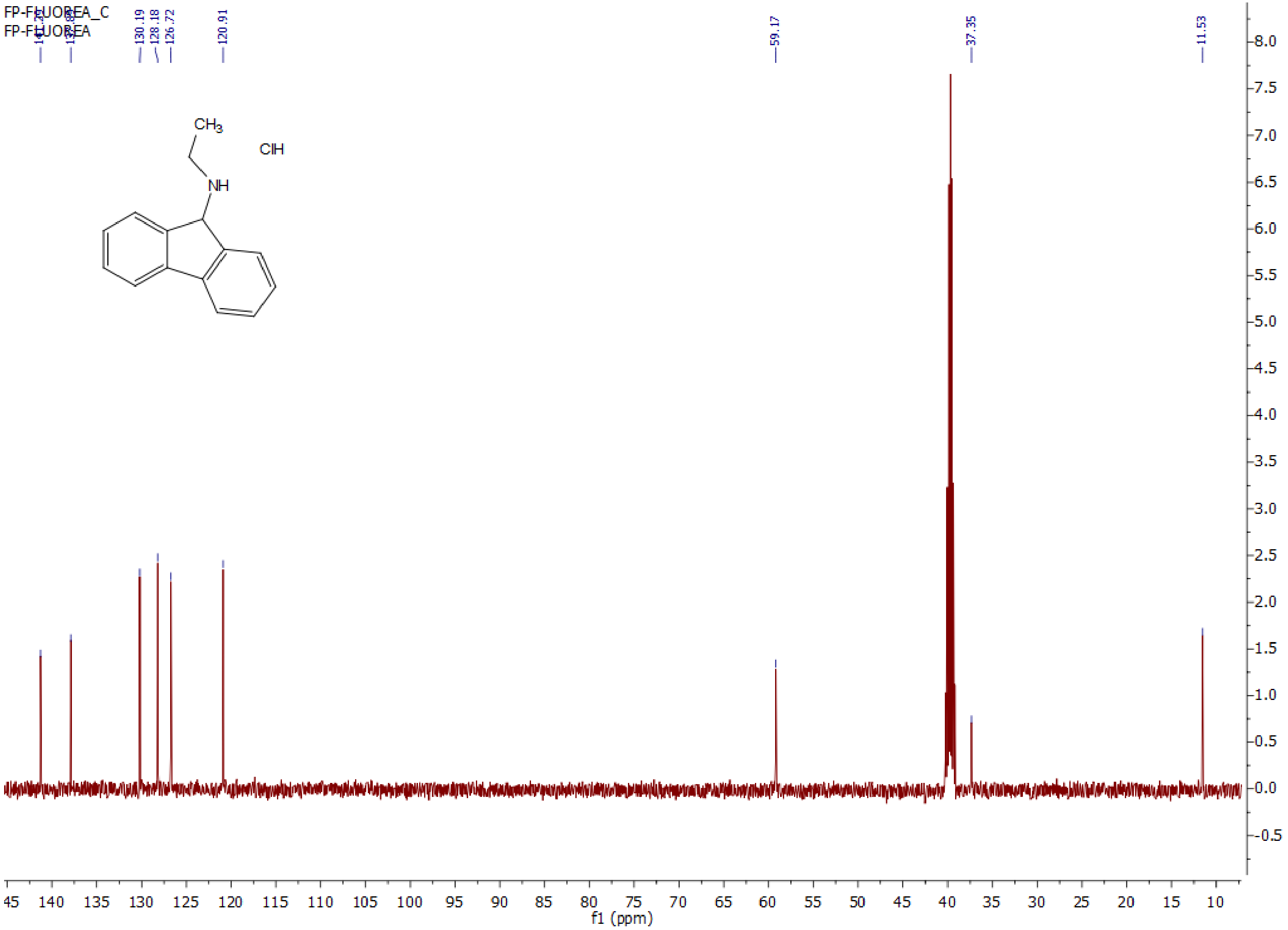

^{1}H NMR of *N,N*-diethyl-9*H*-fluoren-9-amine hydrochloride (3i):

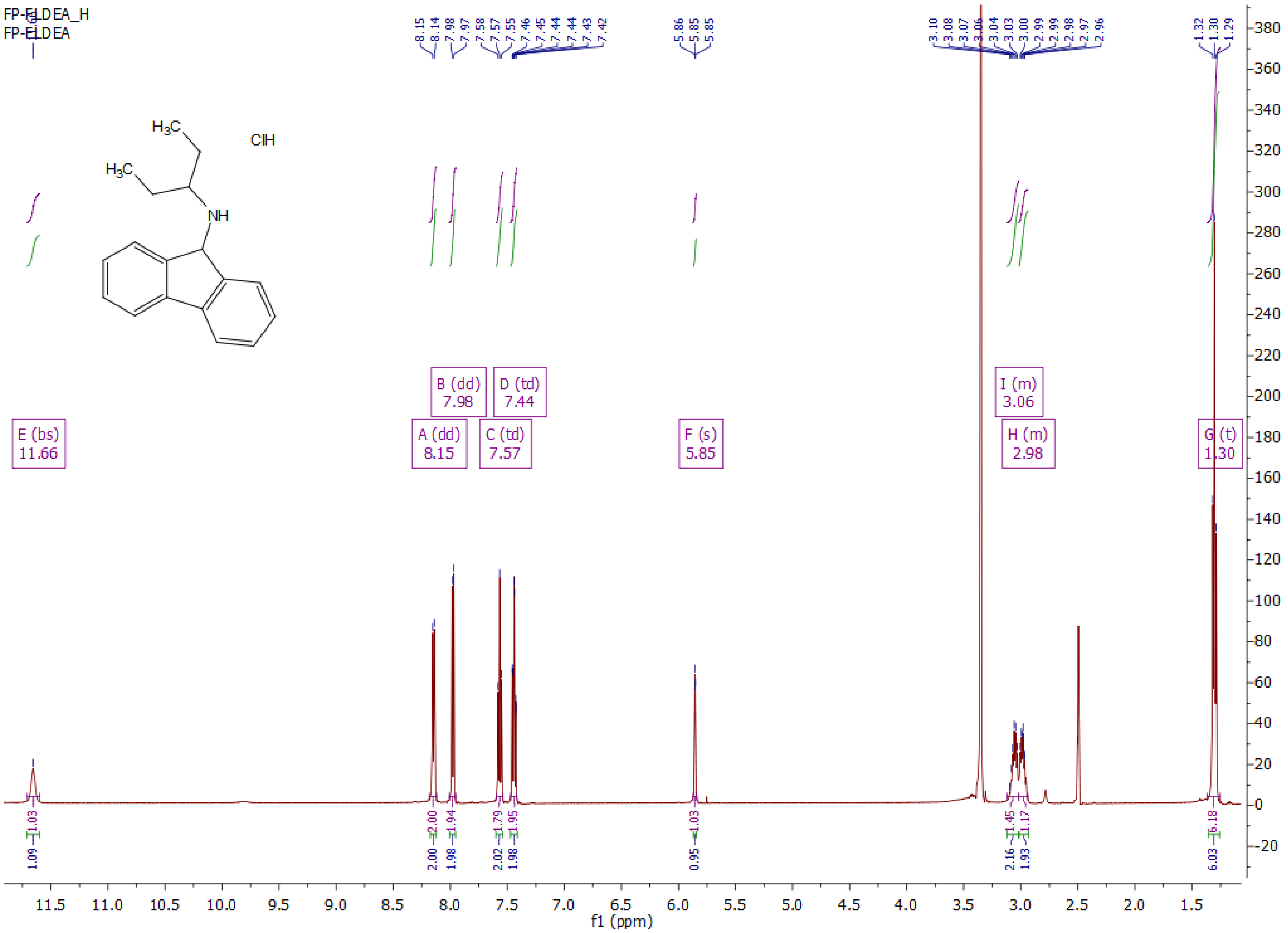

^{13}C NMR of *N,N*-diethyl-9*H*-fluoren-9-amine hydrochloride (3i):

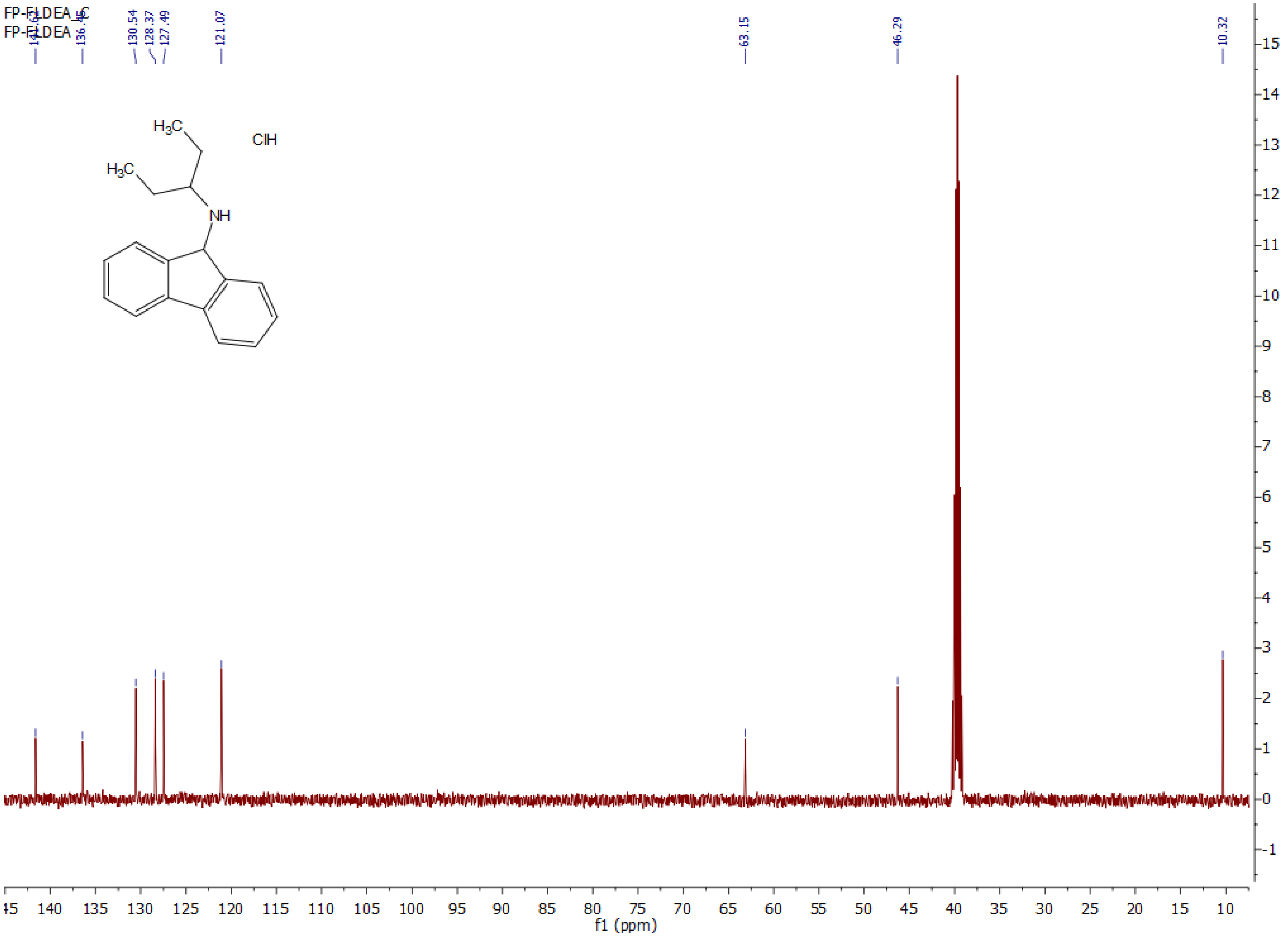

^{1}H NMR of 1-(9*H*-fluorene-9-yl)-4-methylpiperazine hydrochloride (3j):

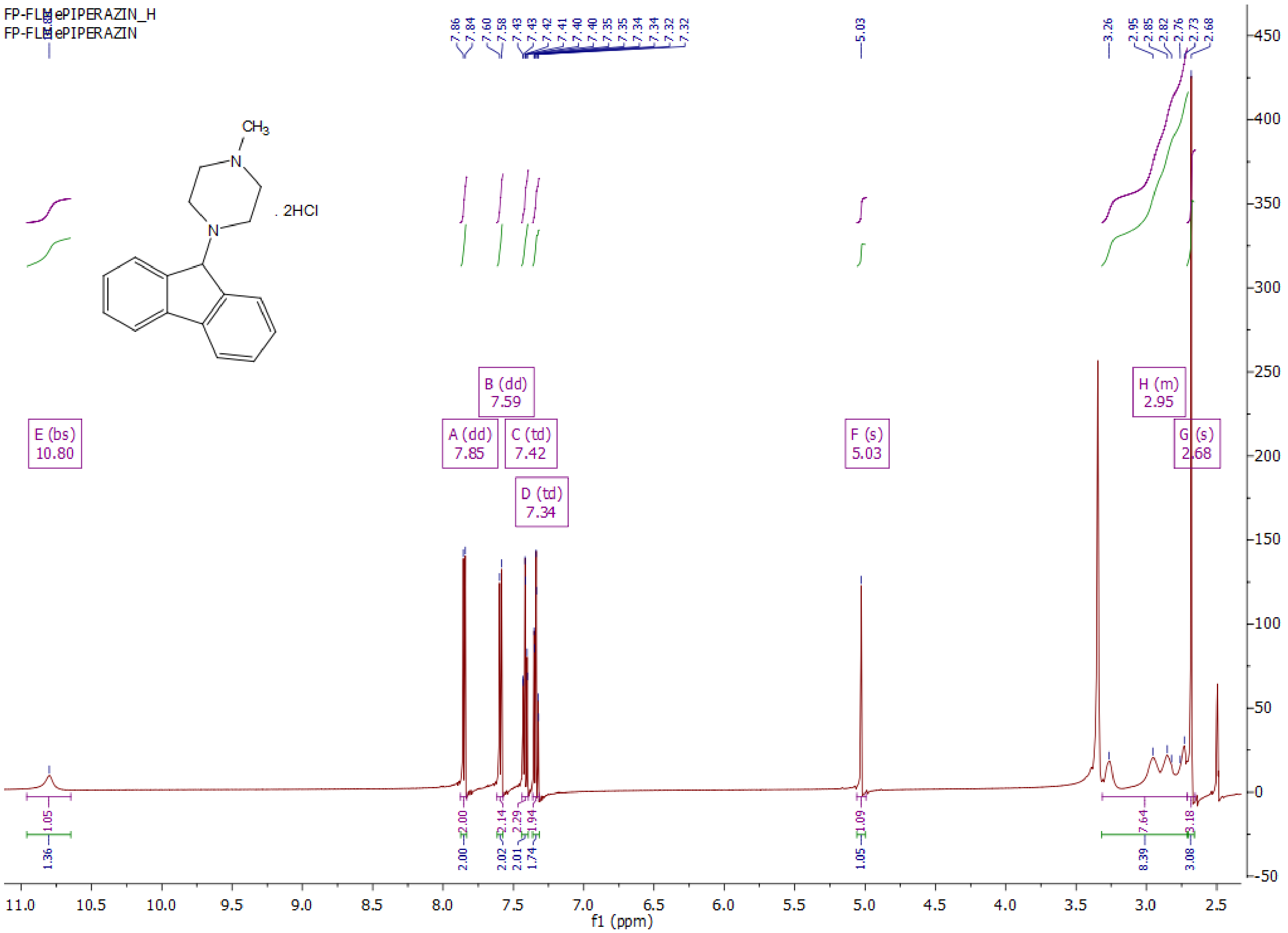

^{13}C NMR of 1-(9*H*-fluorene-9-yl)-4-methylpiperazine hydrochloride (3j):

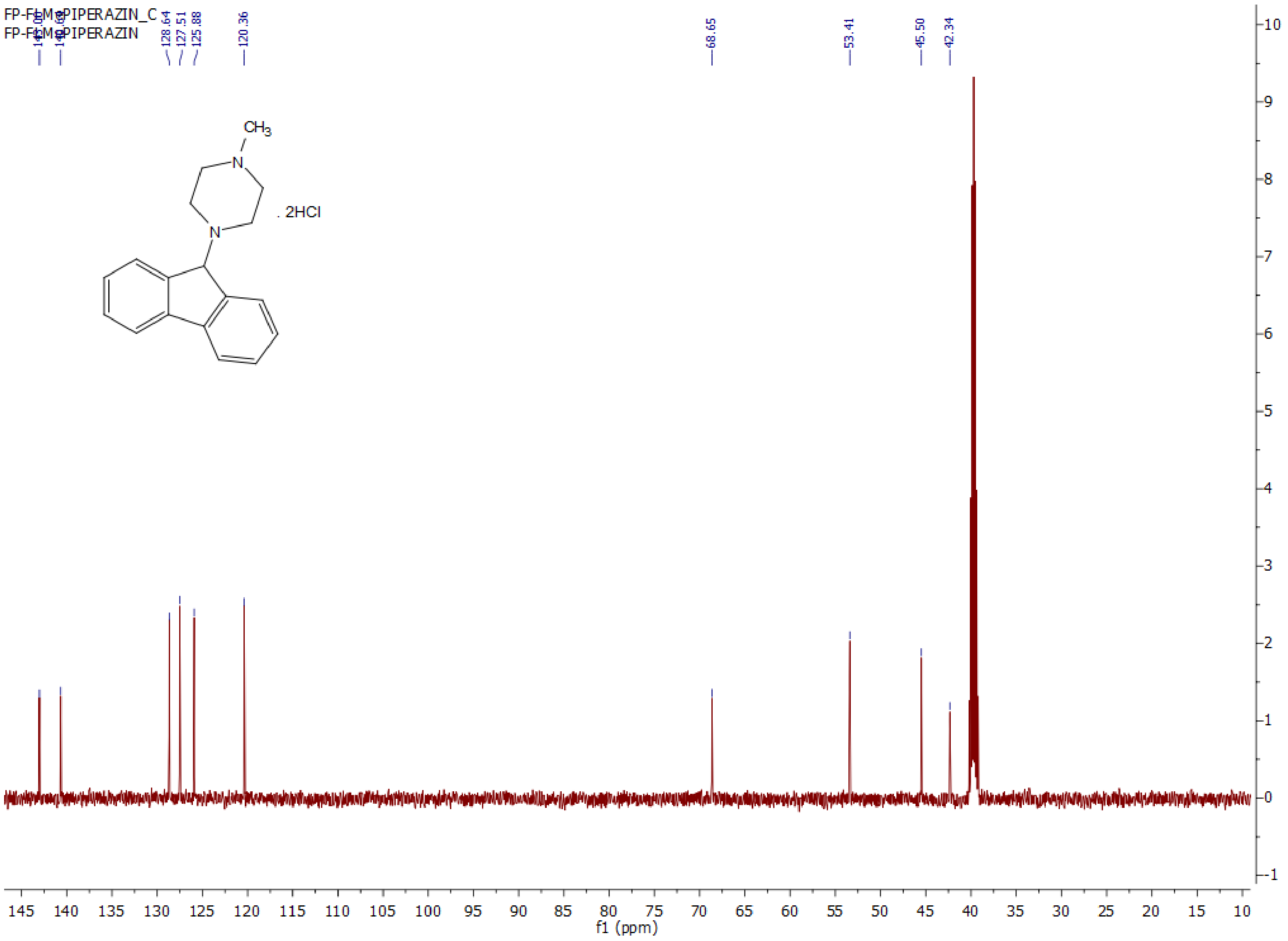

^{1}H NMR of 1-(9*H*-fluorene-9-yl)-4-ethylpiperazine hydrochloride (3k):

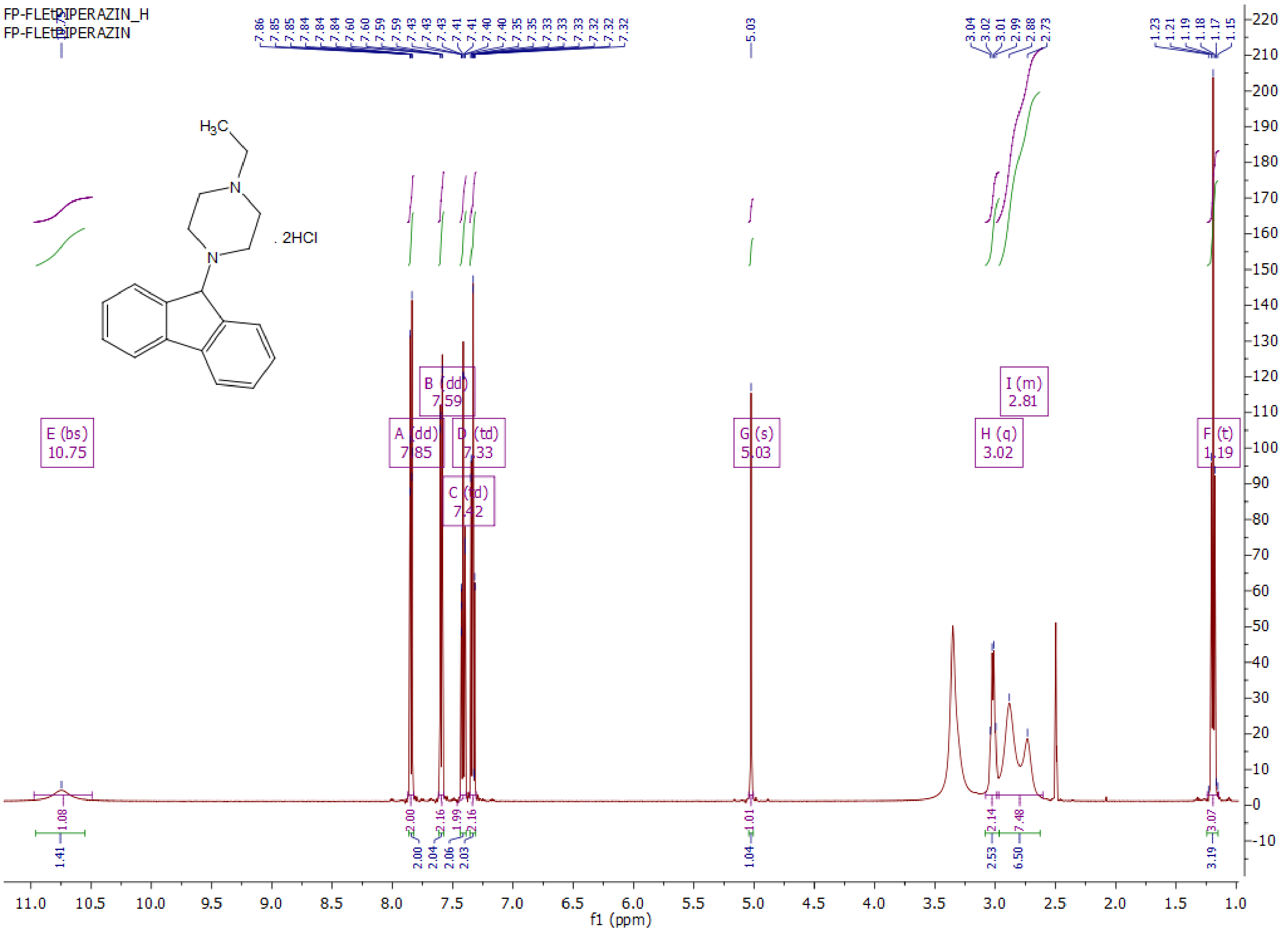

^{13}C NMR of 1-(9*H*-fluorene-9-yl)-4-ethylpiperazine hydrochloride (3k):

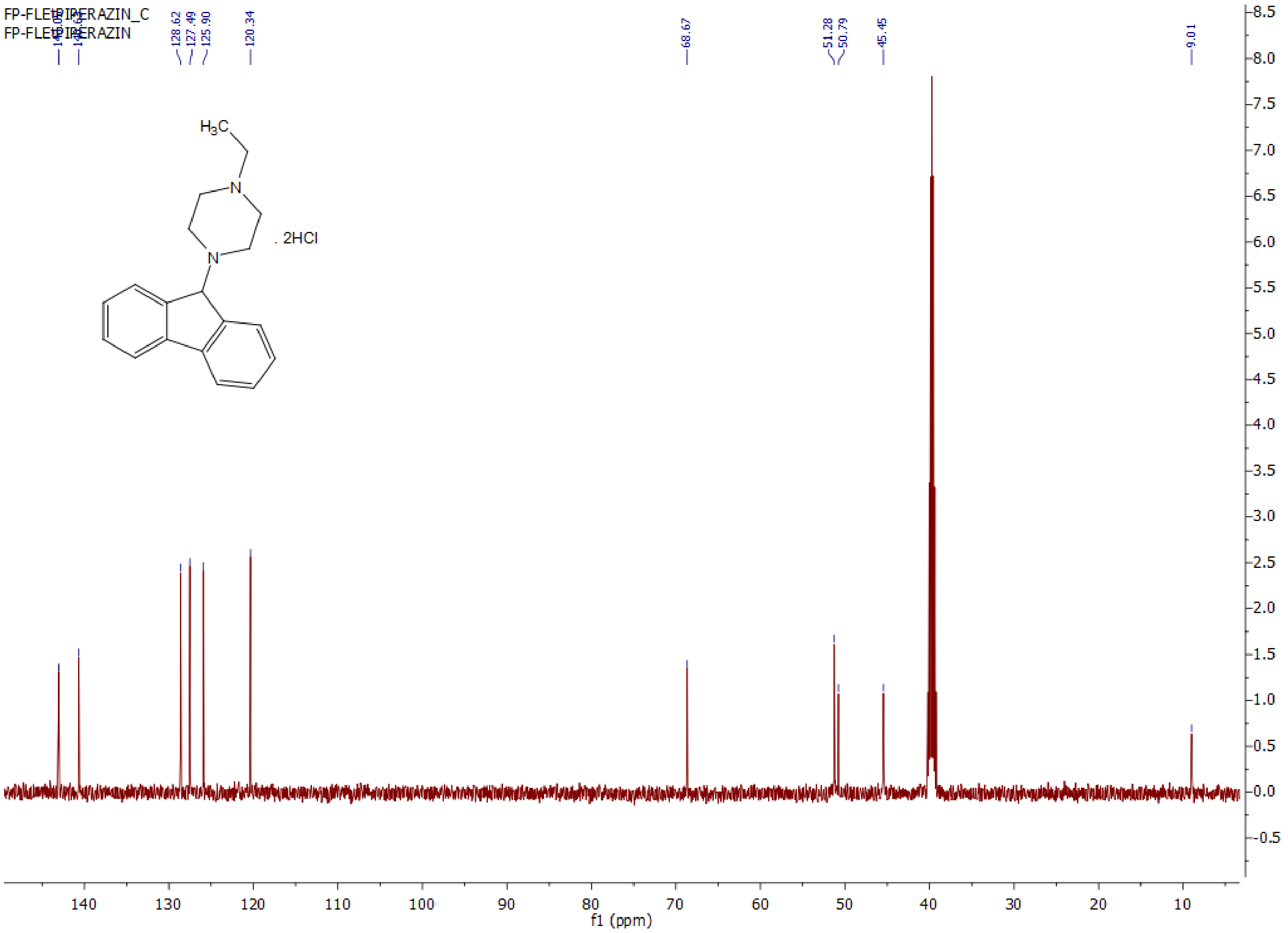

^{1}H NMR of 4-(9*H*-fluorene-9-yl)morpholine hydrochloride (3l):

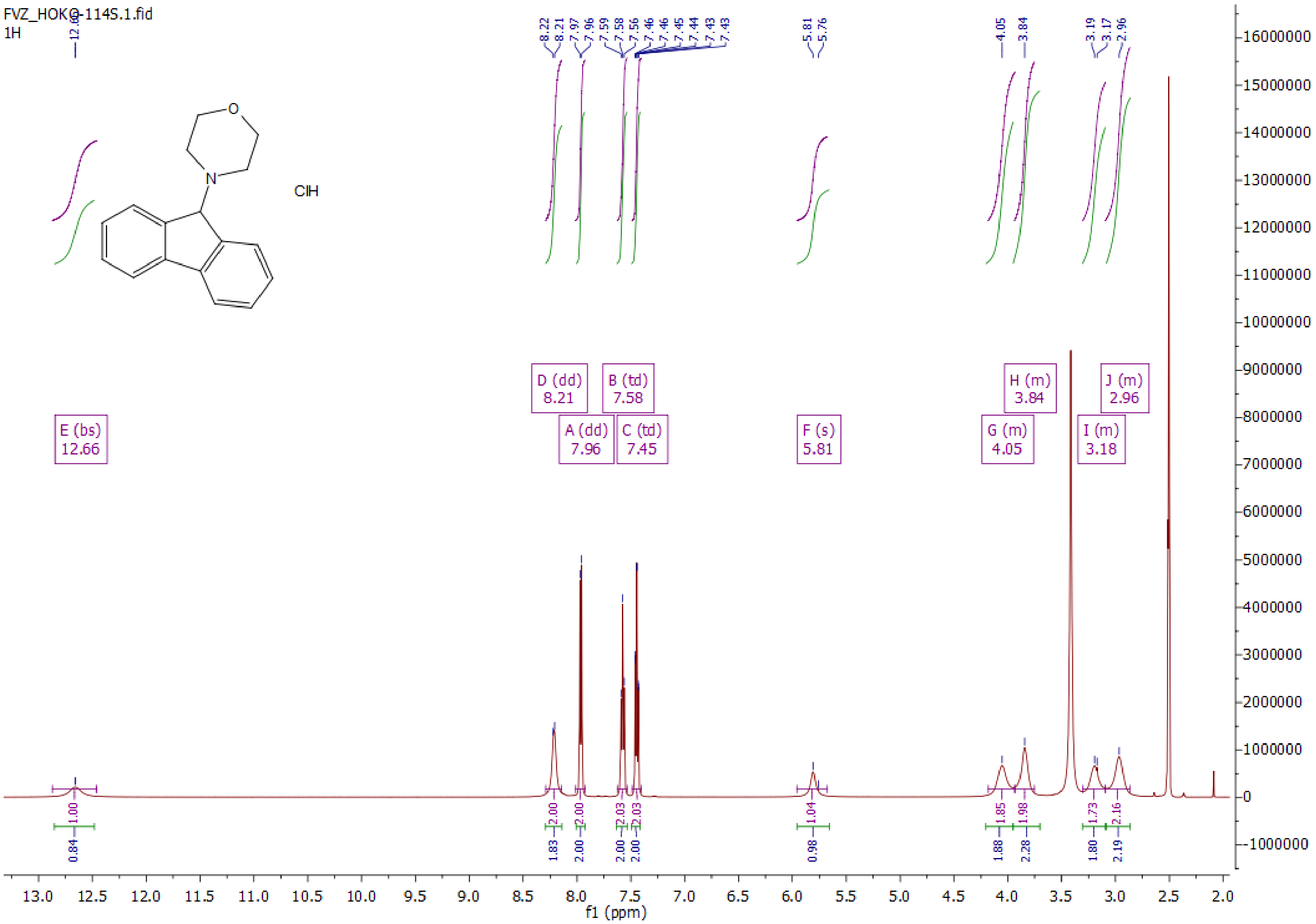

^{13}C NMR of 4-(9*H*-fluorene-9-yl)morpholine hydrochloride (3l):

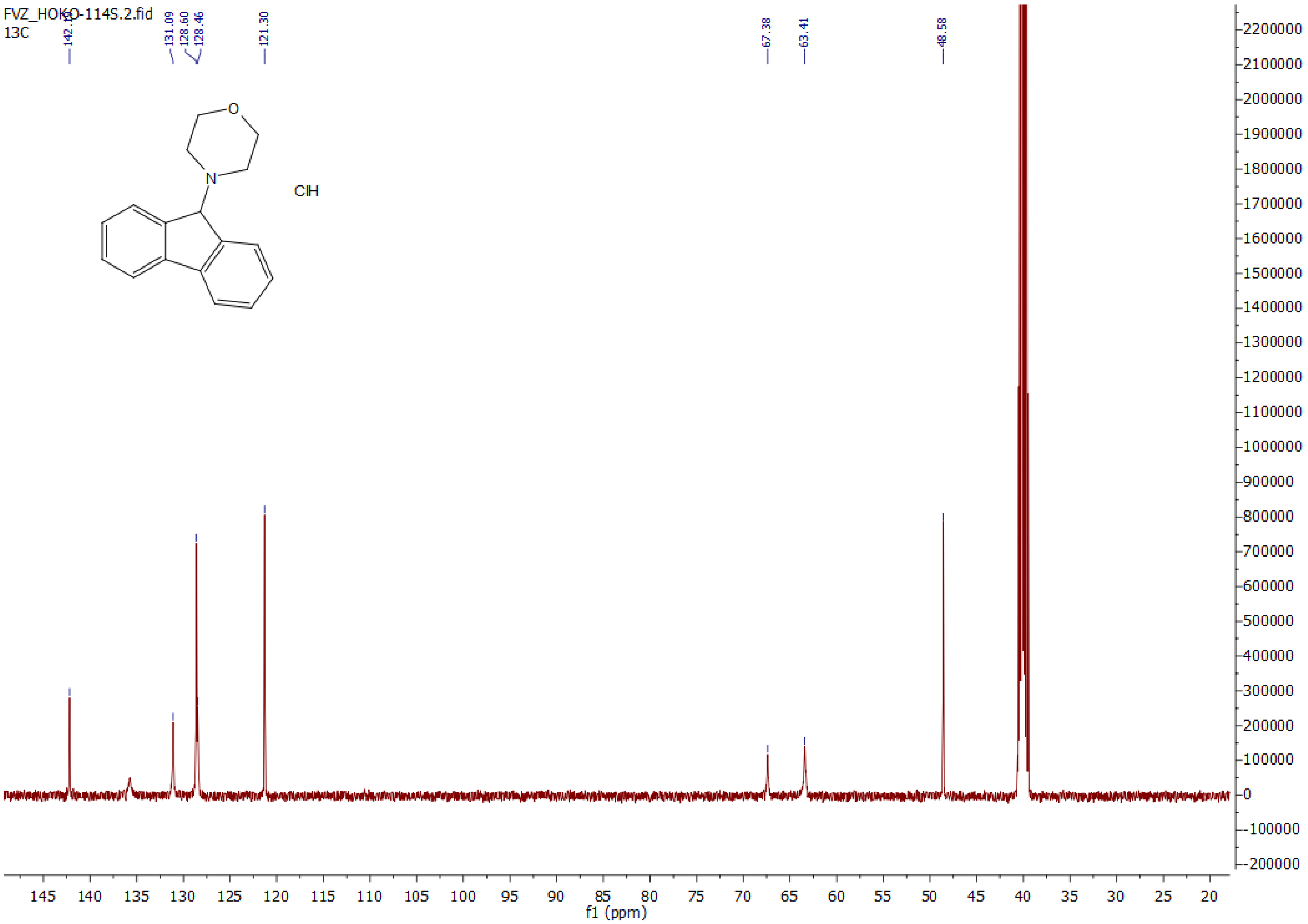

^{1}H NMR of *N*-butyl-9*H*-fluoren-9-amine hydrochloride (3m):

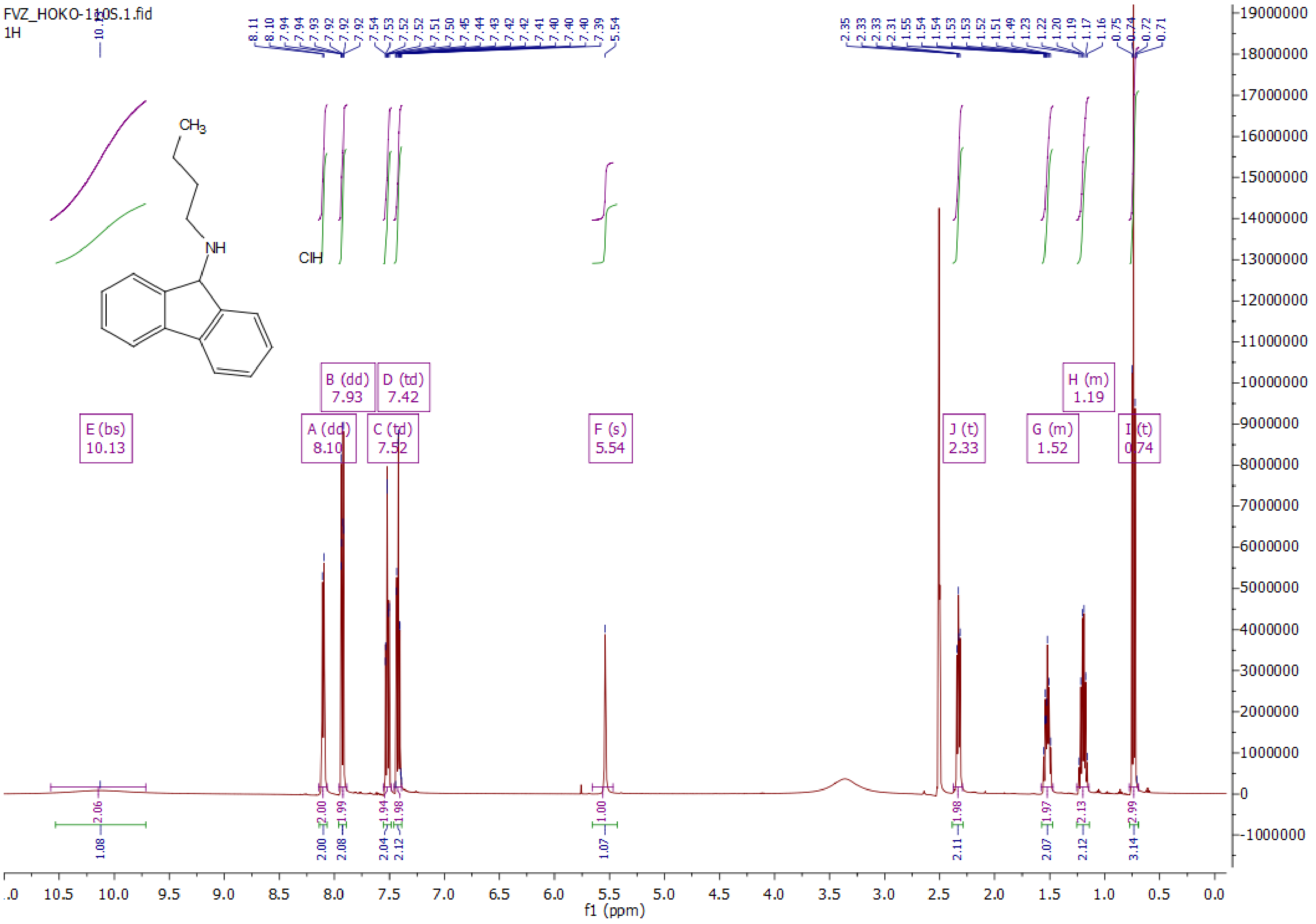

^{13}C NMR of *N*-butyl-9*H*-fluoren-9-amine hydrochloride (3m):

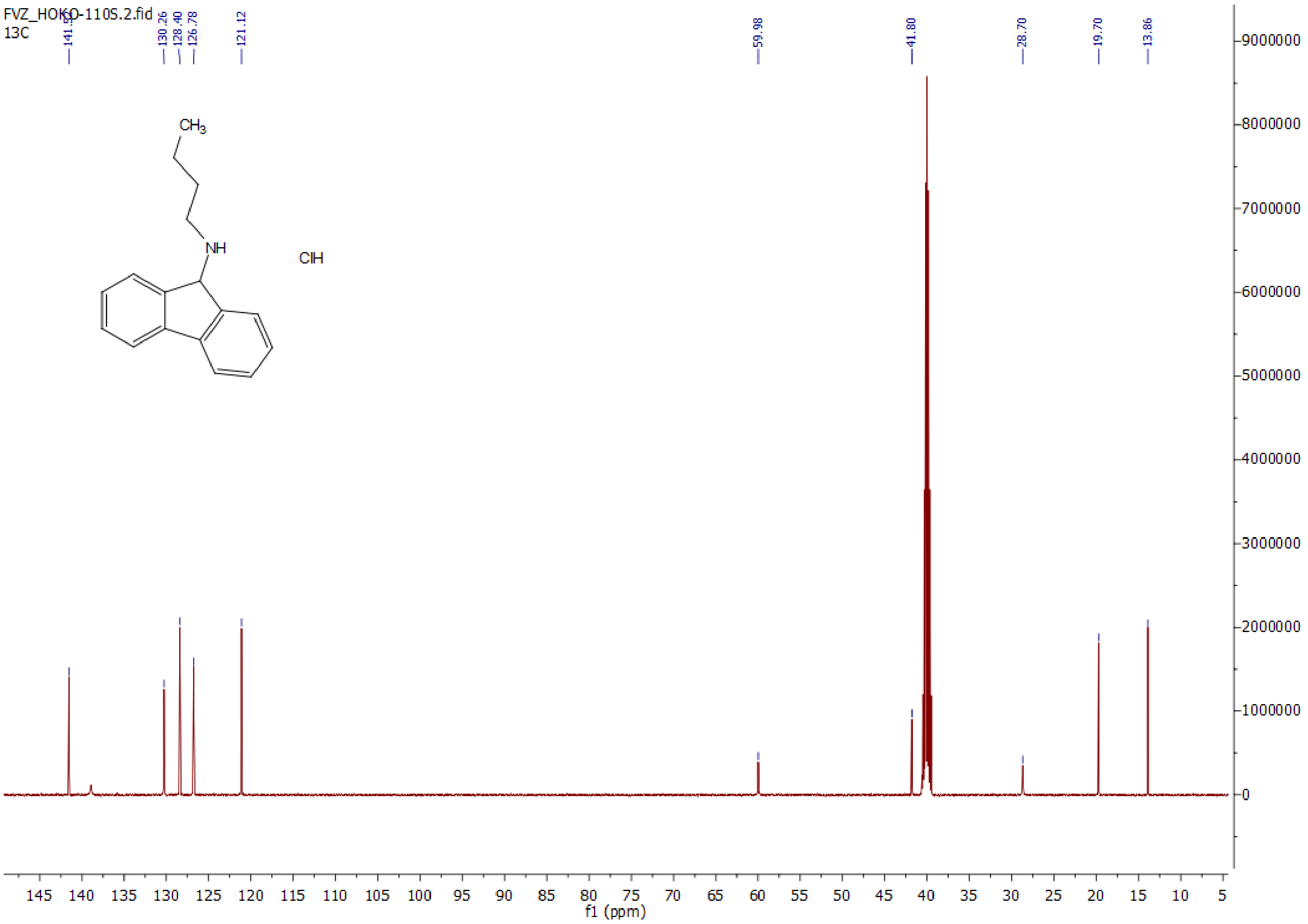

^{1}H NMR of *N*-(2-methylpropyl)-9*H*-fluoren-9-amine hydrochloride (3n):

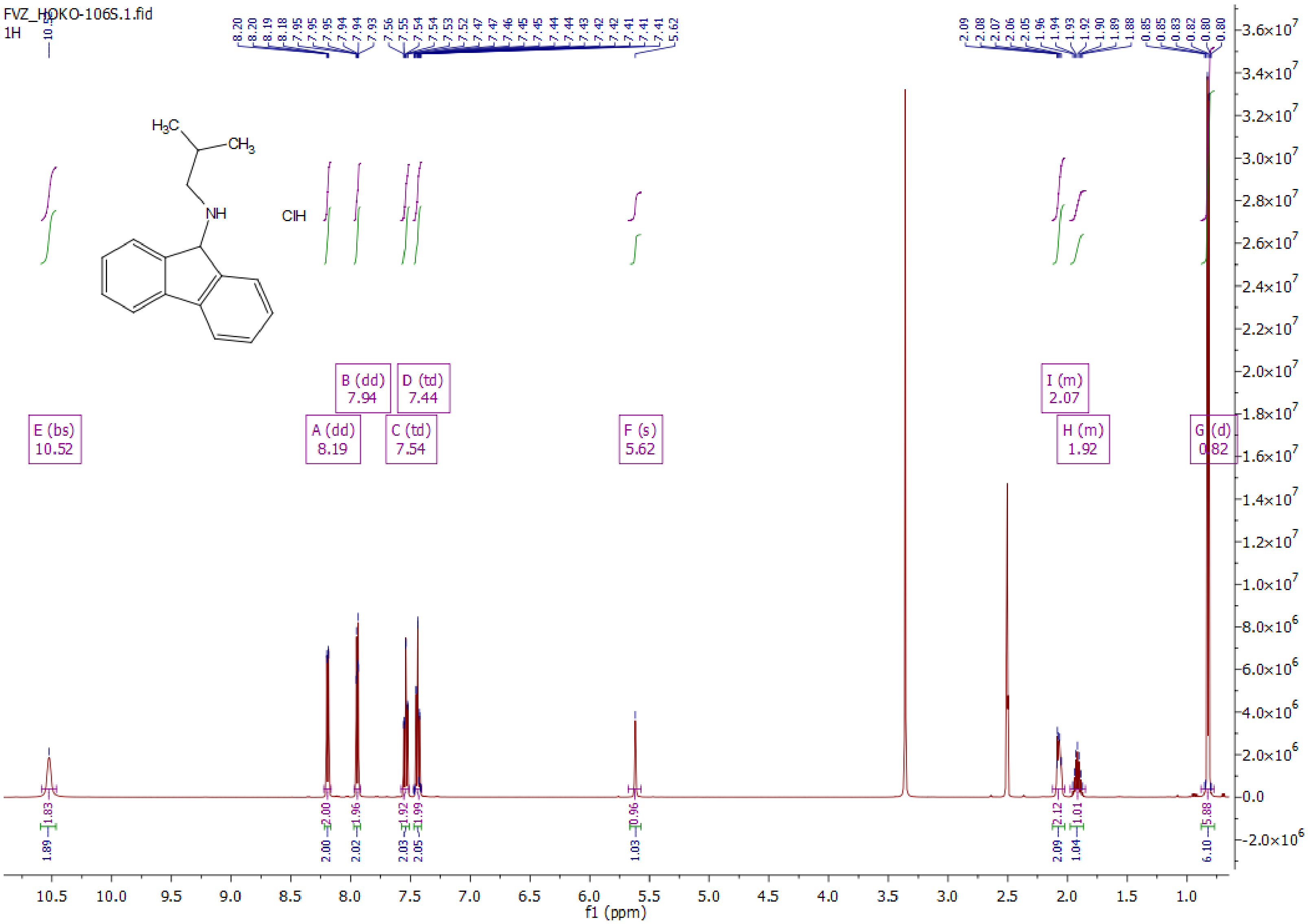

^{13}C NMR of *N*-(2-methylpropyl)-9*H*-fluoren-9-amine hydrochloride (3n):

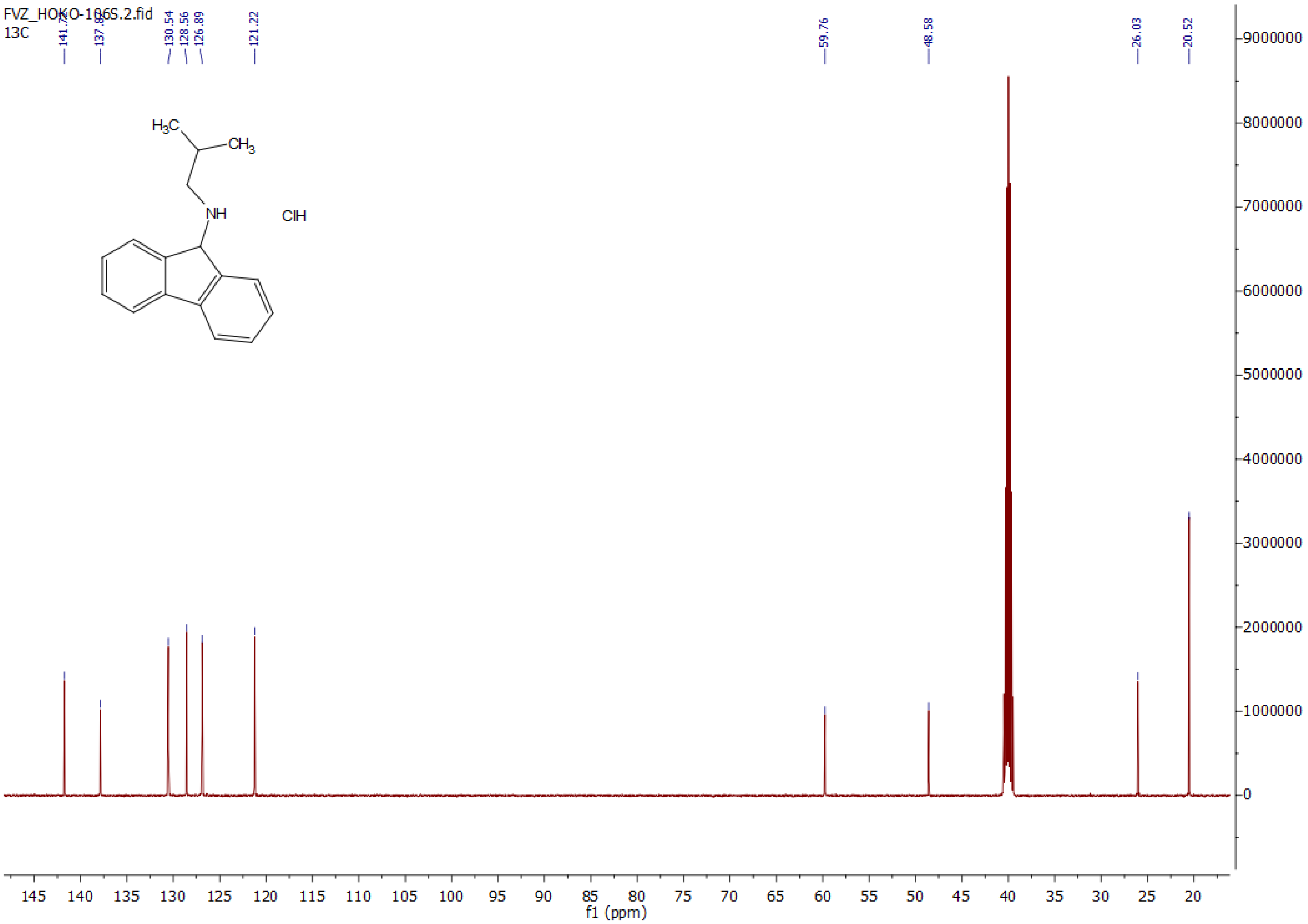

^{1}H NMR of 1-(9*H*-fluoren-9-yl)pyrrolidine hydrochloride (3o):

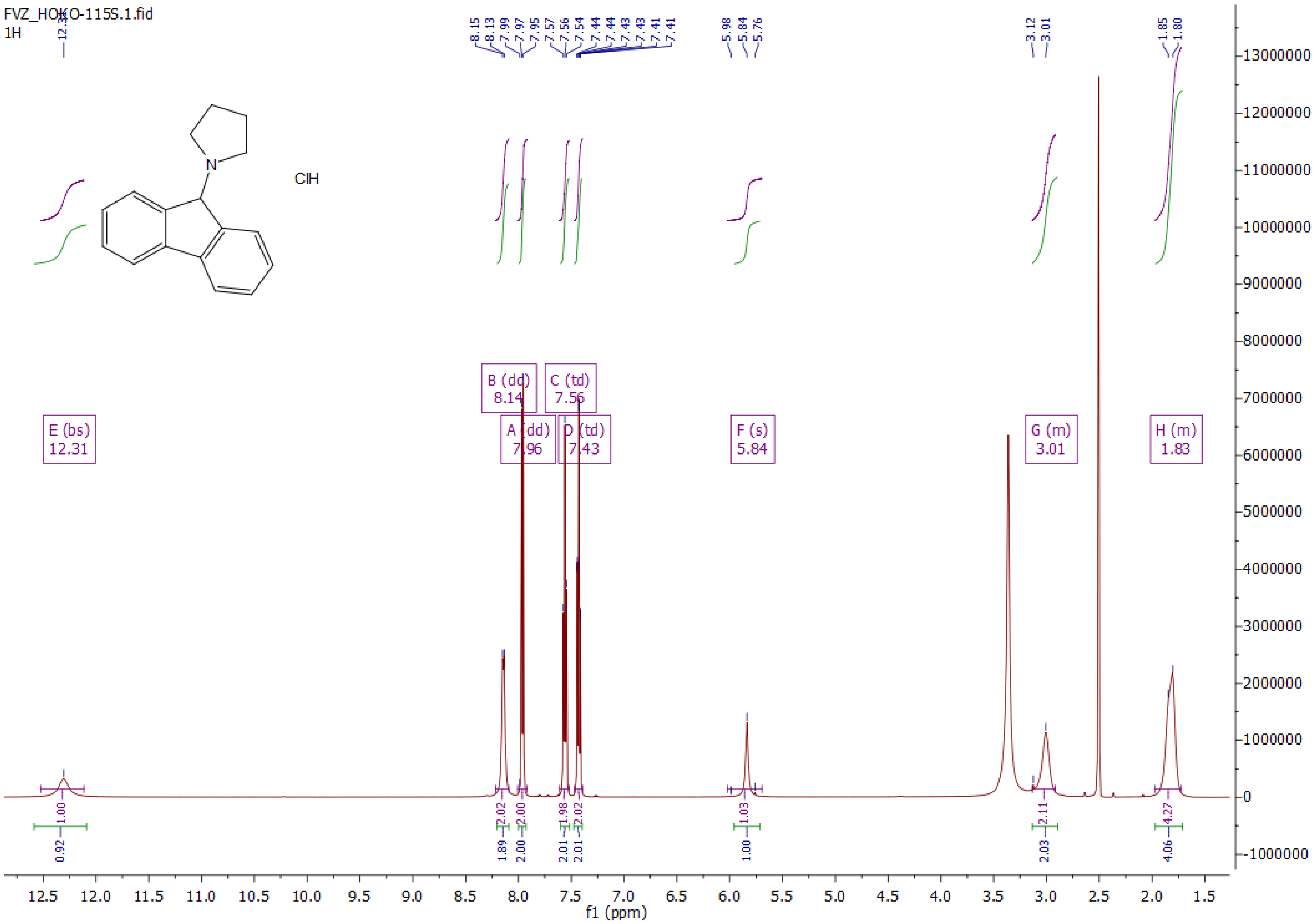

^{13}C NMR of 1-(9*H*-fluoren-9-yl)pyrrolidine hydrochloride (3o):

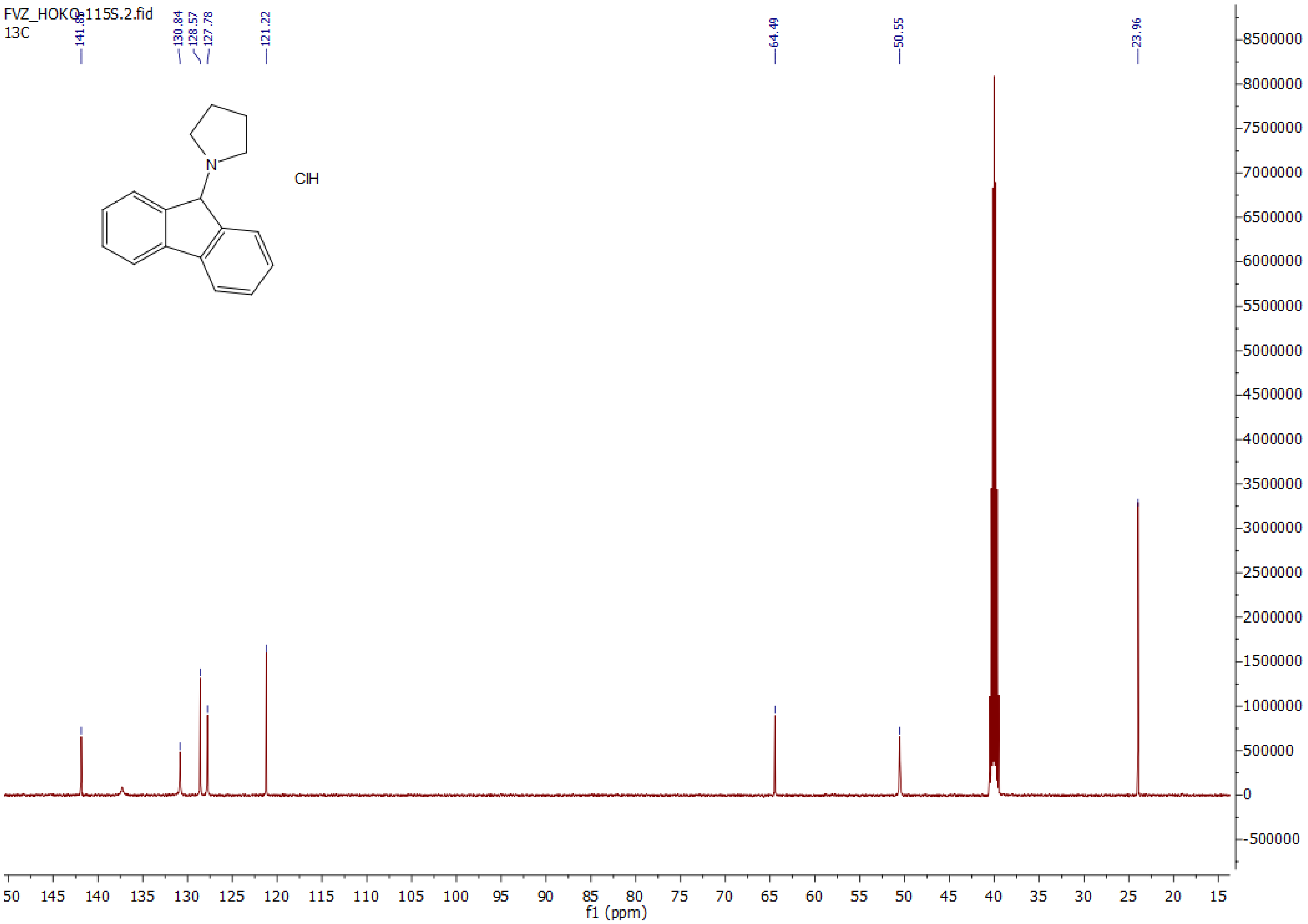

4. HPLC chromatograms and MS spectra of final compounds

HPLC chromatogram (UV 254 nm) of *N*-propan-2-yl-9*H*-fluoren-9-amine hydrochloride (3a)

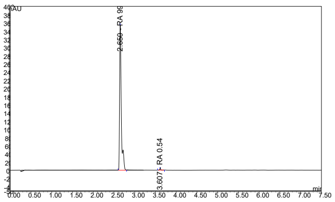

MS spectrum of *N*-propan-2-yl-9*H*-fluoren-9-amine hydrochloride (3a) at R_t: 2.66 min:

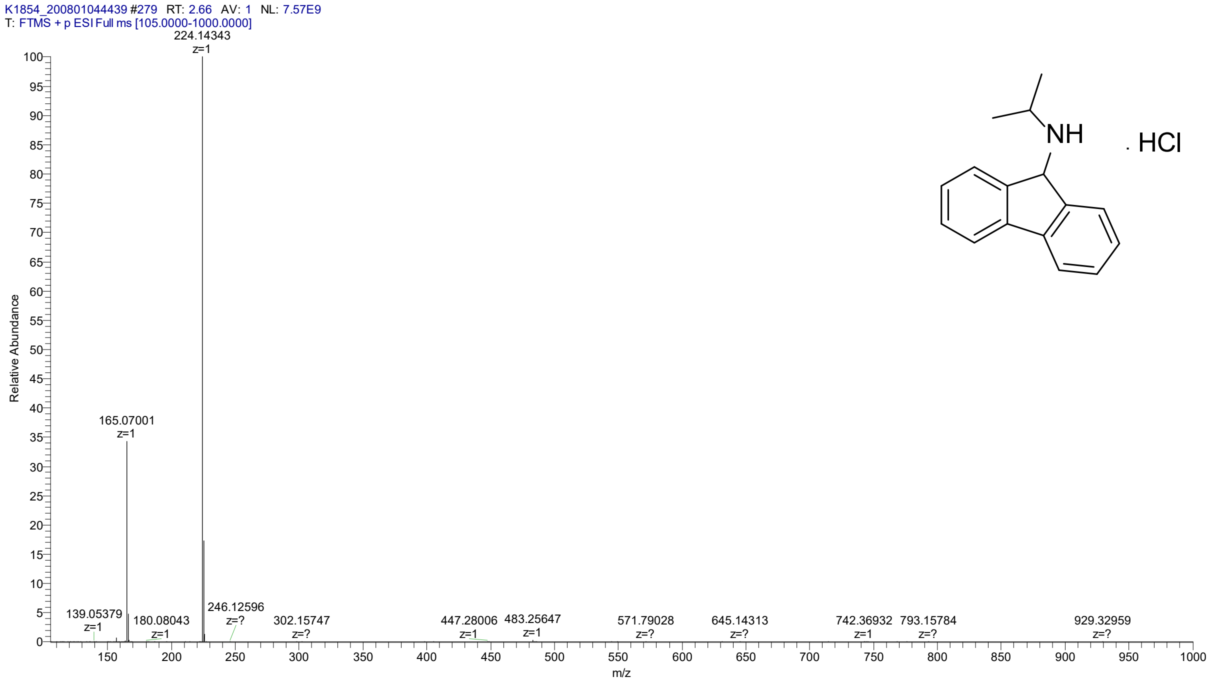

HPLC chromatogram (UV 254 nm) of *N*-methyl-9*H*-fluoren-9-amine hydrochloride (3b):

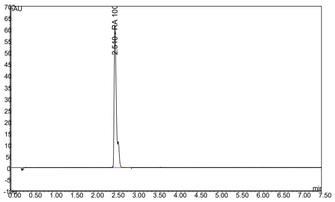

MS spectrum of *N*-methyl-9*H*-fluoren-9-amine hydrochloride (3b) at R_t: 2.53 min:

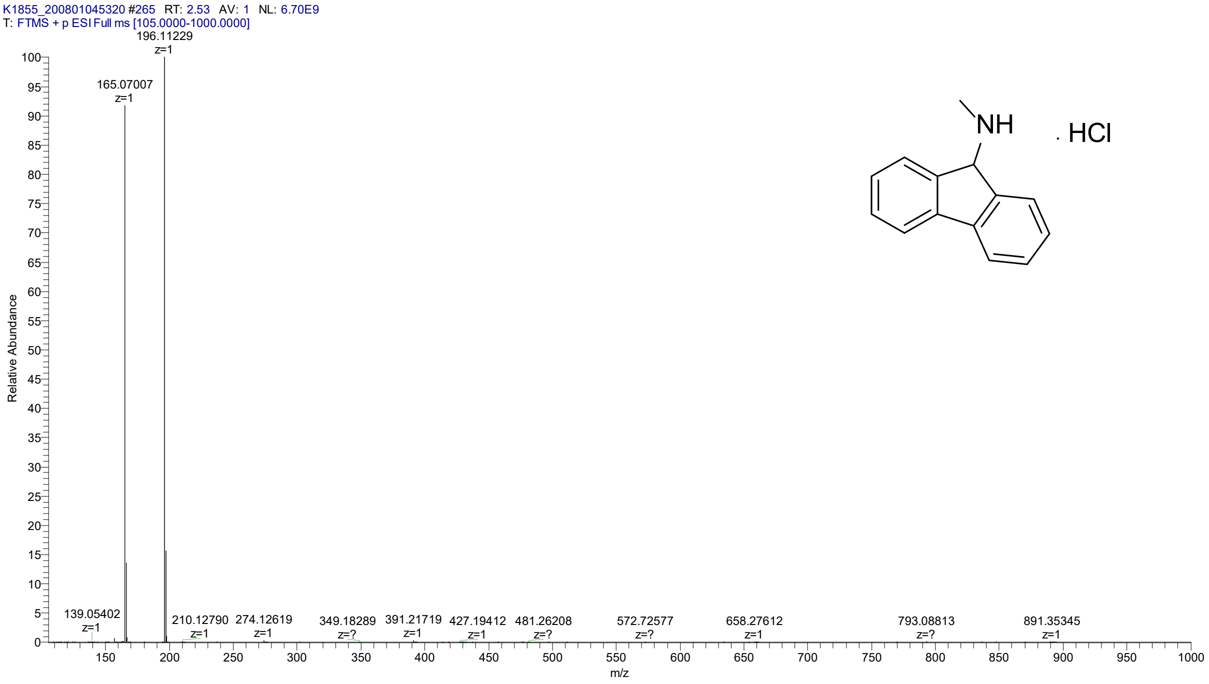

HPLC chromatogram (UV 254 nm) of *N*-cyclohexyl-9*H*-fluoren-9-amine hydrochloride (3c):

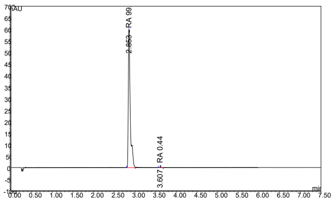

MS spectrum of *N*-cyclohexyl-9*H*-fluoren-9-amine hydrochloride (3c) at R_t: 2.86 min:

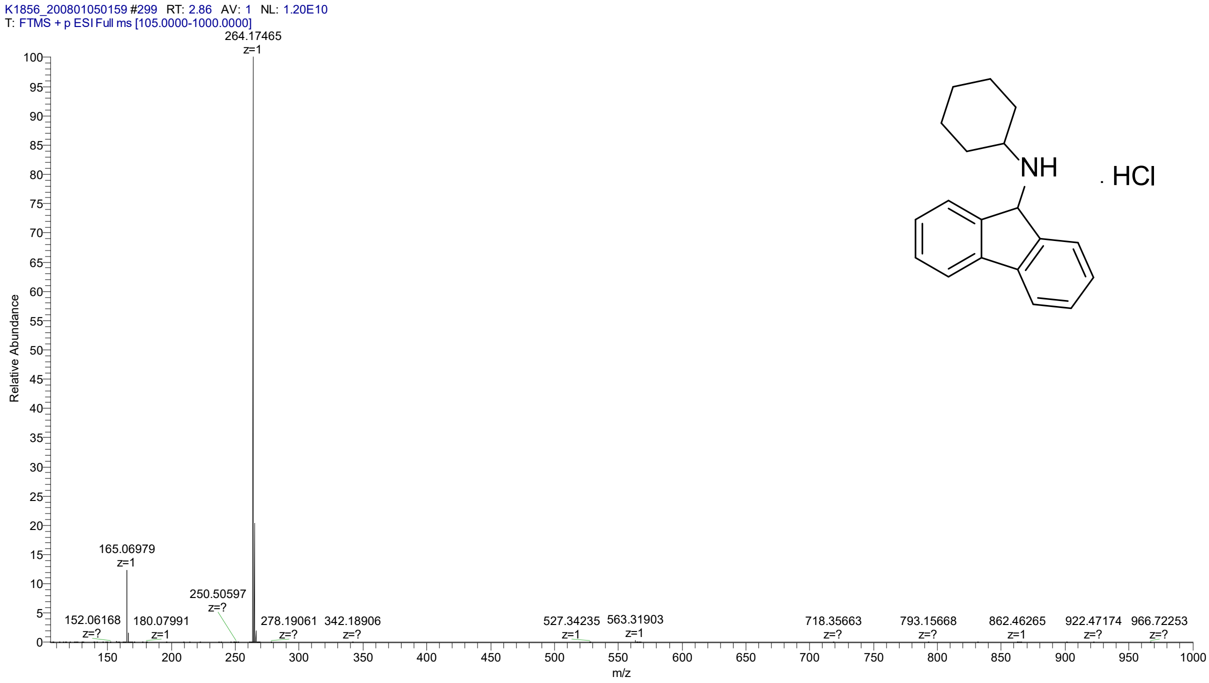

HPLC chromatogram (UV 254 nm) of *N*-cyclopropyl-9*H*-fluoren-9-amine hydrochloride (3d):

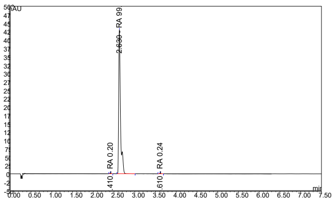

MS spectrum of *N*-cyclopropyl-9*H*-fluoren-9-amine hydrochloride (3d) at R_t: 2.64 min:

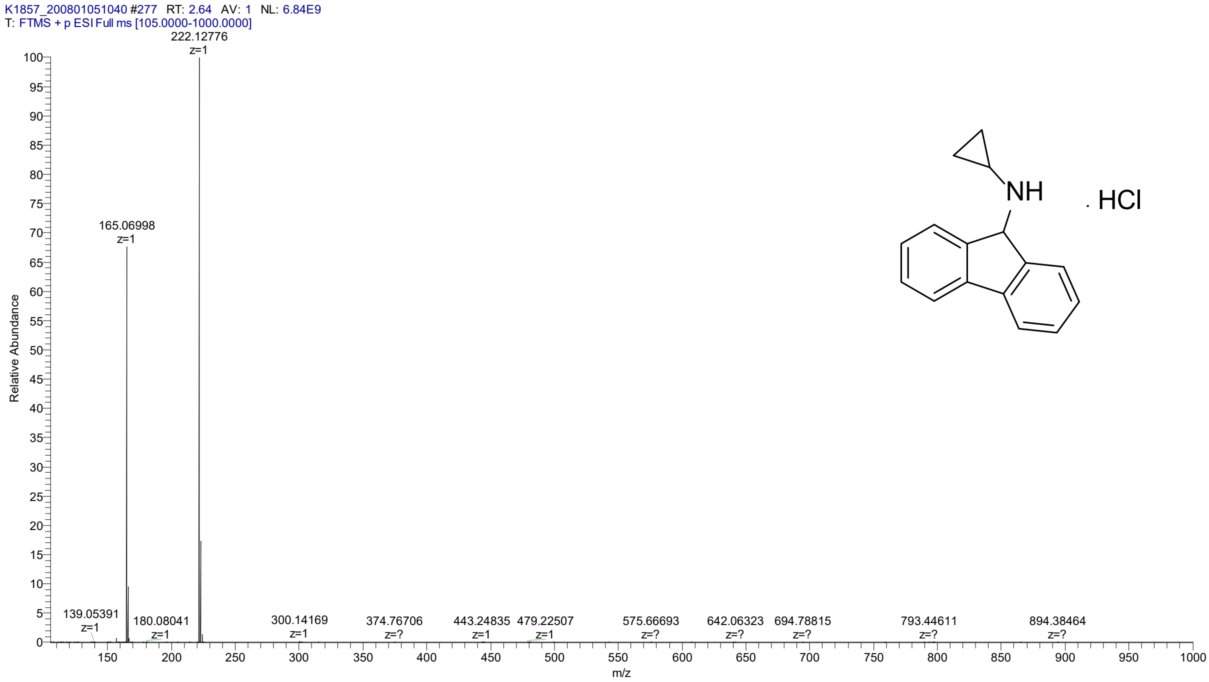

HPLC chromatogram (UV 254 nm) of *N*-cyclobutyl-9*H*-fluoren-9-amine hydrochloride (3e):

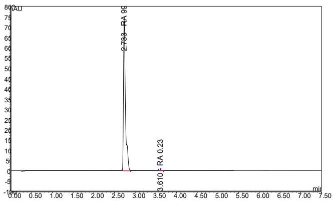

MS spectrum of *N*-cyclobutyl-9*H*-fluoren-9-amine hydrochloride (3e) at R_t: 2.77 min:

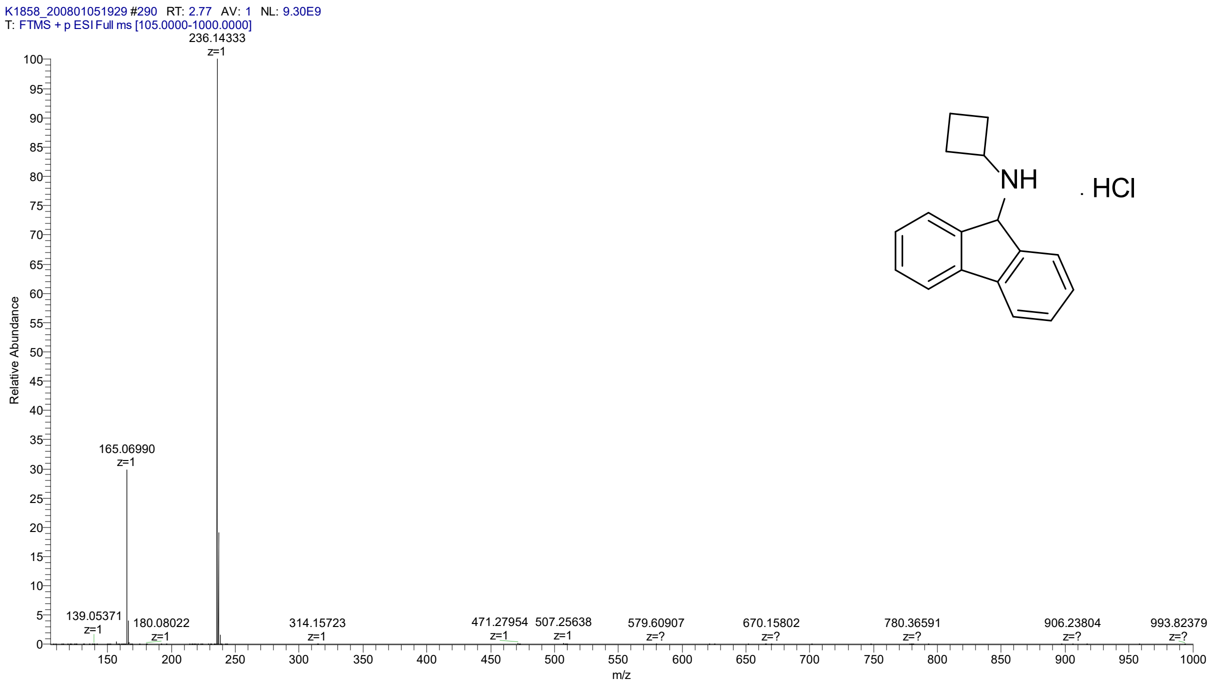

HPLC chromatogram (UV 254 nm) of 1-(9*H*-fluoren-9-yl)piperidine hydrochloride (3f):

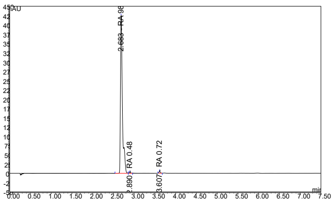

MS spectrum of 1-(9*H*-fluoren-9-yl)piperidine hydrochloride (3f) at R_t: 2.70 min:

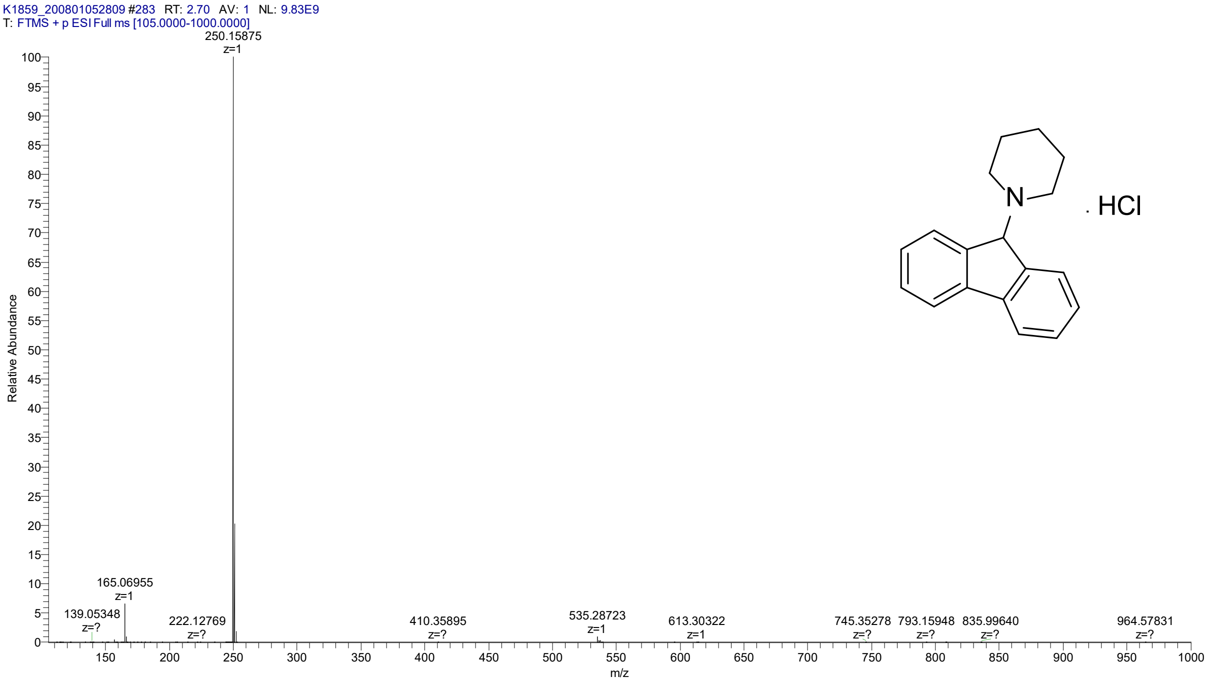

HPLC chromatogram (UV 254 nm) of *N*-(2-methoxyethyl)-9*H*-fluoren-9-amine hydrochloride (3g):

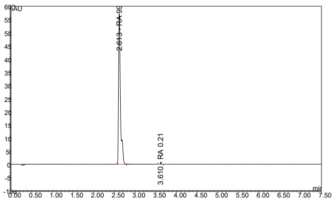

MS spectrum of *N*-(2-methoxyethyl)-9*H*-fluoren-9-amine hydrochloride (3g) at R_t: 2.62 min:

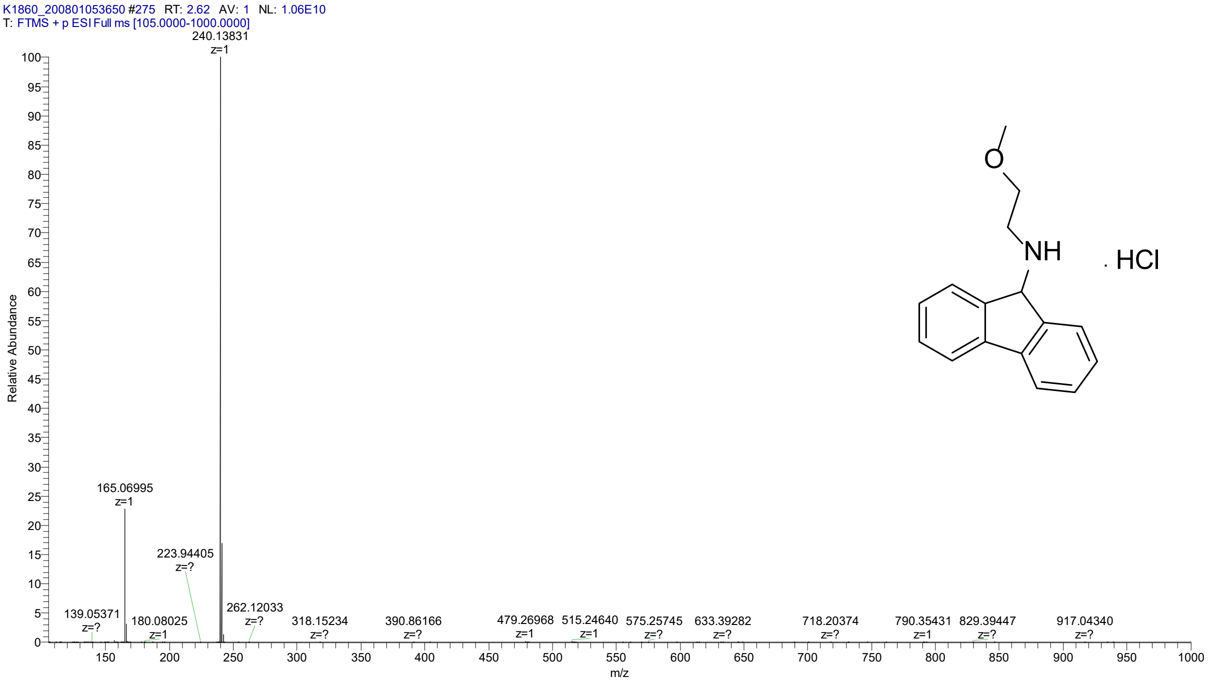

HPLC chromatogram (UV 254 nm) of *N*-ethyl-9*H*-fluoren-9-amine hydrochloride (3h):

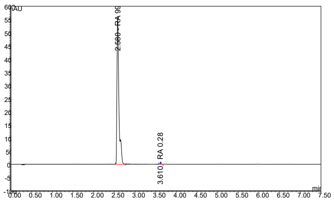

MS spectrum of *N*-ethyl-9*H*-fluoren-9-amine hydrochloride (3h) at R_t: 2.58 min:

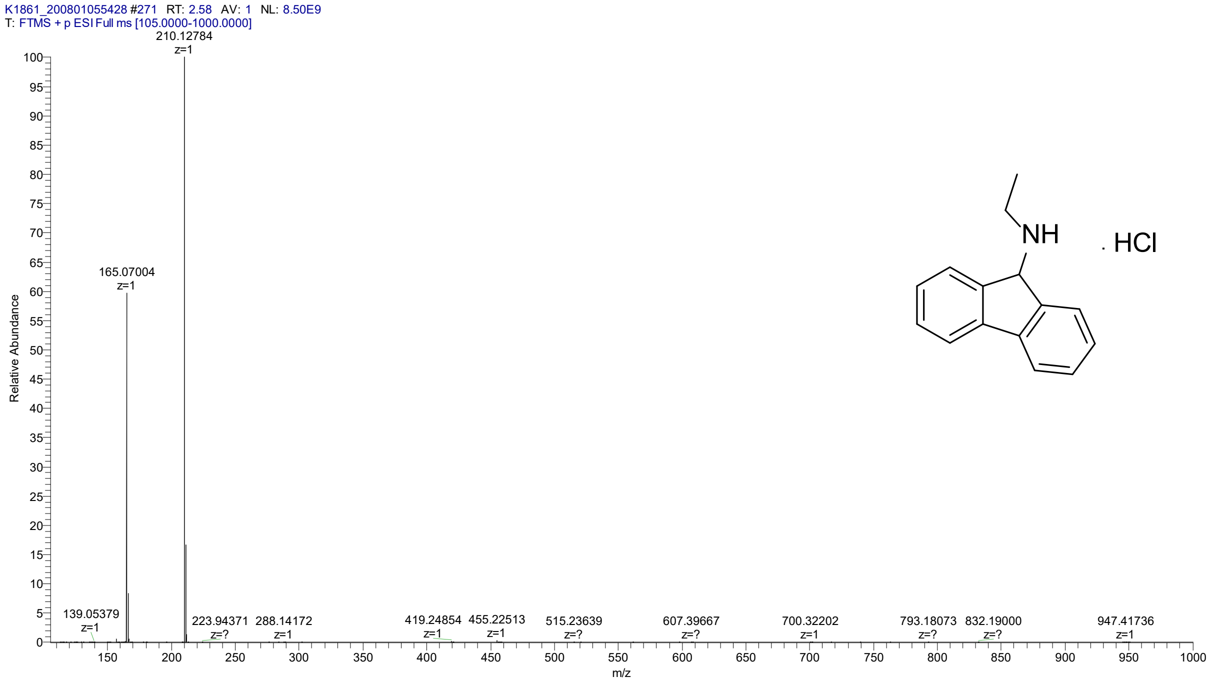

HPLC chromatogram (UV 254 nm) of *N*,*N*-diethyl-9*H*-fluoren-9-amine hydrochloride (3i):

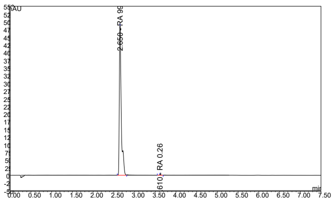

MS spectrum of *N*,*N*-diethyl-9*H*-fluoren-9-amine hydrochloride (3i) at R_t: 2.65 min:

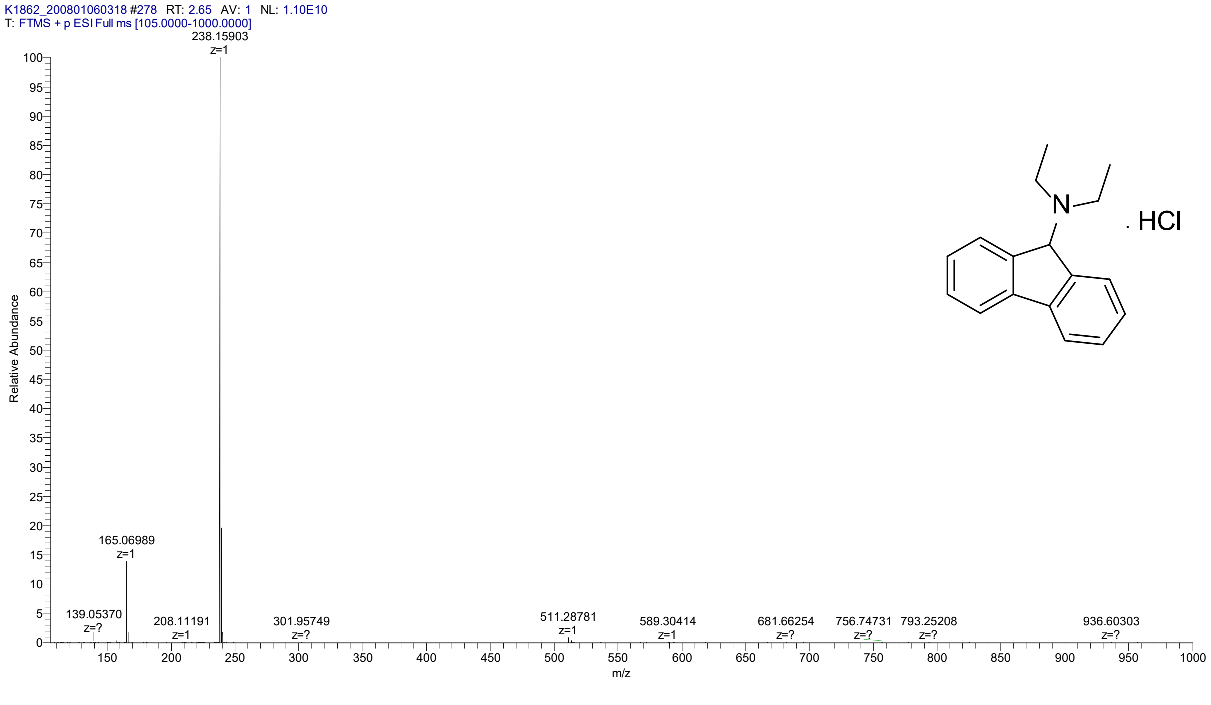

HPLC chromatogram (UV 254 nm) of 1-(9*H*-fluorene-9-yl)-4-methylpiperazine hydrochloride (3j):

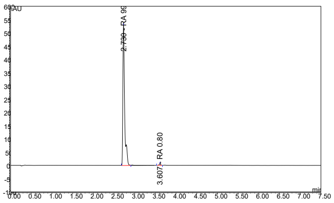

MS spectrum of 1-(9*H*-fluorene-9-yl)-4-methylpiperazine hydrochloride (3j) at R_t: 2.75 min:

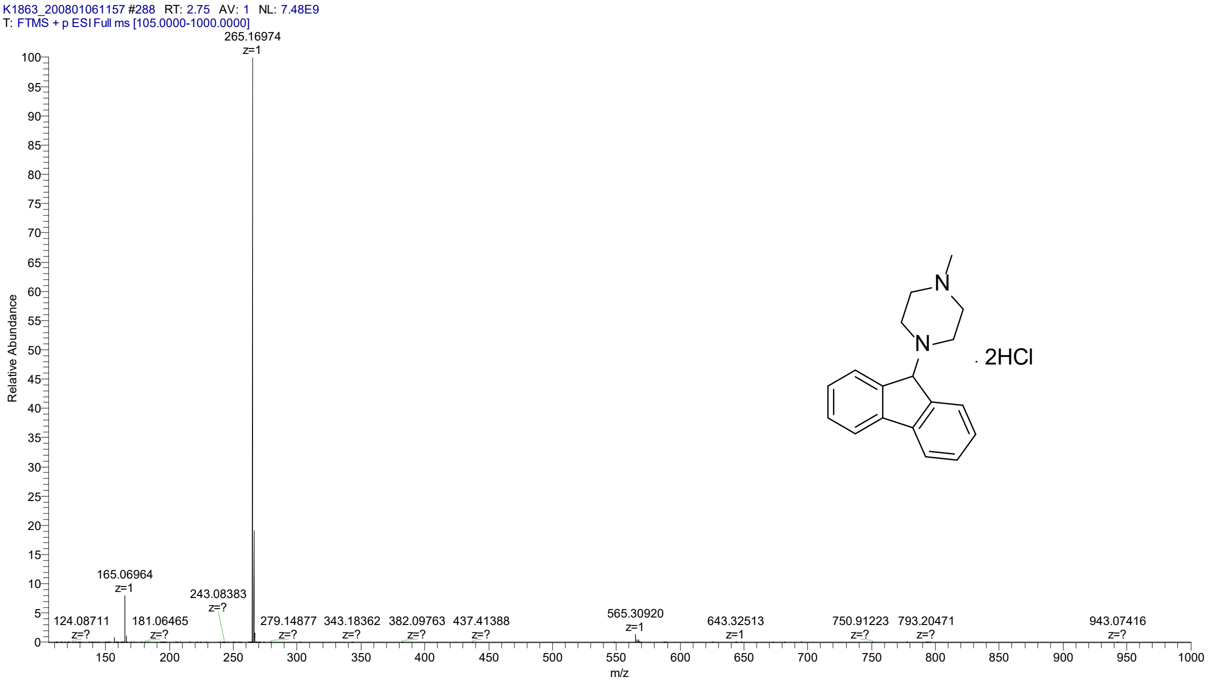

HPLC chromatogram (UV 254 nm) of 1-(9*H*-fluorene-9-yl)-4-ethylpiperazine hydrochloride (3k):

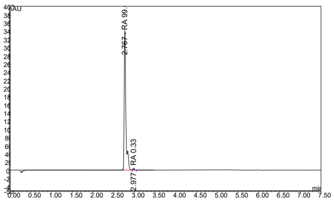

MS spectrum of 1-(9*H*-fluorene-9-yl)-4-ethylpiperazine hydrochloride (3k) at R_t: 2.77 min:

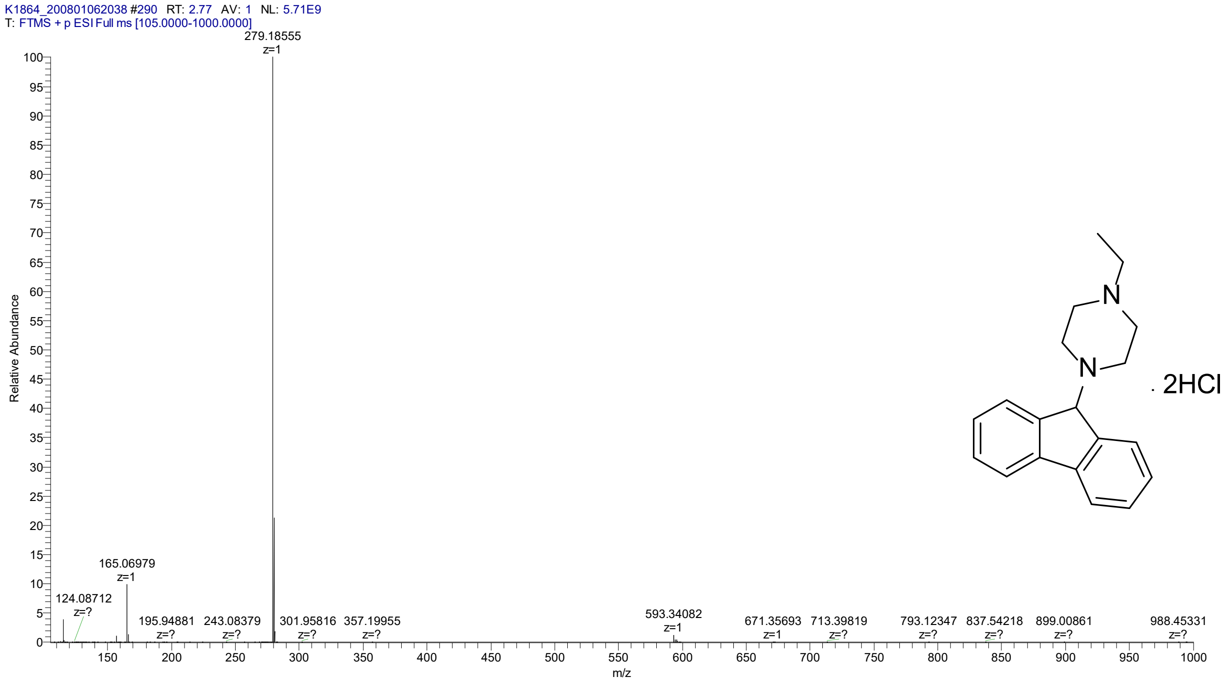

HPLC chromatogram (UV 254 nm) of 4-(9*H*-fluorene-9-yl)morpholine hydrochloride (3l):

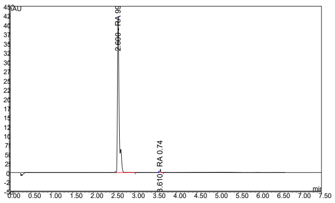

MS spectrum of 4-(9*H*-fluorene-9-yl)morpholine hydrochloride (3l) at R_t: 2.60 min:

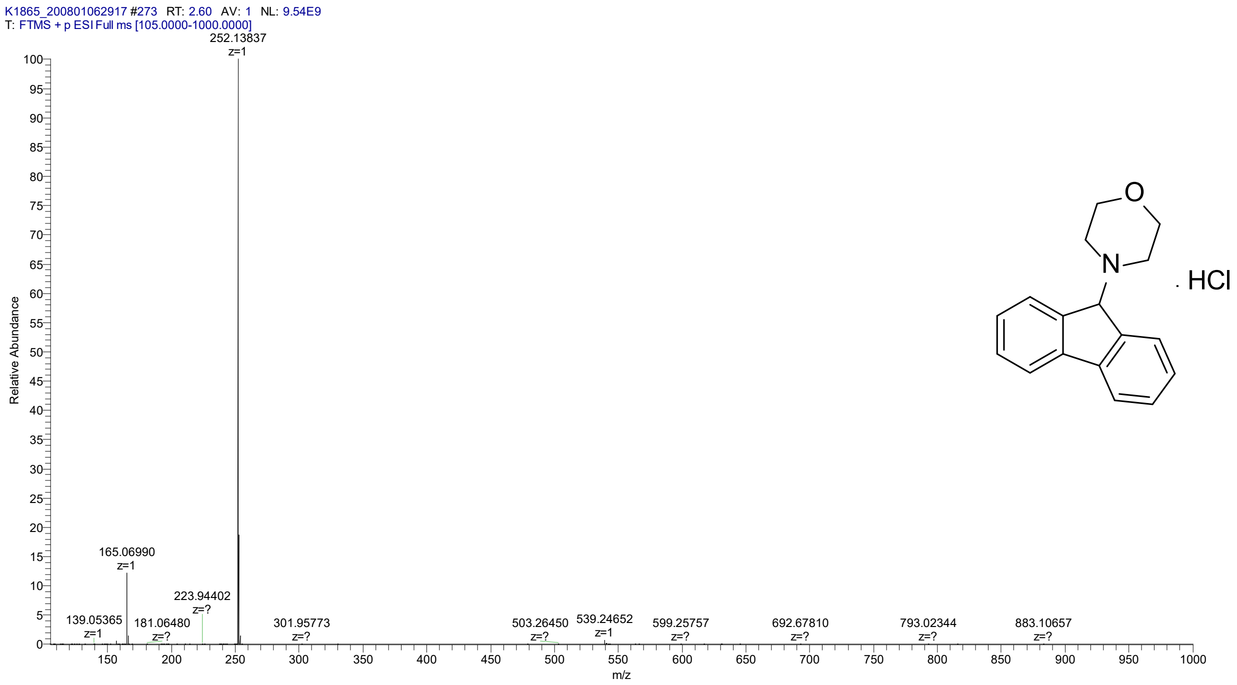

HPLC chromatogram (UV 254 nm) of *N*-butyl-9*H*-fluoren-9-amine hydrochloride (3m):

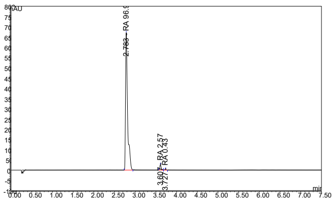

MS spectrum of *N*-butyl-9*H*-fluoren-9-amine hydrochloride (3m) at R_t: 2.81 min:

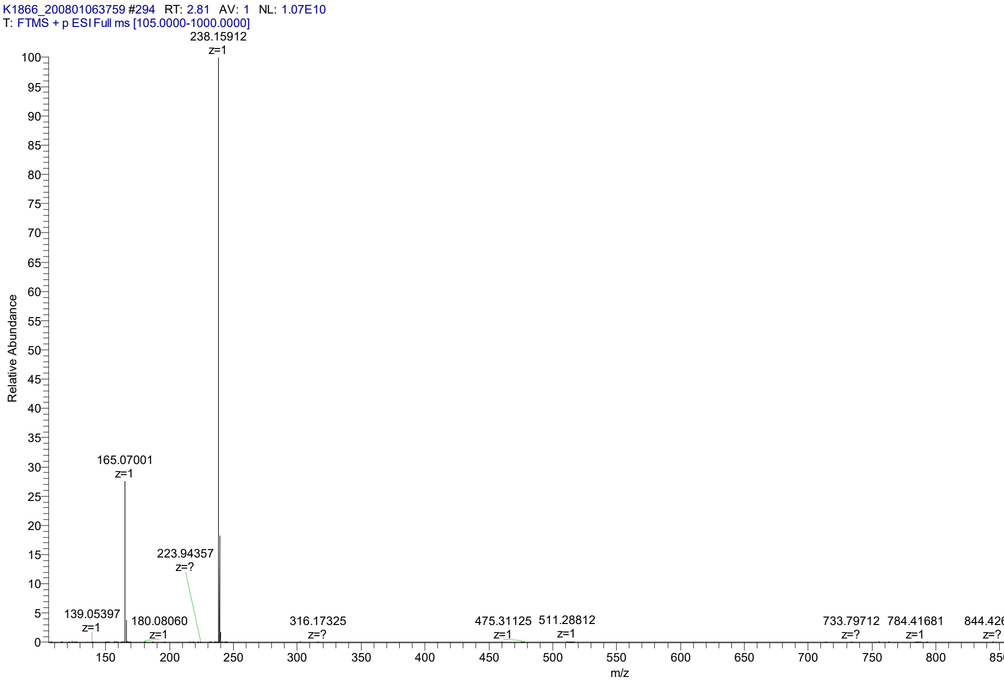

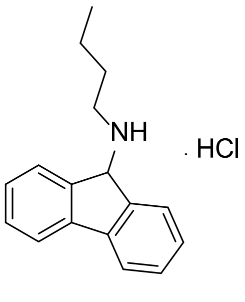

HPLC chromatogram (UV 254 nm) of *N*-(2-methylpropyl)-9*H*-fluoren-9-amine hydrochloride (3n):

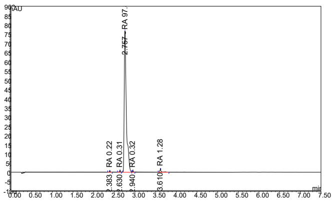

MS spectrum of *N*-(2-methylpropyl)-9*H*-fluoren-9-amine hydrochloride (3n) at R_t: 2.76 min:

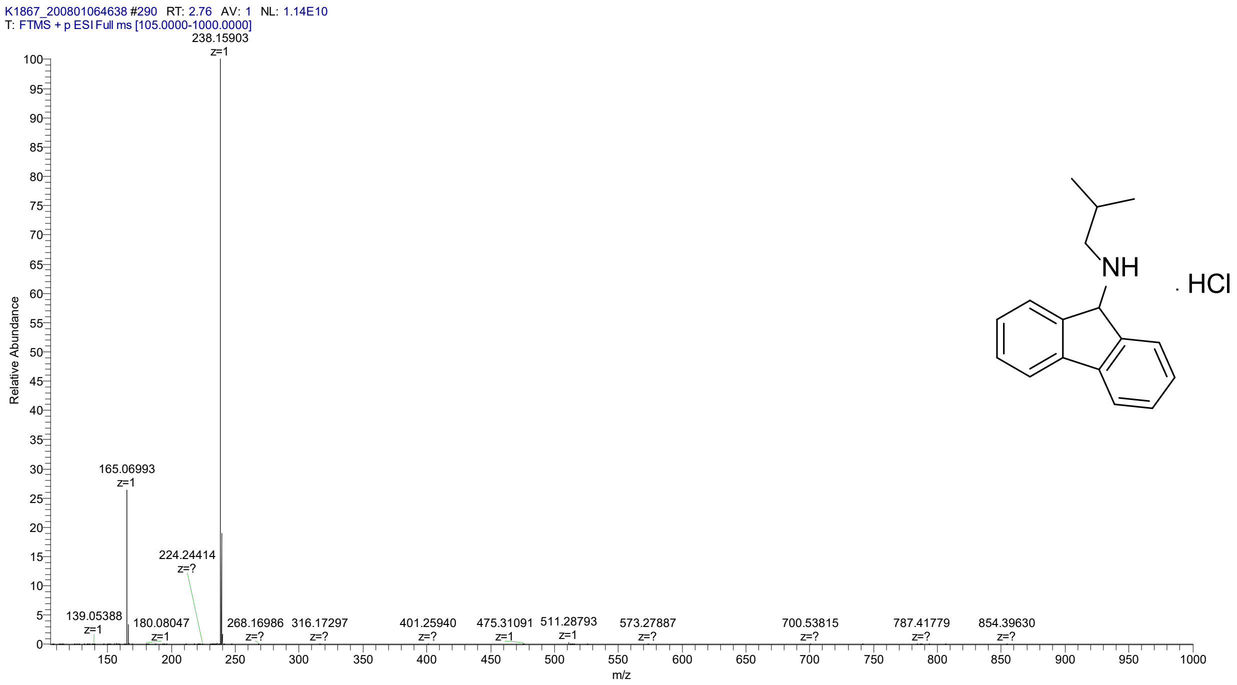

HPLC chromatogram (UV 254 nm) of 1-(9*H*-fluoren-9-yl)pyrrolidine hydrochloride (3o):

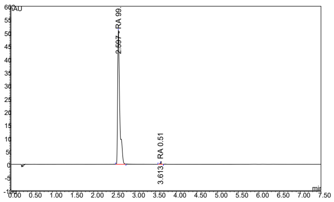

MS spectrum of 1-(9*H*-fluoren-9-yl)pyrrolidine hydrochloride (3o) at R$_t$: 2.61 min:

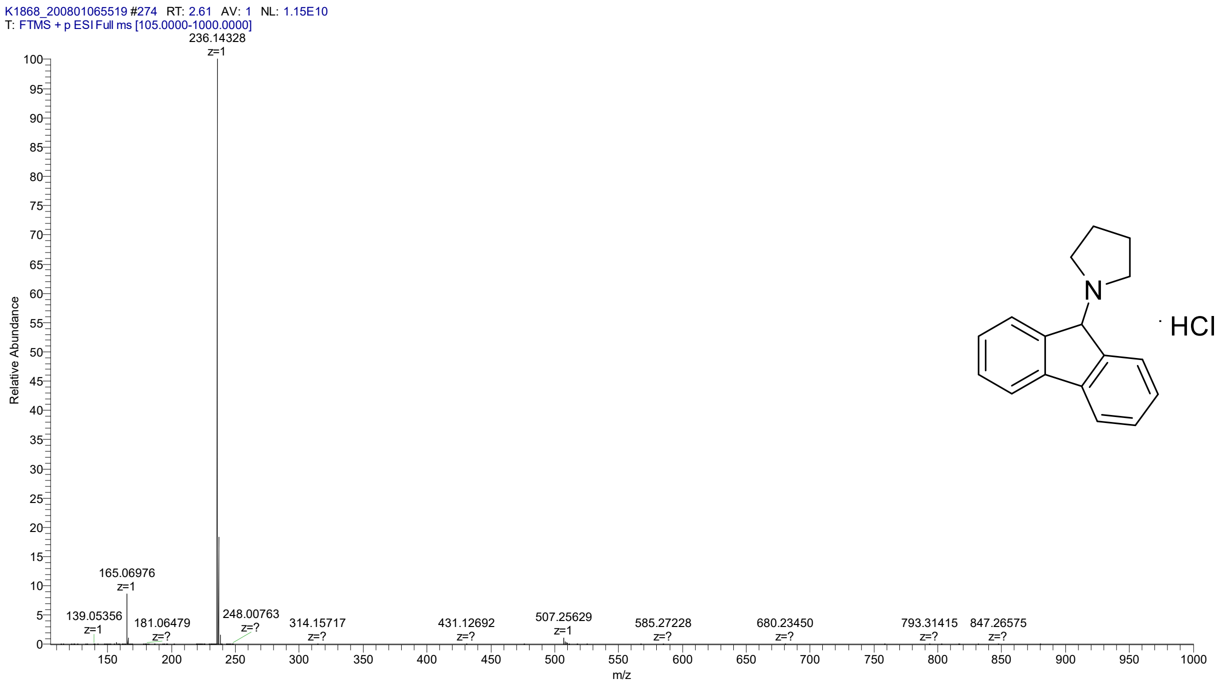

5. References

1. Daina, A.; Michielin, O.; Zoete, V. SwissADME: a free web tool to evaluate pharmacokinetics, drug-likeness and medicinal chemistry friendliness of small molecules. *Scientific Reports* **2017**, *7*, 42717, doi:10.1038/srep42717.
2. Lipinski, C.A. Lead- and drug-like compounds: the rule-of-five revolution. *Drug Discovery Today. Technologies* **2004**, *1*, 337–341, doi:10.1016/j.ddtec.2004.11.007.
3. Ghose, A.K.; Viswanadhan, V.N.; Wendoloski, J.J. A knowledge-based approach in designing combinatorial or medicinal chemistry libraries for drug discovery. 1. A qualitative and quantitative characterization of known drug databases. *J Comb Chem* **1999**, *1*, 55–68, doi:10.1021/cc9800071.
4. Veber, D.F.; Johnson, S.R.; Cheng, H.-Y.; Smith, B.R.; Ward, K.W.; Kopple, K.D. Molecular properties that influence the oral bioavailability of drug candidates. *J Med Chem* **2002**, *45*, 2615–2623, doi:10.1021/jm020017n.
5. Egan, W.J.; Merz, K.M.; Baldwin, J.J. Prediction of drug absorption using multivariate statistics. *J Med Chem* **2000**, *43*, 3867–3877, doi:10.1021/jm000292e.
6. Muegge, I.; Heald, S.L.; Brittelli, D. Simple selection criteria for drug-like chemical matter. *J Med Chem* **2001**, *44*, 1841–1846, doi:10.1021/jm015507e.
